# Supplementary material for: When has service provision for transient ischaemic attack improved enough? A discrete event simulation economic modelling study
Source: BMJ Open. 2017 Nov 25;7(11):e018189. doi: 10.1136/bmjopen-2017-018189 (PMC5719325; doi:10.1136/bmjopen-2017-018189)
Supplement: Supplementary file 1 [file bmjopen-2017-018189supp001.pdf]

Online data supplement

Contents

1. **Extended methods**
2. **eFigure 1.** Care pathway for patients presenting with symptoms of transient ischemic attack
3. **eTable 1.** Sources of data included in the model
4. **eTable 2.** Resource unit costs included in the service provision model
5. **eTable 3.** Costs, resource utilisation and outcomes (per year) of modifying TIA service provision in hospital 2.
6. **References**

Extended methods

Setting and service design

The model was designed to assess the costs and clinical consequences arising from patients suspected of suffering an acute TIA and referred to two large urban hospitals in the West Midlands region of the UK. Both hospitals ran a specialist TIA outpatient service catering for approximately 500 patients with suspected stroke and TIA every year. The model parameters were defined by the characteristics of individuals attending these services and recruited to an observational study[1] during 2011 (table 1).

The availability of specialist TIA clinics was modelled on the basis of existing service provision at participating hospital sites during the study period. Patients could either be seen in traditional outpatient clinics, admitted, or seen on the ward on an outpatient basis. The original services were designed within the confines of available clinical staff and a lack of specialist cover at the weekends. Services were redesigned at each site during the study period and the impact of these changes was examined by modelling both the original and modified service. The number and distribution of clinics in each service are described in Table 2.

The impact of further adjustments to the modified service at each site was modelled to replicate the following scenarios:

a) The addition of high risk clinic slots for patients presenting on a Saturday and Sunday
b) Including a weekend service but reducing the number of routine weekday clinics, by up to 5 clinic slots (one patient per slot) per week.

Overview of the model

A discrete event simulation model was programmed in Delphi version 4 (Borland, San Francisco, CA, USA). It was adapted from a previously developed model and further detail regarding the general modelling framework can be found in the original report.[2] Briefly, the model generated a number of possible (virtual) patient histories which began at the onset of TIA (or TIA like) symptoms and followed the patient along the clinical pathway from initial presentation to referral, specialist TIA clinic attendance, treatment and lifetime follow-up for subsequent stroke morbidity and mortality. An essential feature of the model was that patients were sharing limited resources in the form of routine clinics.

The model estimated the impact of existing service provision, the effect of recent service change and the impact of further alterations to clinic availability on service costs/provision and subsequent stroke events, from a health services perspective.

Clinical pathways in the model

Following onset of an initial event, patients were assumed to contact either their general practitioner (GP) or attend the emergency department (ED) (eFigure 1, online supplemental material). The probability that a patient would choose a specific route was dependent on the type of patient (high/low risk), the time of day, and day of week, estimated using data from the Oxford Vascular Study.[3] Time taken to reach the service depended on the route chosen; where patients directly attended the ED, the time was sampled from a Weibull distribution fitted to data from the Newcastle Stroke Service.[2] Patients attending their GP were assumed to arrive within daytime clinic hours.[3] Following this initial contact with a healthcare professional, patients were referred to a specialist TIA outpatient clinic, seen on the ward (as an outpatient) or admitted as an inpatient. The type of referral was dependant on the specific hospital service provision, clinic availability and the level of risk of the attending patient, defined according to the $ABCD^2$ score.[4] During clinic attendance, it was assumed that the appropriate treatment would be initiated (i.e. blood pressure lowering, cholesterol lowering or antiplatelet therapy in accordance with guidelines) and a small proportion of patients (4.1%) would be deemed suitable for carotid endarterectomy.[5,6] Patients were assumed to take this treatment as prescribed and gain the full benefit in terms of stroke prevention.

Model population

All patients in the model were hypothetical, although they were based on data from real patients and hospitals. Overall rates of patient presentation were based on initial runs of the model and ensured a realistic distribution of final diagnoses which represented the study sample, previous literature[7] and expert opinion (60% TIA mimics, 33% genuine TIA, and 7% minor stroke). High and low risk TIAs were defined according to the $ABCD^2$ score.[4] In the absence of robust data for the distribution of new referrals through the week, it was assumed that new cases arrived uniformly.

Due to missing risk data in the sample population, the observed ratio of low risk/high risk TIA patients used as basecase in the model was supplemented in sensitivity analyses by estimates derived from those reported in previous studies[4,8,9] and the experience of stroke physicians from centres across the UK [personal communications, D Sims, G Ford and C Roffe]. The range of these estimates tested in this sensitivity analysis for impact on the model results was: 2.5:1 (high:low risk; base-case) to 1:1, 5:1 and 7:1.[4,8,9]

Follow-up and risk of repeat events

Hypothetical patients remained in the model until one year from symptom onset, after which the increased risk of repeat event returns close to normal,[10,11] unless they died or suffered a non-fatal disabling stroke. No distinction was made in the model between fatal strokes and non-fatal disabling strokes: these were labelled "major strokes". The risk of a repeat event (TIA or stroke) was dependent on the type of initial event (minor stroke, true TIA or mimic), the $ABCD^2$ based risk prediction of a subsequent event and other relevant risk factors such as age, presence of atrial fibrillation and medication prescribed.[4,6,12-17] Following a minor (non-disabling) stroke, patients remained in the model, but with an additional risk of mortality that could be reduced by appropriate treatment. Additional deaths from this cause were estimated and labelled in the model outputs as "post-stroke deaths". Modelled outputs are therefore derived from risk profile of the hypothetical patients, adjusting for the effects of treatments which would start at varying times in the different scenarios. Risks were modelled using a Weibull distribution for the time to event which allowed for a substantially increased risk in the short term, followed by a decreasing risk over time.[18] Examples of the modelled risk for two patients (with high and low risk characteristics) are given in figure 1.

The model was run 100 times for a total simulated time of 12 years in each run: the first year of the run was regarded as a "warm up" period to allow the system to reach a steady state before collecting data. Patients entering during the next 10 years were included in the model results, with the final year being necessary to allow for a one-year follow up time for all included patients. Results for patients entering the model during this last "follow up" period were not included, but such patients were modelled to ensure that the constraints on clinic availability were maintained. Results are presented in terms of annual costs and other outcomes.

Costs and outcomes

The model included costs of any GP visit (from presentation to referral), or transport by ambulance to the ED and ED attendance, outpatient clinics or hospital admission for TIA and stroke, surgery and therapy (eTables 1 and 2, online data supplement). Pre-hospital and treatment related costs were the same for all options considered in this study since the focus was to compare comparative differences between modelled services caused by different clinic configurations. Costs included in the model were taken from a combination of NHS reference costs,[19,20] and drug costs from the British National Formulary.[21] The price year for all costs was 2011-12.

The primary outcome was the number of expected major strokes occurring post TIA, based on risk analysis. Secondary outcomes included the overall service costs per year and attainment of national

targets for TIA service provision. Target attainment was defined as the number of high and low risk 'breaches' which occurred in each service per year: high risk breaches were defined as a high risk patient not seen by a specialist within 24 hours of initial referral.[5,22] Low risk breaches were defined as low risk patients not seen by a specialist within seven days of referral.[5,22] Further outcomes examined the median time from referral to specialist appointment, the total number of routine outpatient appointments available (used or unused) and any unscheduled outpatient appointments required (where high risk patients were assessed immediately on the ward).

All data are presented as means or medians ± standard deviation (SD), inter-quartile range (IQR) or 99% confidence intervals (CI), chosen because multiple values are compared. Percentages are given for the total population unless otherwise stated.

eFigure 1. Care pathway for patients presenting with symptoms of transient ischemic attack

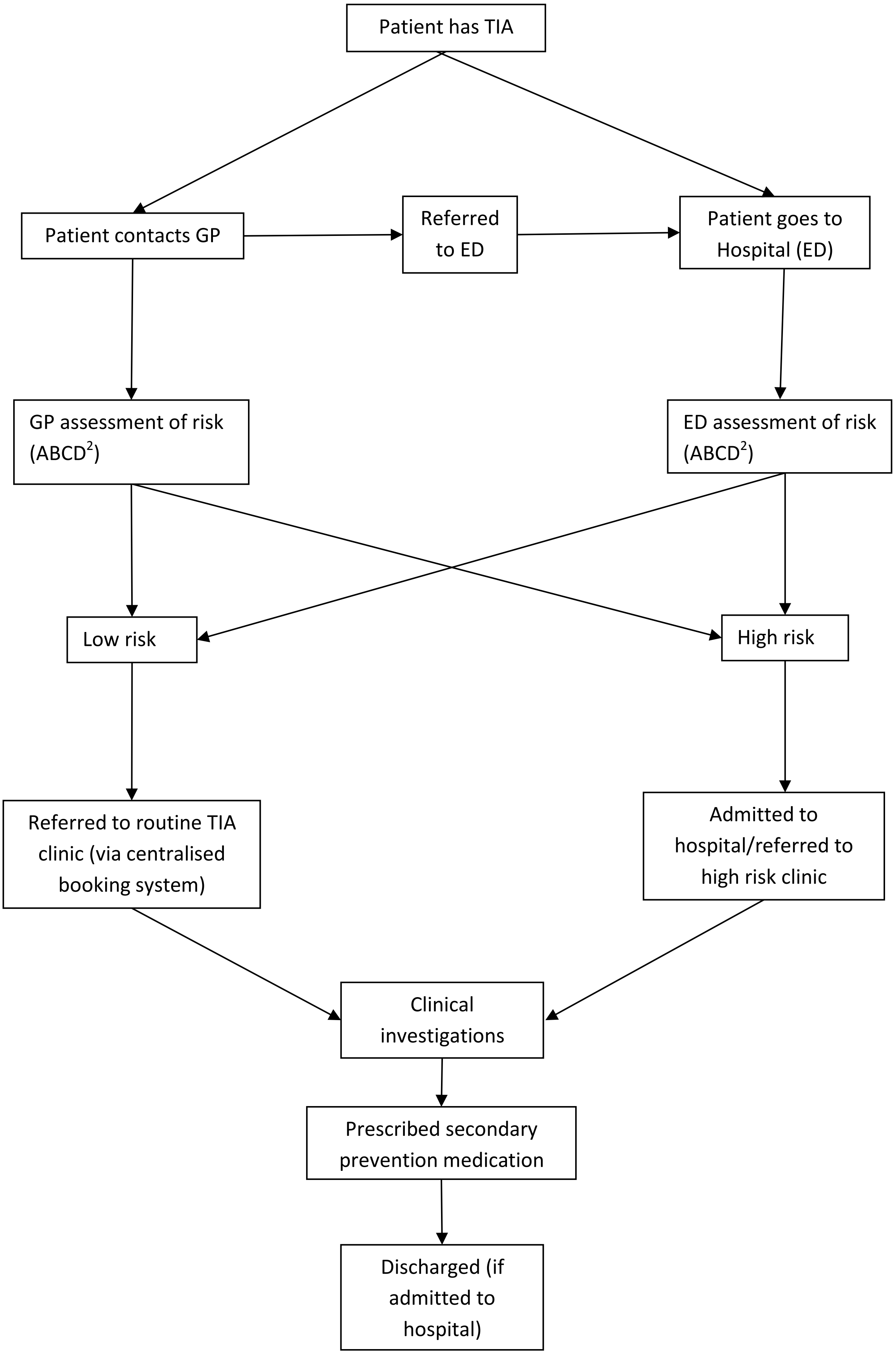

eTable 1. Sources of data included in the model

Model Inputs	Source	References
Schedule of Appointments<br>Casemix (mimic, TIA, minor stroke)	Participating hospital sites, Previous literature and the experience of stroke physicians from centres across the UK (personal communications, D Sims, G Ford and C Roffe)	8,9
Distribution of patient ages	Data from the OXVASC study adjusted to England and Wales population	23
Patient characteristics (gender, components of $ABCD^2$ score, current medication, AF status, cholesterol level, diabetes conditional on age as appropriate)	Data from the CLAHRC optimisation of the management of stroke and TIA study	1
Patient choice to contact GP or ambulance	OXVASC data	3
Time for patient to reach GP	Assumptions sustained from SDO model	2
Time for patient to reach service using ambulance	Data from Northumbria Ambulance Service	2
"Other cause" death rates	Government Actuary’s Department & ONS, Health Statistics Quarterly	24-26
Baseline risk of repeat events	OXVASC data	23
Factors affecting risk of repeat events	Previous literature	12-17,23

OXVASC = OXford VASCular study; CLAHRC = Collaborations for Leadership in Applied Health Research and Care; GP = General Practitioner; $ABCD^2$ = Age, Blood pressure, Clinical features, Duration and Diabetes; AF = Atrial Fibrillation

eTable 2. Resource unit costs included used in the service provision model.

Item	Cost (£)	Notes	Data source (reference)
Use of an ambulance	230		[19]
General practitioner consultation	33		[20]
Emergency department consultation	143	Category 2 investigation not leading to admitted	[19]
Outpatient appointment	212		[19]
Repeat major stroke	3127		[19]
Assessment for carotid endarterectomy	50	Additional cost of ultrasound only	[19]
Carotid endarterectomy surgery	3923		[19]
Stroke after carotid endarterectomy	3127	As for repeat stroke – small numbers	[19]
Hospital Admission	413		[19]
MRI	92	Applied to 20% of patients at outpatient appointment or hospital admission	[19]
Initiation of antiplatelet therapy*	0.485	300mg loading (from pack of 56 aspirin)	[21]
Antiplatelet mono therapy (per week)*	0.1213	75mg daily from pack of 56 aspirin	[21]
Antiplatelet dual therapy (per week)*	1.0683	Mono plus clopidogrel 75 mg od	[21]
Warfarin (per week)*	0.2150	3mg daily	[21]
Statin (per week)*	0.2925	Simvastatin 40mg daily (from pack of 28)	[21]
Antihypertensive*	0.2025	Bendrofulmethiazide 2.5mg daily	[21]

*Costs taken from the British National Formulary (November 2012) [21]

eTable 3. Costs, resource utilisation and outcomes (per year) of modifying TIA service provision in hospital 2.

	**Original service (12 clinic slots per week)**	**Modified service (14 clinic slots per week)**	**New service (13 clinic slots per week)**	**New service (11 clinic slots per week)**	**New service (9 clinic slots per week)**
Days operating per week	7 days	7 days	7 days	7 days	7 days
Total number of patients presenting	490	490	490	490	490
Cost of clinics used & unused	£368,000	£363,000	£353,000	£329,000	£304,000
Major strokes post TIA (mean, 99% CI)*	10.7 (10.5-10.9)	10.4 (10.2-10.5)	10.7 (10.5-10.9)	10.6 (10.5-10.8)	10.6 (10.4-10.8)
Post-stroke deaths (mean, 99% CI)*	3.0 (2.9-3.1)	3.0 (2.9-3.1)	3.0 (2.9-3.1)	3.1 (3.0-3.2)	3.0 (2.9-3.1)
Number of high risk breaches† (%)	0 (0.0%)	0 (0.0%)	0 (0.0%)	0 (0.0%)	0 (0.0%)
Number of low risk breaches‡ (%)	0 (0.0%)	0 (0.0%)	0 (0.0%)	1 (0.3%)	44 (12.9%)
Time from referral to clinic appointment for high risk patients in days (median, IQR)	0.85 (0.74-0.94)	0.75 (0.08-0.88)	0.75 (0.08-0.88)	0.75 (0.08-0.88)	0.75 (0.08-0.88)

TIA = transient ischemic attack; CI = confidence intervals; IQR = inter-quartile range

Of the ~490 patients in the model, 340 are considered low risk (294 TIA mimic; 46 low risk TIA) and 150 are considered high risk (116 high risk TIA; 34 minor stroke)

*Point estimate and 99% quasi confidence interval reflecting the uncertainty from sampling in the model, not any uncertainty in model parameters. 99% was chosen because of multiple values were compared

†High risk breaches were defined as high risk patients not seen by a specialist within 24 hours of initial clinic referral. These could not occur with the service pattern, as all high risk patients were admitted in the original service, and seen on the ward in the modified service.

‡ Low risk breaches were defined as low risk patients not seen by a specialist within 7 days of initial clinic referral.

References

1. Sheppard JP, Mellor RM, Bailey SM, et al. Protocol for an observation and implementation study investigating optimisation of the management of stroke and transient ischaemic attack (TIA). *BMJ open* 2012; **2**(3).

2. Mant J, Barton P, McManus RJ, Rothwell PM. What is the Optimum Model of Service Delivery for Transient Ischaemic Attack?: National Co-ordinating Centre for NHS Service Delivery and Organisation R&D (NCCSDO), 2008.

3. Lasserson DS, Chandratheva A, Giles MF, Mant D, Rothwell PM. Influence of general practice opening hours on delay in seeking medical attention after transient ischaemic attack (TIA) and minor stroke: prospective population based study. *BMJ (Clinical research ed)* 2008; **337**: a1569.

4. Rothwell PM, Giles MF, Flossmann E, et al. A simple score (ABCD) to identify individuals at high early risk of stroke after transient ischaemic attack. *Lancet* 2005; **366**(9479): 29-36.

5. National Institute for Health and Clinical Excellence (NICE). Diagnosis and intial management of acute stroke and transient ischemic attack (TIA), 2008.

6. Rothwell PM, Eliasziw M, Gutnikov SA, Warlow CP, Barnett HJ. Endarterectomy for symptomatic carotid stenosis in relation to clinical subgroups and timing of surgery. *Lancet* 2004; **363**(9413): 915-24.

7. Brazzelli M, Shuler K, Quayyum Z, et al. Clinical and imaging services for TIA and minor stroke: results of two surveys of practice across the UK. *BMJ open* 2013; **3**(8).

8. Tsivgoulis G, Stamboulis E, Sharma VK, et al. Multicenter external validation of the ABCD2 score in triaging TIA patients. *Neurology* 2010; **74**(17): 1351-7.

9. Asimos AW, Johnson AM, Rosamond WD, et al. A multicenter evaluation of the ABCD2 score's accuracy for predicting early ischemic stroke in admitted patients with transient ischemic attack. *Annals of emergency medicine* 2010; **55**(2): 201-10.e5.

10. Coull AJ, Lovett JK, Rothwell PM. Population based study of early risk of stroke after transient ischaemic attack or minor stroke: implications for public education and organisation of services. *BMJ (Clinical research ed)* 2004; **328**(7435): 326.

11. Lovett JK, Dennis MS, Sandercock PA, Bamford J, Warlow CP, Rothwell PM. Very early risk of stroke after a first transient ischemic attack. *Stroke; a journal of cerebral circulation* 2003; **34**(8): e138-40.

12. Collaborative meta-analysis of randomised trials of antiplatelet therapy for prevention of death, myocardial infarction, and stroke in high risk patients. *BMJ (Clinical research ed)* 2002; **324**(7329): 71-86.

13. Aguilar MI, Hart R. Oral anticoagulants for preventing stroke in patients with non-valvular atrial fibrillation and no previous history of stroke or transient ischemic attacks. *The Cochrane database of systematic reviews* 2005; (3): Cd001927.

14. Baigent C, Keech A, Kearney PM, et al. Efficacy and safety of cholesterol-lowering treatment: prospective meta-analysis of data from 90,056 participants in 14 randomised trials of statins. *Lancet* 2005; **366**(9493): 1267-78.

15. Ebrahim S, Harwood R. Stroke epidemiology, evidence, and clinical practice. Oxford University Press: Oxford; 1999.

16. MacMahon S. BP and the risks of CV disease. In: Swales J, ed. Textbook of hypertension. Oxford: Blackwell Scientific Publications; 1994.

17. Wolf PA, Abbott RD, Kannel WB. Atrial fibrillation as an independent risk factor for stroke: the Framingham Study. *Stroke; a journal of cerebral circulation* 1991; **22**(8): 983-8.

18. Rothwell PM, Giles MF, Chandratheva A, et al. Effect of urgent treatment of transient ischaemic attack and minor stroke on early recurrent stroke (EXPRESS study): a prospective population-based sequential comparison. *Lancet* 2007; **370**(9596): 1432-42.

19. Department of Health. 2011–12 Reference Costs. 2013. https://www.gov.uk/government/publications/2011-12-reference-costspublication (accessed June 2013).

20. Curtis L. Unit Costs in Health and Social Care 2011. 2012. http://www.pssru.ac.uk/project-pages/unit-costs/2011/index.php. (accessed November 2012).

21. British National Formulary (BNF). Pharmaceutical Press. 2012. https://www.bnf.org/ (accessed November 2012).

22. Intercollegiate Stroke Working Party. National clinical guideline for stroke. London: Royal College of Physicians, 2012.

23. Rothwell PM, Coull AJ, Giles MF, et al. Change in stroke incidence, mortality, case-fatality, severity, and risk factors in Oxfordshire, UK from 1981 to 2004 (Oxford Vascular Study). *Lancet* 2004; **363**(9425): 1925-33.

24. Government Actuary's Department. Interim life tables. http://www.gad.gov.uk/, 2006.

25. Health Statistics Quarterly. Deaths by age, sex and underlying cause, 2005 registrations. http://www.statistics.gov.uk/statbase/, 2007.

26. Office for National Statistics. Interim Life Tables for England. http://www.ons.gov.uk/ons/publications/index.html, 2011.
