# Supplementary material for: When has service provision for transient ischaemic attack improved enough? A discrete event simulation economic modelling study
Source: BMJ Open. 2017 Nov 25;7(11):e018189. doi: 10.1136/bmjopen-2017-018189 (PMC5719325; doi:10.1136/bmjopen-2017-018189)
Supplement: Author's manuscript [file bmjopen-2017-018189.draft_revisions.pdf]

BMJ Open is committed to open peer review. As part of this commitment we make the peer review history of every article we publish publicly available.

When an article is published we post the peer reviewers' comments and the authors' responses online. We also post the versions of the paper that were used during peer review. These are the versions that the peer review comments apply to.

The versions of the paper that follow are the versions that were submitted during the peer review process. They are not the versions of record or the final published versions. They should not be cited or distributed as the published version of this manuscript.

BMJ Open is an open access journal and the full, final, typeset and author-corrected version of record of the manuscript is available on our site with no access controls, subscription charges or pay-per-view fees (http://bmjopen.bmj.com).

If you have any questions on BMJ Open's open peer review process please email editorial.bmjopen@bmj.com

BMJ Open

When has service provision for transient ischemic attack improved enough? A discrete event simulation economic modelling study

Journal:	*BMJ Open*
Manuscript ID	bmjopen-2017-018189
Article Type:	Research
Date Submitted by the Author:	13-Jun-2017
Complete List of Authors:	Barton, Pelham; University of Birmingham, Health Economics Unit<br>Sheppard, James; University of Oxford, Nuffield Department of Primary Care Health Sciences<br>Penaloza, Cristina; University of Birmingham, School of Health and Population Sciences<br>Jowett, Sue; University of Birmingham, Health Economics Unit<br>Ford, Gary; Oxford University Hospitals NHS Foundation Trust, Oxford Academic Health Science Network<br>Lasserson, Daniel; Oxford University Hospitals NHS Foundation Trust, NIHR Oxford Biomedical Research Centre<br>Mant, Jonathan; University of Cambridge, General Practice and Primary Care Research Unit<br>Mellor, Ruth; NHS Lanarkshire, Department of Public Health<br>Quinn, Tom; St George's Univesity of London & Kingston University, Joint Faculty of Health, Social Care & Education<br>Rothwell, Peter; Stroke Prevention Research Unit, Nuffield Department of Clinical Neurosciences<br>Sandler, David; Heart of England NHS Foundation Trust,<br>Sims, Don; University Hospitals Birmingham NHS Foundation Trust, Stroke Medicine<br>McManus, Richard; University of Oxford, Dept of Primary Care Health Sciences
**Primary Subject Heading**:	Health economics
Secondary Subject Heading:	Cardiovascular medicine, Neurology
Keywords:	Economic modelling, Stroke < NEUROLOGY, Secondary Care, Secondary prevention, Health services

SCHOLARONE™
Manuscripts

When has service provision for transient ischemic attack improved enough? A discrete event simulation economic modelling study

Modelling changes to service provision for transient ischemic attack

Barton, P.,[1] Sheppard, J.P.,[2] Penaloza-Ramos, M.C.,[1] Jowett, S.,[1] Ford, G.A.,[3] Lasserson, D.,[4] Mant, J.,[5] Mellor, R.M.,[6] Quinn, T.,[7] Rothwell, P.M.,[8] Sandler, D.,[9] Sims, D.,[10] McManus, R.J.[2] on behalf of the BBC CLAHRC investigators

[1]Health Economics Unit, University of Birmingham, Edgbaston, Birmingham, UK

[2]Nuffield Department of Primary Care Health Sciences, NIHR School for Primary Care Research, University of Oxford, Oxford, Oxfordshire, UK

[3]Oxford Academic Health Science Network, Oxford, Oxfordshire, UK

[4]Nuffield Department of Medicine, University of Oxford, Oxford, Oxfordshire, UK

[5]Primary Care Unit, University of Cambridge, Cambridge, Cambridgeshire, UK,

[6]Department of Public Health, NHS Lanarkshire, Kirklands, Fallside Road, Bothwell, UK

[7]Faculty of Health, Social Care and Education, St George's, University of London & Kingston University, London, UK

[8]Nuffield Department of Clinical Neurosciences, University of Oxford, Oxfordshire, UK.

[9]Heart of England NHS Foundation Trust, Birmingham, West Midlands, UK

[10]University Hospitals Birmingham NHS Foundation Trust, Birmingham, West Midlands, UK

Corresponding Author: James Sheppard

Address: Nuffield Department of Primary Care Health Sciences, University of Oxford, Radcliffe Primary Care, Radcliffe Observatory Quarter, Woodstock Road, Oxford, OX2 6GG

Email: james.sheppard@phc.ox.ac.uk

For peer review only

Telephone: +44 (0)1865 617192

Fax: +44 (0)1865 289287

Word count: 2,984 (not including abstract, tables, figures or references)

Key words: Economic modelling, stroke, secondary care, secondary prevention, health services

For peer review only

For peer review only - http://bmjopen.bmj.com/site/about/guidelines.xhtml

Abstract

Objectives: The aim of this study was to examine the impact of transient ischaemic attack service modification in two hospitals on costs and clinical outcomes.

Design: Discrete event simulation model using data from routine electronic health records

Participants: Patients with suspected TIA were followed from symptom onset to presentation, referral to specialist clinics, treatment and subsequent stroke.

Interventions: The model examined previous and modified services (more same day clinics) and further hypothetical service reconfiguration (7-day service with less availability of clinics per day).

Outcome measures: The primary outcome of the model was the prevalence of disabling stroke after TIA. Secondary outcomes included service costs (including those of treating subsequent stroke) and time to treatment and attainment of national targets for service provision (proportion of high risk patients seen within 24 hours).

Results: The estimated costs of service provision for 490 patients per year at each site were £340,000 and £368,000 respectively. This resulted in 31% of high risk patients seen within 24 hours of referral (47/150) with a median time from referral to clinic attendance/treatment of 1.15 days (IQR 0.93-2.88). The costs associated with the modified and hypothetical services varied from -£5,000 to +£21,000 and target attainment was improved to 79% (118/150). However, the median time to clinic attendance was only reduced to 0.85 days (IQR 0.17-0.99) and thus no appreciable impact on the modelled incidence of disabling stroke was observed (10.7 per year, 99% CI 10.5-10.9 [original service] vs. 10.6 per year, 99% CI 10.4-10.8 [modified service]).

Conclusions: Reconfiguration of services for TIA is effective at increasing target attainment, but in those services which are already working efficiently (treating patients within 1-2 days), it has little estimated impact on clinical outcomes.

Word count: 282

For peer review only

Strengths and limitations of this study

- This is the first study to model the impact of routine service provision for patients suffering transient ischemic attack.
- The results are likely to be representative of the hospitals studied, but not necessarily other centres in the UK and across the world.
- Modelling allows estimation of service 'unknowns' which requires pre-programming and therefore simplification of certain service intricacies. Thus, the services modelled represent stylised versions of the actual services offered by participating hospitals.
- Costs relate to standard weekday services and it is not clear whether those actually incurred would be increased by provision of services at the weekend.

For peer review only

Background

Transient ischaemic attack (TIA) is common, with incidences of 15-83 patients per 100,000 population recorded across the world;[1-5] in the UK, it affects approximately 50 patients per 100,000 population.[6] TIA is important because it represents a significant risk factor for future stroke with around 8% suffering an event within 7 days, and 12% within a month without preventative therapy.[7 8]

Rapid recognition and treatment of TIA patients at high risk of having a subsequent stroke is important because simple interventions, such as early prescription of preventative medications, can substantially reduce the risk of stroke following TIA, and existing evidence suggests that earlier intervention maybe better.[9 10] In the UK, guidelines[11 12] recommend that patients at high risk of recurrent stroke (defined using the ABCD^2 score of $\geq$4)[10] are seen within 24 hours of symptom onset and all other patients are seen within 7 days. A recent UK audit suggests there has been significant improvement in attainment of these targets: 45% of high risk outpatients and 60% of inpatients now receive treatment on the same day as referral, compared with just 10% and 33% four years earlier.[13] This improved target attainment has been achieved through a variety of approaches, with some centres admitting more patients for assessment and treatment and others designing services with excess routine clinic capacity.[14]

Service reconfiguration remains ongoing in routine clinical practice and it is unclear what impact such changes have on service costs and clinical outcomes. This study used discrete event simulation modelling to assess the impact of TIA service reconfiguration in two large urban hospitals with different approaches to service provision.

Methods

An extended methods section can be found in the online data supplement.

For peer review only - http://bmjopen.bmj.com/site/about/guidelines.xhtml

Setting and service design

The model was designed to assess the costs and clinical consequences arising from patients suspected of suffering an acute TIA, referred to two large urban hospitals in the West Midlands region of the UK. Both hospitals ran a specialist TIA outpatient service catering for approximately 500 patients with suspected TIA and minor stroke every year. The model parameters were defined by the characteristics of individuals attending these services, recruited to an observational study[15] during 2011 (table 1).

The availability of specialist TIA clinics was modelled on the basis of existing service provision at participating hospital sites during the study period. Patients could either be seen in traditional outpatient clinics, admitted, or seen on the ward on an outpatient basis. The original services were designed within the confines of available clinical staff and in particular a lack of specialist cover at the weekends. Both services subsequently underwent redesign and the impact of these changes on cost and outcomes was examined by modelling both the original and modified service. The number and distribution of clinics in each service are described in table 2.

The impact of further hypothetical adjustments to the modified service at each site was modelled to replicate the following scenarios:

a) The addition of high risk clinic slots for patients presenting on a Saturday and Sunday
b) Including a weekend service but reducing the number of routine weekday clinics, by up to 5 clinic slots per week.

Overview of the model

A discrete event simulation model was programmed in Delphi version 4 (Borland, San Francisco, CA, USA). It was adapted from a previously developed model and further detail regarding the general modelling framework can be found in the original report.[16] Briefly, the model generated a

For peer review only - http://bmjopen.bmj.com/site/about/guidelines.xhtml

number of possible (virtual) patient histories which began at the onset of TIA (or TIA like) symptoms and followed the patient along the clinical pathway from initial presentation to follow-up for subsequent stroke morbidity and mortality. An essential feature of the model was that patients were sharing limited resources in the form of routine clinics.

Clinical pathways in the model

Following onset of an initial event, patients were assumed to contact either their general practitioner (GP) or attend the emergency department (ED) (eFigure 1, online supplemental material). The probability that a patient would choose a specific route was dependent on the type of patient (high/low risk), the time of day, and day of week, estimated using data from the Oxford Vascular Study.[17] Following this initial contact with a healthcare professional, patients were referred to a specialist TIA outpatient clinic, seen on the ward (as an outpatient) or admitted as an inpatient. The type of referral was dependant on the specific hospital service provision, clinic availability and the level of risk of the attending patient, defined according to the ABCD^2 score.[10] During clinic attendance, it was assumed that the appropriate treatment would be initiated (i.e. blood pressure lowering, cholesterol lowering or antiplatelet therapy in accordance with guidelines) and a small proportion of patients (4.1%) would be treated with carotid endarterectomy.[12 18]

Model population

Overall rates of patient presentation were based on initial runs of the model and ensured a realistic distribution of final diagnoses which represented the study sample, previous literature[14] and expert opinion (60% TIA mimics, 33% genuine TIA, and 7% minor stroke). The observed ratio of low risk/high risk TIA patients used in the base-case of the model was supplemented in sensitivity analyses by estimates derived from those reported in previous studies[10 19 20] and the experience of stroke physicians from centres across the UK [personal communications, D Sims, G Ford and C

For peer review only

Roffe]. The range of these estimates tested in this sensitivity analysis for impact on the model results was: 2.5:1 (high:low risk; base-case) to 1:1, 5:1 and 7:1.[10 19 20]

Follow-up and risk of repeat events

Patients remained in the model until one year from symptom onset or until they suffered a repeat event (within the same year), after which the increased risk of repeat event returns close to normal.[7 8] The risk of a repeat event (TIA or disabling stroke) was dependent on the initial event (true TIA or mimic), the severity of that event (measured by $ABCD^2$ score) and other relevant risk factors such as age, presence of atrial fibrillation and medication prescribed.[10 18 21-26] Risks were modelled using a Weibull distribution for the time to event which allowed for a substantially increased risk in the short term, followed by a decreasing risk over time.[9] Examples of the modelled risk for two patients (with high and low risk characteristics) are given in figure 1. The model was run 100 times for a total simulated time of 12 years in each run.

Costs and outcomes

The model included costs of GP visits, transport by ambulance to the ED, ED attendance, outpatient clinics, hospital admission for TIA and stroke, surgery and therapy (eTables 1 and 2, online data supplement). Costs included in the model were taken from a combination of NHS reference costs[27 28] and drug costs from the British National Formulary[29] and were modelled from a health services perspective. The price year for all costs was 2011-12.

The primary outcome was the number of disabling strokes occurring post TIA. Secondary outcomes included the overall costs of service provision per year and attainment of national targets for TIA service provision. Target attainment was defined as the number of high and low risk ‘breaches’ which occurred in each service per year: high risk breaches were defined as a high risk patient not seen by a specialist within 24 hours of initial referral.[11 12] Low risk breaches were defined as low

For peer review only - http://bmjopen.bmj.com/site/about/guidelines.xhtml
For peer review only

risk patients not seen by a specialist within seven days of referral.[11 12] Further outcomes examined the median time from referral to specialist appointment, the total number of routine outpatient appointments available (used or unused) and any unscheduled outpatient appointments required (where high risk patients were assessed immediately on the ward).

All data are presented as means or medians ± standard deviation (SD), inter-quartile range (IQR) or 99% confidence intervals (CI), chosen because multiple values are compared. Percentages are given for the total population unless otherwise stated.

Results

TIA service usage

The model estimated a total of 490 patients would be referred to specialist TIA clinics each year at each hospital site. Of these, approximately 34 (7%) patients would be considered to have minor stroke and 162 (33%) would subsequently be considered to have confirmed TIA: the numbers classified as high/low risk, were 116/46, a ratio of 2.5:1. All subsequent results were robust to adjustment of this high:low risk ratio in the sensitivity analyses.

Costs and outcomes of original TIA service provision

The baseline incidence of disabling strokes post TIA was 10.6 per year (99% CI 10.4-10.8) in hospital 1 and 10.7 per year (99% CI 10.5-10.9) in hospital 2. The cost of providing TIA services at both sites ranged from £340,000-£368,000 per year (table 3 and eTable 3). Services included the provision of 624-724 specialist week day clinic slots per year. In hospital 1, few high risk patients were seen within 24 hours of referral (47/150 patients [31%]) and the median time from referral to clinic appointment was 1.15 days (IQR 0.93-2.88). In hospital 2, where all high risk TIAs were admitted (a target attainment of 100%), the median time from referral to being seen and treated by a specialist was 0.85 days (IQR 0.74-0.94).

For peer review only

For peer review only - http://bmjopen.bmj.com/site/about/guidelines.xhtml

Impact of service modification

Observed service modification increased the number of high risk patients seen within 24 hours in hospital 1 (118/150; guideline target attainment of 79%), with increased service costs (increased from £344,000 to £366,000 per year) due to an increase in the number of unused routine clinic appointments (225 to 316 unused clinic slots per year) (figure 2). This increase in target attainment, resulted in a median time from referral to clinic appointment of 0.85 days (IQR 0.17-0.99); the incidence of disabling strokes post TIA remained largely unchanged (10.6 per year, 99% CI 10.4-10.8 [modified service] vs. 10.7 per year, 99% CI 10.5-10.9 [original service]). Similar findings were observed at the second hospital site where costs were marginally reduced by not admitting patients with high risk TIA (decrease from £368,000 to £363,000 per year) but there was no change in the number of patients being seen within 24 hours (150/150; guideline target attainment of 100%), median time from referral to clinic appointment (0.75 days, IQR 0.08-0.88 [modified service] vs. 0.85 days, IQR 0.17-0.99 [original service]) or incidence of disabling strokes post TIA (10.4 per year, 99% CI 10.2-10.4 [modified service] vs. 10.7 per year, 99% CI 10.5-10.9 [original service]).

Potential optimal service provision

Modelling the impact of further hypothetical service reconfiguration, including a seven day service, suggested that it was possible to reduce service costs and the number of unused clinic appointments and improve guideline target attainment, but this had little impact on the incidence of disabling stroke post TIA (table 3 and eTable 3). Figure 2 shows the trade-off between routine appointment provision, unscheduled appointments and high/low risk guideline breaches. Assuming unused appointments (pink), unscheduled appointments (in light blue), admissions (purple) and breaches (red [high risk breaches] and light red [low risk breaches]) are preferably avoidable outcomes of service provision, the optimal hypothetical service in hospital 1 involved a weekend service with approximately 17-18 routine clinic appointments per week. In hospital 2, where more unscheduled

For peer review only

appointments were incurred, the optimal hypothetical service involved a weekend service with 10-11 routine clinic slots distributed across the week on Mondays, Wednesdays and Fridays (with high risk patients being seen in unscheduled appointments on the ward on any day of the week if necessary).

Discussion

The aim of this study was to model the impact of TIA service reconfiguration in two large urban hospitals and establish the impact on clinical outcomes (disabling strokes), costs, and clinic usage. Reconfiguration of services was found to be effective and appropriate for reducing resource (clinic slot) waste, and improving guideline target attainment with variable effects on costs. However, in hospitals where services, despite poor target attainment, had minimal patient delays, such as those studied here, significant reconfiguration had little impact on modelled clinical outcomes such as disabling stroke post TIA. These findings suggest that clinicians and service coordinators should be cautious before initiating significant reconfiguration of services which are already seeing high risk patients within 1-2 days, unless there is an obvious potential to reduce costs, such as reducing admissions of high risk TIA patients. Whilst national targets for care are important, consideration should be given to revising them to maximise the benefits of attainment whilst not incentivising small changes that might increase costs for no measurable clinical benefit.

Strengths and limitations

The present study used local hospital data to describe the characteristics of the presenting population and thus the results are likely to be representative of the hospitals studied, but not necessarily generalizable to other centres in the UK and across the world. Despite this, guideline target attainment in hospital 1 (original service configuration) was similar to that reported nationally (31% vs. 37% nationally)[13] which suggests that this hospital was more likely representative of centres across the UK.

For peer review only - http://bmjopen.bmj.com/site/about/guidelines.xhtml

Modelling allows estimation of service ‘unknowns’, such as the number of unscheduled clinic appointments required to meet guideline targets for high risk patients. This requires pre-programming with specific rules and therefore simplification of certain service intricacies is necessary. For example, the process by which high risk patients were allocated unscheduled clinic appointments was formalised such that a patient referred by the ED physician was assumed to have been seen by a stroke specialist within two hours. However, patients referred by their GP were assumed to be seen by the stroke specialist on the ward at 10am the following morning or 5pm the same day, whichever was later (but within 24 hours of initial referral). In reality, services are often adapted in response to a specific set of circumstances such as clinic room availability and thus, the services modelled here can best be thought of as stylised versions of the actual services offered by the hospitals in question.

The costs modelled here were taken from standard NHS reference costs[27 28] and included investigations, treatment and the cost of staff attending patients during each appointment/admission. Costs relate to standard weekday services and it is not clear whether those actually incurred would be increased by provision of services at the weekend. It is therefore possible that the costs of providing a weekend service may have been underestimated in the present analyses.

Findings in the context of previous literature and implications for practice

Economic modelling has previously been used for determining the impact of service reconfiguration in acute stroke[30-33] but studies examining optimal strategies for TIA service provision are less common. Those that exist, focus on whether or not patients should be admitted or be seen as an outpatient.[16 34 35] It is generally accepted that patient admission for TIA is inefficient,[15, 23] and specialist outpatient services are required,[36] but there are a paucity of objective data describing the configuration of these services, limited to questionnaire surveys of stroke physicians[14 37] and the

For peer review only - http://bmjopen.bmj.com/site/about/guidelines.xhtml

Sentinel Stroke National Audit Programme (SSNAP) in the UK.[13] The latter represents hospitals nationally (across the UK) but has only a small focus on TIA service provision and relies on participating hospitals to collect their own data and perform quality checks, introducing the possibility of bias.

The primary finding of this study was that whilst service reconfiguration can significantly improve guideline target attainment and potentially reduce service costs, such changes have little impact on the estimated prevalence of subsequent stroke, when services are already working efficiently. In the present study, even the largest service reconfiguration (introduction of a 7 day service) only reduced the median time from patient referral to clinic attendance and treatment by 0.3 days (8 hours). This enabled a large number of patients to be seen within 24 hours, improving guideline target attainment but had little impact on stroke risk. The recommendations for a 'see and treat within 24 hours' target for high risk patients were originally based on data from the EXPRESS study,[9] which showed that those patients seen and treated within 24 hours of initial referral had significantly better outcomes than those patients who were not. However, in the original service to which this optimal strategy was compared, the average time from referral to clinic appointment was 3 days (IQR 2-5 days) and time to first treatment was 20 days (IQR 8-53). The improvement in time to initiation of treatment was therefore 20 days, compared to an improvement of 8 hours seen in the present study, which explains the very different results in terms of clinical outcome.

Recent results from SSNAP show that 45% of high risk outpatients and 60% of high risk inpatients are seen and treated within 24 hours of referral, suggesting there is plenty of room for improvement. However, with a median time to first clinic appointment (and treatment) for all patients of just 2 days, the present analyses would suggest further improvements in guideline target attainment may have little impact on disabling stroke following TIA.

For peer review only

The modified services examined and proposed here may require the capacity to accommodate unscheduled clinic appointments, which is likely to have an opportunity cost affecting the quality of other services running concurrently in the hospital and may result in increased costs with little tangible benefit. In particular, the ability to accommodate such appointments depends on the time of day at which the patient presents, the availability of specialist stroke physicians and scanning equipment required to make an accurate diagnosis, as well as the needs of competing services within the hospital (e.g. acute stroke services).

Conclusions

Reconfiguration of specialist services for TIA can be effective and appropriate for reducing costs, reducing the number of unused routine clinic appointments and increasing clinical guideline target attainment. However, where services are already working near optimal, such modification has little impact on clinical outcomes such as subsequent stroke. Clinicians and service coordinators should be cautious before initiating significant reconfiguration of services which are already seeing high risk patients within 1-2 days, as such changes may increase costs with little or no change in outcome.

For peer review only

Declarations

Ethics approval and consent to participate

Approval for this project was obtained from the National Research Ethics Service (NRES) Committee, London – Queen Square (reference; 09/H0716/71).

Data sharing

The datasets analysed during the current study are available from the corresponding author on reasonable request.

Competing interests

The authors declare that they have no competing interests

Funding

This work was supported by the National Institute for Health Research (NIHR) as part of the Collaborations for Leadership in Applied Health Research and Care (CLAHRC) programme for Birmingham and Black Country. JPS held a Medical Research Council (MRC grant number MR/K022032/1) Strategic Skills Postdoctoral Fellowship. RJMcM holds an NIHR Professorship.

Authors' contributions

RJMcM, JM, SJ and PB had the original idea and gained the funding for the project. PB undertook the economic analyses. JPS and RMM were responsible for the data collection and JPS wrote the first draft of the manuscript with RJMcM and PB. PB, JPS, MCP, SJ, GAF, DL, JM, RMM, TQ, PMR, DS, DSi and RJM contributed to protocol development, refined the manuscript and approved the final version. RJMcM is the guarantor.

Acknowledgements

For peer review only

For peer review only - http://bmjopen.bmj.com/site/about/guidelines.xhtml

We would like to thank Amun Boyal, Kelly Rosborough and the staff at both participating hospitals for their assistance with recruitment and data collection. Thanks to Sheila Bailey and Anita Martin for their administrative support and Primary Care Clinical Research and Trials Unit for building and maintaining the study database.

Birmingham and Black Country Collaborations for Leadership in Applied Health Research and Care investigators include:

Peter Carr, Heart of England NHS Foundation Trust

Sheila Greenfield, Primary Care Clinical Sciences, University of Birmingham

Brin Helliwell, Lay member of Steering Group

Christina Nand, Lay member of Steering Group

Norman Phillips, Lay member of Steering Group

Rob Scott, Birmingham and Midland Eye Centre

Satinder Singh, Primary Care Clinical Sciences, University of Birmingham

Matthew Ward, West Midlands Ambulance Service NHS Trust

References

1. Cancelli I, Janes F, Gigli GL, et al. Incidence of transient ischemic attack and early stroke risk: validation of the ABCD2 score in an Italian population-based study. *Stroke; a journal of cerebral circulation* 2011;42(10):2751-7. doi: 10.1161/strokeaha.110.612705 [published Online First: 2011/08/13]

2. Correia M, Silva MR, Magalhaes R, et al. Transient ischemic attacks in rural and urban northern Portugal: incidence and short-term prognosis. *Stroke; a journal of cerebral circulation* 2006;37(1):50-5. doi: 10.1161/01.STR.0000195209.26543.8f [published Online First: 2005/12/03]

For peer review only - http://bmjopen.bmj.com/site/about/guidelines.xhtml

3. Feigin VL, Shishkin SV, Tzirkin GM, et al. A population-based study of transient ischemic attack incidence in Novosibirsk, Russia, 1987-1988 and 1996-1997. *Stroke; a journal of cerebral circulation* 2000;31(1):9-13. [published Online First: 2000/01/08]

4. Fonseca PG, Weiss PA, Harger R, et al. Transient ischemic attack incidence in joinville, Brazil, 2010: a population-based study. *Stroke; a journal of cerebral circulation* 2012;43(4):1159-62. doi: 10.1161/strokeaha.111.645101 [published Online First: 2011/12/31]

5. Kleindorfer D, Panagos P, Pancioli A, et al. Incidence and short-term prognosis of transient ischemic attack in a population-based study. *Stroke; a journal of cerebral circulation* 2005;36(4):720-3. doi: 10.1161/01.STR.0000158917.59233.b7 [published Online First: 2005/02/26]

6. Rothwell PM, Coull AJ, Giles MF, et al. Change in stroke incidence, mortality, case-fatality, severity, and risk factors in Oxfordshire, UK from 1981 to 2004 (Oxford Vascular Study). *Lancet* 2004;363(9425):1925-33. doi: 10.1016/s0140-6736(04)16405-2 [published Online First: 2004/06/15]

7. Coull AJ, Lovett JK, Rothwell PM. Population based study of early risk of stroke after transient ischaemic attack or minor stroke: implications for public education and organisation of services. *BMJ (Clinical research ed)* 2004;328(7435):326. doi: 10.1136/bmj.37991.635266.44 [published Online First: 2004/01/28]

8. Lovett JK, Dennis MS, Sandercock PA, et al. Very early risk of stroke after a first transient ischemic attack. *Stroke; a journal of cerebral circulation* 2003;34(8):e138-40. doi: 10.1161/01.str.0000080935.01264.91 [published Online First: 2003/07/12]

9. Rothwell PM, Giles MF, Chandratheva A, et al. Effect of urgent treatment of transient ischaemic attack and minor stroke on early recurrent stroke (EXPRESS study): a prospective population-

For peer review only

based sequential comparison. *Lancet* 2007;370(9596):1432-42. doi: 10.1016/s0140-6736(07)61448-2 [published Online First: 2007/10/12]

10. Rothwell PM, Giles MF, Flossmann E, et al. A simple score (ABCD) to identify individuals at high early risk of stroke after transient ischaemic attack. *Lancet* 2005;366(9479):29-36. doi: 10.1016/s0140-6736(05)66702-5 [published Online First: 2005/07/05]

11. Intercollegiate Stroke Working Party. National clinical guideline for stroke. London: Royal College of Physicians, 2012.

12. National Institute for Health and Clinical Excellence (NICE). Diagnosis and intial management of acute stroke and transient ischemic attack (TIA). NICE clinical guideline, 2008.

13. Royal College of Physicians. Sentinel Stroke National Audit Programme (SSNAP) Acute Organisational Audit Report 2014, 2014.

14. Brazzelli M, Shuler K, Quayyum Z, et al. Clinical and imaging services for TIA and minor stroke: results of two surveys of practice across the UK. *BMJ open* 2013;3(8) doi: 10.1136/bmjopen-2013-003359 [published Online First: 2013/08/10]

15. Sheppard JP, Mellor RM, Bailey SM, et al. Protocol for an observation and implementation study investigating optimisation of the management of stroke and transient ischaemic attack (TIA). *BMJ open* 2012;2(3) doi: 10.1136/bmjopen-2012-001430 [published Online First: 2012/06/27]

16. Mant J, Barton P, McManus RJ, et al. What is the Optimum Model of Service Delivery for Transient Ischaemic Attack?: National Co-ordinating Centre for NHS Service Delivery and Organisation R&D (NCCSDO), 2008.

17. Lasserson DS, Chandratheva A, Giles MF, et al. Influence of general practice opening hours on delay in seeking medical attention after transient ischaemic attack (TIA) and minor stroke:

For peer review only

prospective population based study. *BMJ (Clinical research ed)* 2008;337:a1569. doi: 10.1136/bmj.a1569 [published Online First: 2008/09/20]

18. Rothwell PM, Eliasziw M, Gutnikov SA, et al. Endarterectomy for symptomatic carotid stenosis in relation to clinical subgroups and timing of surgery. *Lancet* 2004;363(9413):915-24. doi: 10.1016/s0140-6736(04)15785-1 [published Online First: 2004/03/27]

19. Tsivgoulis G, Stamboulis E, Sharma VK, et al. Multicenter external validation of the ABCD2 score in triaging TIA patients. *Neurology* 2010;74(17):1351-7. doi: 10.1212/WNL.0b013e3181dad63e [published Online First: 2010/04/28]

20. Asimos AW, Johnson AM, Rosamond WD, et al. A multicenter evaluation of the ABCD2 score's accuracy for predicting early ischemic stroke in admitted patients with transient ischemic attack. *Annals of emergency medicine* 2010;55(2):201-10.e5. doi: 10.1016/j.annemergmed.2009.05.002 [published Online First: 2009/06/27]

21. Collaborative meta-analysis of randomised trials of antiplatelet therapy for prevention of death, myocardial infarction, and stroke in high risk patients. *BMJ (Clinical research ed)* 2002;324(7329):71-86. [published Online First: 2002/01/12]

22. Aguilar MI, Hart R. Oral anticoagulants for preventing stroke in patients with non-valvular atrial fibrillation and no previous history of stroke or transient ischemic attacks. *The Cochrane database of systematic reviews* 2005(3):Cd001927. doi: 10.1002/14651858.CD001927.pub2 [published Online First: 2005/07/22]

23. Baigent C, Keech A, Kearney PM, et al. Efficacy and safety of cholesterol-lowering treatment: prospective meta-analysis of data from 90,056 participants in 14 randomised trials of statins. *Lancet* 2005;366(9493):1267-78. doi: 10.1016/s0140-6736(05)67394-1 [published Online First: 2005/10/11]

For peer review only

24. Ebrahim S, Harwood R. Stroke epidemiology, evidence, and clinical practice. Oxford University Press: Oxford 1999.

25. MacMahon S. BP and the risks of CV disease. In: Swales J, ed. Textbook of hypertension. Oxford: Blackwell Scientific Publications 1994.

26. Wolf PA, Abbott RD, Kannel WB. Atrial fibrillation as an independent risk factor for stroke: the Framingham Study. *Stroke; a journal of cerebral circulation* 1991;22(8):983-8. [published Online First: 1991/08/01]

27. Department of Health. 2011–12 Reference Costs 2013 [Available from: https://www.gov.uk/government/publications/2011-12-reference-costspublication accessed June 2013.

28. Curtis L. Unit Costs in Health and Social Care 2011: Personal Social Services Research Unit (PSSRU); 2012 [Available from: http://www.pssru.ac.uk/project-pages/unit-costs/2011/index.php. accessed November 2012.

29. British National Formulary (BNF). Pharmaceutical Press 2012 [Available from: https://www.bnf.org/ accessed November 2012.

30. Penaloza-Ramos MC, Sheppard JP, Jowett S, et al. Cost-effectiveness of optimizing acute stroke care services for thrombolysis. *Stroke; a journal of cerebral circulation* 2014;45(2):553-62. doi: 10.1161/strokeaha.113.003216 [published Online First: 2014/01/05]

31. Earnshaw SR, McDade C, Chapman AM, et al. Economic impact of using additional diagnostic tests to better select patients with stroke for intravenous thrombolysis in the United Kingdom. *Clinical therapeutics* 2012;34(7):1544-58. doi: 10.1016/j.clinthera.2012.05.004 [published Online First: 2012/06/15]

For peer review only

32. Dirks M, Baeten SA, Dippel DW, et al. Real-life costs and effects of an implementation program to increase thrombolysis in stroke. *Neurology* 2012;79(6):508-14. doi: 10.1212/WNL.0b013e31826356bf [published Online First: 2012/07/13]

33. Barton M, McClean S, Gillespie J, et al. Is it beneficial to increase the provision of thrombolysis?-- a discrete-event simulation model. *QJM : monthly journal of the Association of Physicians* 2012;105(7):665-73. doi: 10.1093/qjmed/hcs036 [published Online First: 2012/03/03]

34. Joshi JK, Ouyang B, Prabhakaran S. Should TIA patients be hospitalized or referred to a same-day clinic?: a decision analysis. *Neurology* 2011;77(24):2082-8. doi: 10.1212/WNL.0b013e31823d763f [published Online First: 2011/11/19]

35. Nguyen-Huynh MN, Johnston SC. Is hospitalization after TIA cost-effective on the basis of treatment with tPA? *Neurology* 2005;65(11):1799-801. doi: 10.1212/01.wnl.0000187067.93321.fa [published Online First: 2005/12/14]

36. Giles MF, Rothwell PM. Substantial underestimation of the need for outpatient services for TIA and minor stroke. *Age and ageing* 2007;36(6):676-80. doi: 10.1093/ageing/afm088 [published Online First: 2007/07/28]

37. Beech P, Greenhalgh J, Thornton M, et al. A survey of the practice of stroke doctors in developing transient ischaemic attack services in the UK. *Journal of evaluation in clinical practice* 2007;13(3):395-9. doi: 10.1111/j.1365-2753.2006.00714.x [published Online First: 2007/05/24]

For peer review only

Figure legends

Figure 1. Example survival curves and daily hazard rates for risk of disabling stroke in high and low risk patients.

High risk patient: male age 70-74, SBP 156mmHg, Speech disturbance without weakness, Duration of symptoms 60+ mins, not diabetic (resulting in ABCD and $ABCD^2$ scores of 5), undiagnosed AF, cholesterol 5.8. Low risk patient: male age 70-74, SBP 115mmHg, Speech disturbance without weakness, Duration of symptoms 0-9 mins, not diabetic (resulting in ABCD and $ABCD^2$ of scores 2), AF on warfarin, cholesterol 6.0.

Figure 2. Outcomes of service provision in terms of clinic appointment utilisation and guideline breaches.

OP = outpatient appointment; TIA = transient ischemic attack; LR = low risk; HR = high risk. Original and modified services (first two bars at each hospital) were those which were actually implemented in each hospital. The remaining weekend services are hypothetical. *Hospital 1 included 16 routine clinics, 5 days per week (original service), 17 in the modified service and 19 in the modified service with weekend working. †Hospital 2 included 12 routine clinics, 3 days per week (original service), and 14 in the modified service (which also included weekend working).

For peer review only

For peer review only - http://bmjopen.bmj.com/site/about/guidelines.xhtml

Tables

Table 1. Patient characteristics as modelled

**Patient characteristics**	
Age (mean±SD) (years)	74 ± 12
Gender (% female)	240 (48.9%)
Systolic blood pressure (mean ± SD) (mmHg)	
TIA mimic	143 ± 26
TIA	147 ± 22
Minor stroke	144 ± 18
Overall mean	145 ± 24
Final Diagnosis (Actual condition)	
High risk TIA	116 (23.6%)
Low risk TIA	46 (9.4%)
TIA mimic	294 (60.0%)
Minor stroke	34 (7.0%)
*Patients referral to hospital via their GP according to diagnosis**	
High risk TIA	84 (72.3%)
Low risk TIA/ TIA mimic	299 (87.9%)
Minor stroke	20 (57.4%)
Overall mean	402 (82.1%)

SD = Standard deviation; TIA = Transient ischemic attack; GP = general practitioner. [*]Expected numbers are rounded to the nearest integer, the apparent anomaly with the addition results from this rounding. High risk patients defined as an ABCD^2 score of ≥4

For peer review only

Table 2. Pattern of outpatient clinics for suspected TIA based on actual service provision at participating hospitals during patient recruitment period.

Hospital and service		Number of routine clinic slots available by day of the week					Total clinics per year	Details of clinic allocation within the model
		Mon	*Tues*	*Wed*	*Thur*	*Fri*		
Hospital 1	Original service*	2	4	4	4	2	724	All patients are assigned to the next available clinic slots in order of referral. Where two referrals are made in one day, high risk patients are given priority.
	Modified service*	4	4	2	3	4	769	Where two referrals are made in one day, high patients are given priority. One slot is reserved at the end of each clinic for high risk referrals. If the next high risk clinic slot is not within 24 hours of referral, an additional slot is made available (up to 1 per day). All other patients are assigned to the next available clinic slots in order of referral.
Hospital 2	Original service†	6	4	2	0	0	624	Patients are assigned to the next available outpatient clinic. Those high risk patients who cannot be seen within 24 hours are admitted (including those presenting at weekends).
	Modified service†	4 or 6‡	0	4	0	4	676	All high risk patients are seen on the ward as outpatients as required (including at weekends). Patients referred before 10am are seen at 5pm on the same day, patients referred after 10am are seen at 10am on the following day. All low risk patients seen at the next available clinic in order of referral.

*Clinics are divided among 4 specialists, each of whom were absent for approximately 7 weeks a year (annual leave). These clinics are assumed not to take place if the specialist is absent.

†Specialists were absent for approximately 7 weeks a year (annual leave). All absent clinicians were replaced by a specialist from another site within the Trust.

‡There is a 50% probability each week that either 4 or 6 clinic slots will be available.

For peer review only - http://bmjopen.bmj.com/site/about/guidelines.xhtml

Table 3. Costs, resource utilisation and outcomes (per year) of modifying TIA service provision in hospital 1.

	**Original service (16 clinic slots per week)**	**Modified service (17 clinic slots per week)**	**Weekend service + 17 clinic slots per week)**	**Weekend service + 15 clinic slots per week)**	**Weekend service + 13 clinic slots per week)**
Days operating per week	5 days	5 days	7 days	7 days	7 days
Total number of patients presenting	491	490	490	491	491
Cost of clinics used & unused	£340,000	£361,000	£366,000	£346,000	£325,000
Disabling strokes post TIA (mean, 99% CI)*	10.6 (10.4-10.8)	10.7 (10.5-10.9)	10.6 (10.4-10.8)	10.8 (10.6-11.0)	10.6 (10.4-10.8)
Number of high risk breaches† (%)	103 (69%)	32 (21%)	6 (4%)	7 (5%)	8 (5%)
Number of low risk breaches‡ (%)	7 (2%)	4 (1%)	3 (1%)	13 (4%)	75 (22%)
Time from referral to clinic appointment for high risk patients in days (median, IQR)	1.15 (0.93-2.88)	0.85 (0.17-0.99)	0.68 (0.16-0.93)	0.70 (0.15-0.93)	0.86 (0.16-0.99)

TIA = transient ischemic attack; CI = confidence intervals; IQR = inter-quartile range. Of the ~490 patients in the model, 340 are considered low risk (294 TIA mimic; 46 low risk TIA) and 150 are considered high risk (116 high risk TIA; 34 minor stroke). *Point estimate and 99% quasi confidence interval reflecting the uncertainty from sampling in the model, not any uncertainty in model parameters. 99% was chosen because of multiple values were compared. †High risk breaches were defined as high risk patients not seen by a specialist within 24 hours of initial clinic referral.[12] ‡ Low risk breaches were defined as low risk patients not seen by a specialist within 7 days of initial clinic referral.

For peer review only

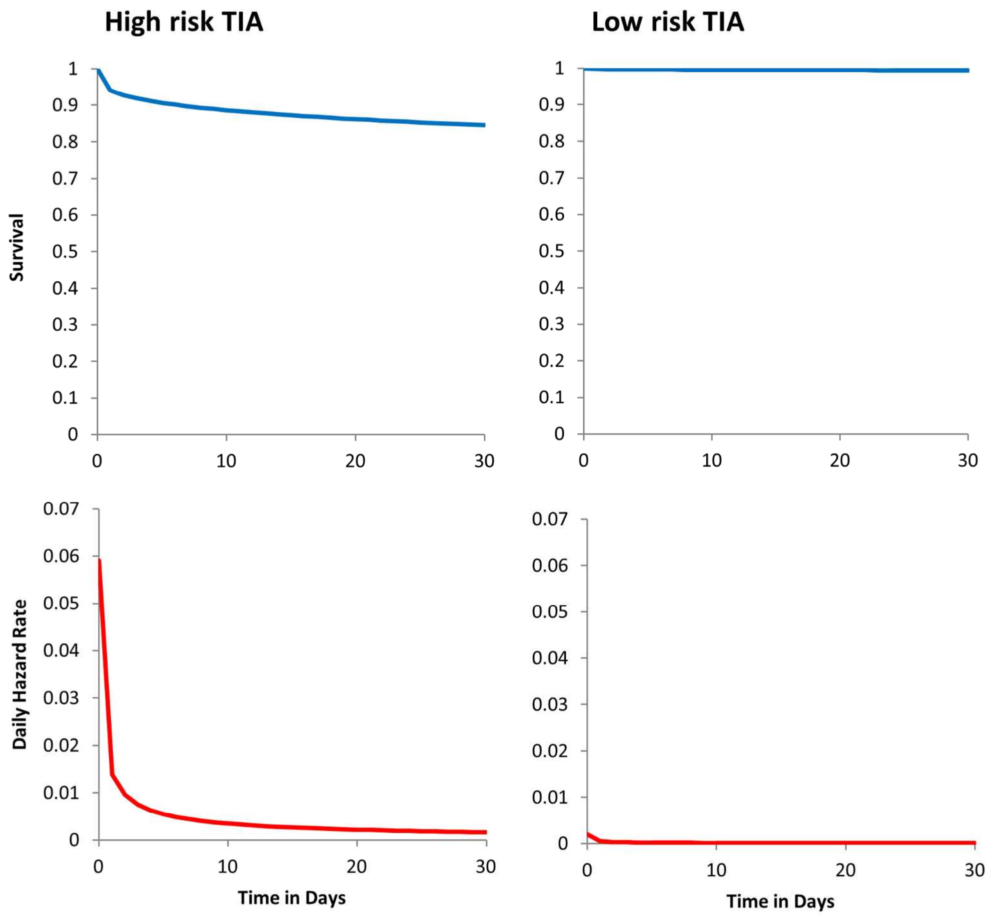

Figure 1. Example survival curves and daily hazard rates for risk of disabling stroke in high and low risk patients.

High risk patient: male age 70-74, SBP 156mmHg, Speech disturbance without weakness, Duration of symptoms 60+ mins, not diabetic (resulting in ABCD and ABCD2 scores of 5), undiagnosed AF, cholesterol 5.8. Low risk patient: male age 70-74, SBP 115mmHg, Speech disturbance without weakness, Duration of symptoms 0-9 mins, not diabetic (resulting in ABCD and ABCD2 of scores 2), AF on warfarin, cholesterol 6.0.

172x160mm (300 x 300 DPI)

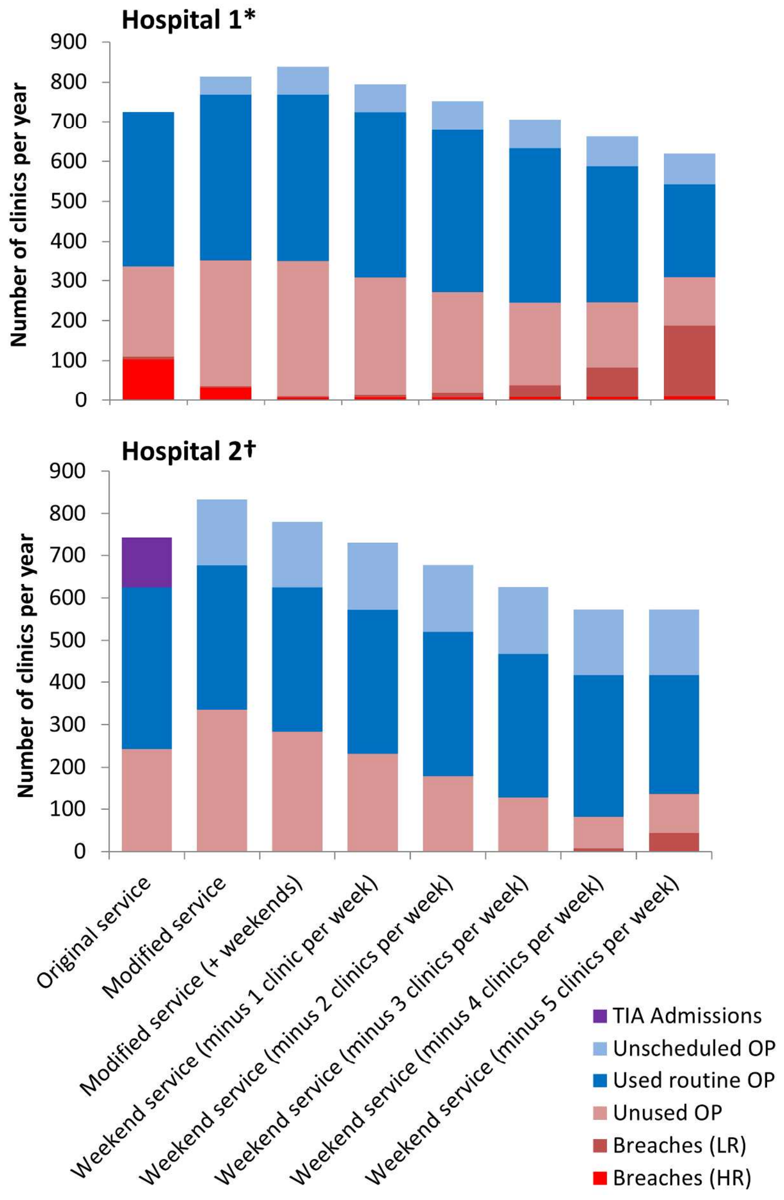

Figure 2. Outcomes of service provision in terms of clinic appointment utilisation and guideline breaches.

OP = outpatient appointment; TIA = transient ischemic attack; LR = low risk; HR = high risk. Original and modified services (first two bars at each hospital) were those which were actually implemented in each hospital. The remaining weekend services are hypothetical. *Hospital 1 included 16 routine clinics, 5 days per week (original service), 17 in the modified service and 19 in the modified service with weekend working. †Hospital 2 included 12 routine clinics, 3 days per week (original service), and 14 in the modified service (which also included weekend working).

172x260mm (300 x 300 DPI)

Online data supplement

For peer review only

Contents

1. **Extended methods**
2. **eFigure 1.** Care pathway for patients presenting with symptoms of transient ischemic attack
3. **eTable 1.** Sources of data included in the model
4. **eTable 2.** Resource unit costs included in the service provision model
5. **eTable 3.** Costs, resource utilisation and outcomes (per year) of modifying TIA service provision in hospital 2.
6. **References**

For peer review only

Extended methods

Setting and service design

The model was designed to assess the costs and clinical consequences arising from patients suspected of suffering an acute TIA and referred to two large urban hospitals in the West Midlands region of the UK. Both hospitals ran a specialist TIA outpatient service catering for approximately 500 patients with suspected stroke and TIA every year. The model parameters were defined by the characteristics of individuals attending these services and recruited to an observational study[1] during 2011 (table 1).

The availability of specialist TIA clinics was modelled on the basis of existing service provision at participating hospital sites during the study period. Patients could either be seen in traditional outpatient clinics, admitted, or seen on the ward on an outpatient basis. The original services were designed within the confines of available clinical staff and a lack of specialist cover at the weekends. Both services subsequently underwent redesign and the impact of these changes was examined by modelling both the original and modified service. The number and distribution of clinics in each service are described in Table 2.

The impact of further adjustments to the modified service at each site was modelled to replicate the following scenarios:

a) The addition of high risk clinic slots for patients presenting on a Saturday and Sunday
b) Including a weekend service but reducing the number of routine weekday clinics, by up to 5 clinic slots per week.

Overview of the model

A discrete event simulation model was programmed in Delphi version 4 (Borland, San Francisco, CA, USA). It was adapted from a previously developed model and further detail regarding the general modelling framework can be found in the original report.[2] Briefly, the model generated a number of possible (virtual) patient histories which began at the onset of TIA (or TIA like) symptoms and followed the patient along the clinical pathway from initial presentation to referral, specialist TIA clinic attendance, treatment and lifetime follow-up for subsequent stroke morbidity and mortality. An essential feature of the model was that patients were sharing limited resources in the form of routine clinics.

For peer review only - http://bmjopen.bmj.com/site/about/guidelines.xhtml

The model estimated the impact of existing service provision, the effect of recent service change and the impact of further alterations to clinic availability on service costs/provision and subsequent stroke events, from a health services perspective.

Clinical pathways in the model

Following onset of an initial event, patients were assumed to contact either their general practitioner (GP) or attend the emergency department (ED) (eFigure 1, online supplemental material). The probability that a patient would choose a specific route was dependent on the type of patient (high/low risk), the time of day, and day of week, estimated using data from the Oxford Vascular Study.[3] Time taken to reach the service depended on the route chosen; where patients directly attended the ED, the time was sampled from a Weibull distribution fitted to data from the Newcastle Stroke Service.[2] Patients attending their GP were assumed to arrive within daytime clinic hours.[3] Following this initial contact with a healthcare professional, patients were referred to a specialist TIA outpatient clinic, seen on the ward (as an outpatient) or admitted as an inpatient. The type of referral was dependant on the specific hospital service provision, clinic availability and the level of risk of the attending patient, defined according to the $ABCD^2$ score.[4] During clinic attendance, it was assumed that the appropriate treatment would be initiated (i.e. blood pressure lowering, cholesterol lowering or antiplatelet therapy in accordance with guidelines) and a small proportion of patients (4.1%) would be deemed suitable for carotid endarterectomy.[5, 6]

Model population

Overall rates of patient presentation were based on initial runs of the model and ensured a realistic distribution of final diagnoses which represented the study sample, previous literature[7] and expert opinion (60% TIA mimics, 33% genuine TIA, and 7% minor stroke). In the absence of robust data for the distribution of new referrals through the week, it was assumed that new cases arrived uniformly.

Due to missing risk data in the sample population, the observed ratio of low risk/high risk TIA patients used as basecase in the model was supplemented in sensitivity analyses by estimates derived from those reported in previous studies[4, 8, 9] and the experience of stroke physicians from centres across the UK [personal communications, D Sims, G Ford and C Roffe]. The range of these estimates tested in this sensitivity analysis for impact on the model results was: 2.5:1 (high:low risk; base-case) to 1:1, 5:1 and 7:1.[4, 8, 9]

For peer review only

Follow-up and risk of repeat events

Patients remained in the model until one year from symptom onset or until they suffered a repeat event (within the same year). Repeat TIA events meant restarting the clinical pathway, subsequent stroke was counted as an outcome and costed accordingly. The risk of a repeat event (TIA or major stroke) was dependent on the initial event (true TIA or mimic), the severity of that event (measured by ABCD^2 score) and other relevant risk factors such as age, presence of atrial fibrillation and medication prescribed.[4, 6, 10-15] Risks were modelled using a Weibull distribution for the time to event which allowed for a substantially increased risk in the short term, followed by a decreasing risk over time.[16] Patients were considered to have a risk of "other cause" death, on the basis of age group and gender, based on national statistics for "all cause" death adjusted for stroke deaths,[17-19] allowing a date of death to be sampled for each individual patient. In the vast majority of cases, the date of other cause death was beyond the follow-up time in the model.

The model was run 100 times for a total simulated time of 12 years in each run: the first year of the run was regarded as a "warm up" period to allow the system to reach a steady state before collecting data. Patients entering during the next 10 years were included in the model results, with the final year being necessary to allow for a one-year follow up time for all included patients. Results for patients entering the model during this last "follow up" period were not included, but such patients were modelled to ensure that the constraints on clinic availability were maintained. Results are presented in terms of annual costs and other outcomes.

Costs and outcomes

The model included costs of GP visits, transport by ambulance to the ED, ED attendance, outpatient clinics, hospital admission for TIA and stroke, surgery and therapy (eTable 1 and 2, online data supplement). Costs included in the model were taken from a combination of NHS reference costs,[20, 21] and drug costs from the British National Formulary.[22] The price year for all costs was 2011-12.

The primary outcome of this study was number of major strokes occurring post TIA. Secondary outcomes included the overall service costs per year and attainment of national targets for TIA service provision. Target attainment was defined as the number of high and low risk 'breaches' which occurred in each service per year: high risk breaches were defined as a high risk patient not seen by a specialist within 24 hours of initial referral.[5, 23] Low risk breaches were defined as low risk patients not seen by a specialist within seven days of referral.[5, 23] Further outcomes examined the median time from referral to specialist appointment, the total number of routine outpatient

For peer review only - http://bmjopen.bmj.com/site/about/guidelines.xhtml

appointments available (used or unused) and any unscheduled outpatient appointments required (where high risk patients were assessed immediately on the ward).

All data are presented as means or medians ± standard deviation (SD), inter-quartile range (IQR) or 99% confidence intervals (CI), chosen because multiple values are compared. Percentages are given for the total population unless otherwise stated.

For peer review only

eFigure 1. Care pathway for patients presenting with symptoms of transient ischemic attack

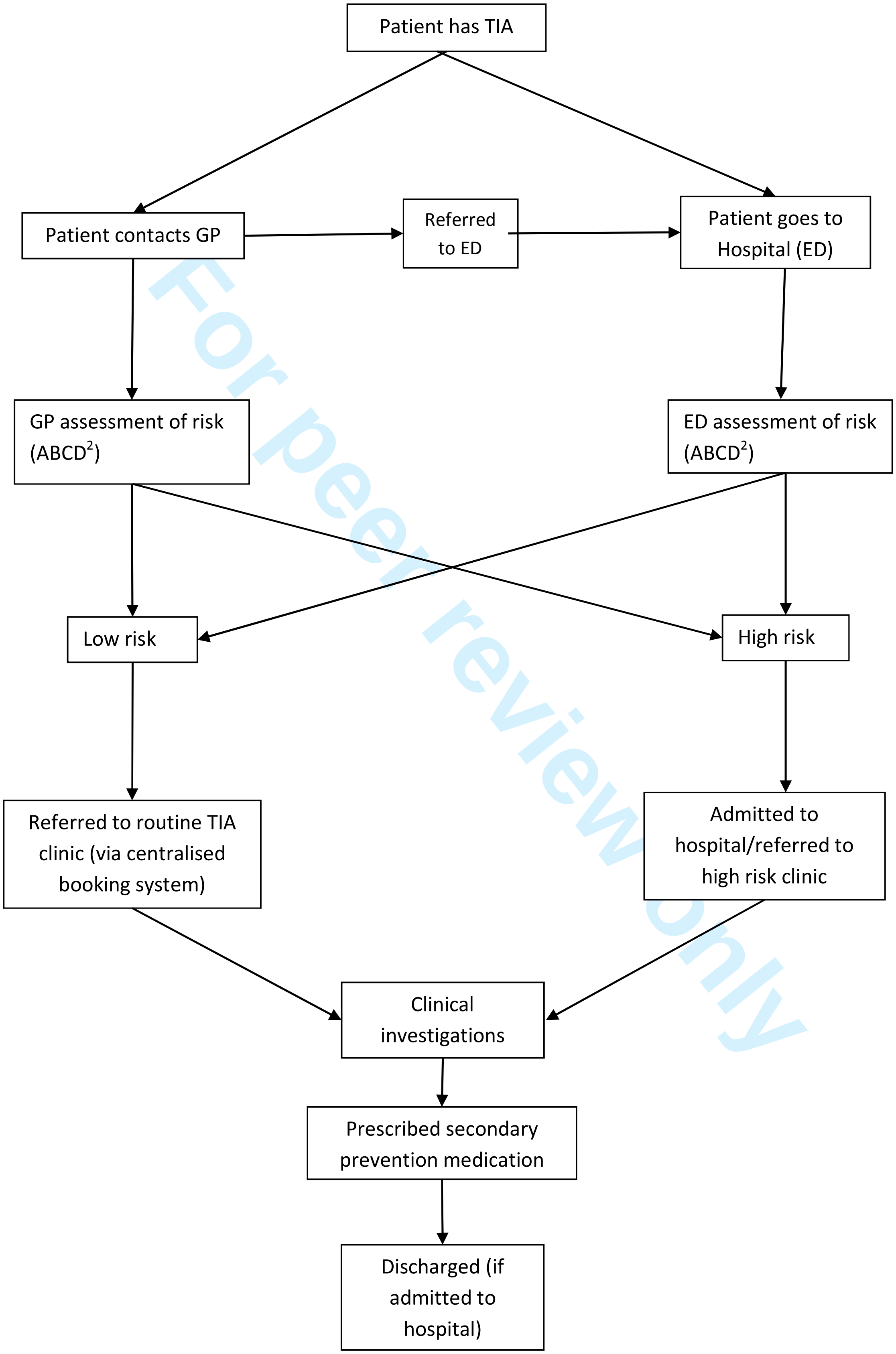

For peer review only

eTable 1. Sources of data included in the model

Model Inputs	Source	References
Schedule of Appointments<br>Casemix (mimic, TIA, minor stroke)	Participating hospital sites, Previous literature and he experience of stroke physicians from centres across the UK (personal communications, D Sims, G Ford and C Roffe)	8, 9
Distribution of patient ages	Data from the OXVASC study adjusted to England and Wales population	24
Patient characteristics (gender, components of $ABCD^2$ score, current medication, AF status, cholesterol level, diabetes conditional on age as appropriate)	Data from the CLAHRC optimisation of the management of stroke and TIA study	1
Patient choice to contact GP or ambulance	OXVASC data	3
Time for patient to reach GP	Assumptions sustained from SDO model	2
Time for patient to reach service using ambulance	Data from Northumbria Ambulance Service	2
"Other cause" death rates	Government Actuary's Department & ONS, Health Statistics Quarterly	17-19
Baseline risk of repeat events	OXVASC data	24
Factors affecting risk of repeat events	Previous literature	10-15, 24

OXVASC = OXford VASCular study; CLAHRC = Collaborations for Leadership in Applied Health Research and Care

For peer review only

eTable 2. Resource unit costs included used in the service provision model.

Item	Cost (£)	Notes	Data source (reference)
Use of an ambulance	230		[20]
General practitioner consultation	33		[21]
Emergency department consultation	143	Category 2 investigation not leading to admitted	[20]
Outpatient appointment	212		[20]
Repeat major stroke	3127		[20]
Assessment for carotid endarterectomy	50	Additional cost of ultrasound only	[20]
Carotid endarterectomy surgery	3923		[20]
Stroke after carotid endarterectomy	3127	As for repeat stroke – small numbers	[20]
Hospital Admission	413		[20]
MRI	92	Applied to 20% of patients at outpatient appointment or hospital admission	[20]
Initiation of antiplatelet therapy*	0.485	300mg loading (from pack of 56 aspirin)	[22]
Antiplatelet mono therapy (per week)*	0.1213	75mg daily from pack of 56 aspirin	[22]
Antiplatelet dual therapy (per week)*	1.0683	Mono plus clopidogrel 75 mg od	[22]
Warfarin (per week)*	0.2150	3mg daily	[22]
Statin (per week)*	0.2925	Simvastatin 40mg daily (from pack of 28)	[22]
Antihypertensive*	0.2025	Bendrofulmethiazide 2.5mg daily	[22]

*Costs taken from the British National Formulary (November 2012) [22]

For peer review only

eTable 3. Costs, resource utilisation and outcomes (per year) of modifying TIA service provision in hospital 2.

	Original service (12 clinic slots per week)	Modified service (14 clinic slots per week)	New service (13 clinic slots per week)	New service (11 clinic slots per week)	New service (9 clinic slots per week)
Days operating per week	7 days	7 days	7 days	7 days	7 days
Total number of patients presenting	490	490	490	490	490
Cost of clinics used & unused	£368,000	£363,000	£353,000	£329,000	£304,000
Disabling strokes post TIA (mean, 99% CI)*	10.7 (10.5-10.9)	10.4 (10.2-10.5)	10.7 (10.5-10.9)	10.6 (10.5-10.8)	10.6 (10.4-10.8)
Number of high risk breaches† (%)	0 (0.0%)	0 (0.0%)	0 (0.0%)	0 (0.0%)	0 (0.0%)
Number of low risk breaches‡ (%)	0 (0.0%)	0 (0.0%)	0 (0.0%)	1 (0.3%)	44 (12.9%)
Time from referral to clinic appointment for high risk patients in days (median, IQR)	0.85 (0.74-0.94)	0.75 (0.08-0.88)	0.75 (0.08-0.88)	0.75 (0.08-0.88)	0.75 (0.08-0.88)

TIA = transient ischemic attack; CI = confidence intervals; IQR = inter-quartile range
Of the ~490 patients in the model, 340 are considered low risk (294 TIA mimic; 46 low risk TIA) and 150 are considered high risk (116 high risk TIA; 34 minor stroke)
*Point estimate and 99% quasi confidence interval reflecting the uncertainty from sampling in the model, not any uncertainty in model parameters. 99% was chosen because of multiple values were compared
†High risk breaches were defined as high risk patients not seen by a specialist within 24 hours of initial clinic referral.
‡ Low risk breaches were defined as low risk patients not seen by a specialist within 7 days of initial clinic referral.

For peer review only

References

1. Sheppard JP, Mellor RM, Bailey SM, et al. Protocol for an observation and implementation study investigating optimisation of the management of stroke and transient ischaemic attack (TIA). *BMJ open* 2012; **2**(3).

2. Mant J, Barton P, McManus RJ, Rothwell PM. What is the Optimum Model of Service Delivery for Transient Ischaemic Attack?: National Co-ordinating Centre for NHS Service Delivery and Organisation R&D (NCCSDO), 2008.

3. Lasserson DS, Chandratheva A, Giles MF, Mant D, Rothwell PM. Influence of general practice opening hours on delay in seeking medical attention after transient ischaemic attack (TIA) and minor stroke: prospective population based study. *BMJ (Clinical research ed)* 2008; **337**: a1569.

4. Rothwell PM, Giles MF, Flossmann E, et al. A simple score (ABCD) to identify individuals at high early risk of stroke after transient ischaemic attack. *Lancet* 2005; **366**(9479): 29-36.

5. National Institute for Health and Clinical Excellence (NICE). Diagnosis and intial management of acute stroke and transient ischemic attack (TIA), 2008.

6. Rothwell PM, Eliasziw M, Gutnikov SA, Warlow CP, Barnett HJ. Endarterectomy for symptomatic carotid stenosis in relation to clinical subgroups and timing of surgery. *Lancet* 2004; **363**(9413): 915-24.

7. Brazzelli M, Shuler K, Quayyum Z, et al. Clinical and imaging services for TIA and minor stroke: results of two surveys of practice across the UK. *BMJ open* 2013; **3**(8).

8. Tsivgoulis G, Stamboulis E, Sharma VK, et al. Multicenter external validation of the ABCD2 score in triaging TIA patients. *Neurology* 2010; **74**(17): 1351-7.

9. Asimos AW, Johnson AM, Rosamond WD, et al. A multicenter evaluation of the ABCD2 score's accuracy for predicting early ischemic stroke in admitted patients with transient ischemic attack. *Annals of emergency medicine* 2010; **55**(2): 201-10.e5.

10. Collaborative meta-analysis of randomised trials of antiplatelet therapy for prevention of death, myocardial infarction, and stroke in high risk patients. *BMJ (Clinical research ed)* 2002; **324**(7329): 71-86.

11. Aguilar MI, Hart R. Oral anticoagulants for preventing stroke in patients with non-valvular atrial fibrillation and no previous history of stroke or transient ischemic attacks. *The Cochrane database of systematic reviews* 2005; (3): Cd001927.

12. Baigent C, Keech A, Kearney PM, et al. Efficacy and safety of cholesterol-lowering treatment: prospective meta-analysis of data from 90,056 participants in 14 randomised trials of statins. *Lancet* 2005; **366**(9493): 1267-78.

13. Ebrahim S, Harwood R. Stroke epidemiology, evidence, and clinical practice. Oxford University Press: Oxford; 1999.

14. MacMahon S. BP and the risks of CV disease. In: Swales J, ed. Textbook of hypertension. Oxford: Blackwell Scientific Publications; 1994.

For peer review only

15. Wolf PA, Abbott RD, Kannel WB. Atrial fibrillation as an independent risk factor for stroke: the Framingham Study. *Stroke; a journal of cerebral circulation* 1991; **22**(8): 983-8.

16. Rothwell PM, Giles MF, Chandratheva A, et al. Effect of urgent treatment of transient ischaemic attack and minor stroke on early recurrent stroke (EXPRESS study): a prospective population-based sequential comparison. *Lancet* 2007; **370**(9596): 1432-42.

17. Government Actuary's Department. Interim life tables. http://www.gad.gov.uk/, 2006.

18. Health Statistics Quarterly. Deaths by age, sex and underlying cause, 2005 registrations. http://www.statistics.gov.uk/statbase/, 2007.

19. Office for National Statistics. Interim Life Tables for England. http://www.ons.gov.uk/ons/publications/index.html, 2011.

20. Department of Health. 2011–12 Reference Costs. 2013. https://www.gov.uk/government/publications/2011-12-reference-costspublication (accessed June 2013).

21. Curtis L. Unit Costs in Health and Social Care 2011. 2012. http://www.pssru.ac.uk/project-pages/unit-costs/2011/index.php. (accessed November 2012).

22. British National Formulary (BNF). Pharmaceutical Press. 2012. https://www.bnf.org/ (accessed November 2012).

23. Intercollegiate Stroke Working Party. National clinical guideline for stroke. London: Royal College of Physicians, 2012.

24. Rothwell PM, Coull AJ, Giles MF, et al. Change in stroke incidence, mortality, case-fatality, severity, and risk factors in Oxfordshire, UK from 1981 to 2004 (Oxford Vascular Study). *Lancet* 2004; **363**(9425): 1925-33.

For peer review only

CHEERS Checklist
Items to include when reporting economic evaluations of health interventions

The **ISPOR CHEERS Task Force Report**, *Consolidated Health Economic Evaluation Reporting Standards (CHEERS)—Explanation and Elaboration: A Report of the ISPOR Health Economic Evaluations Publication Guidelines Good Reporting Practices Task Force*, provides examples and further discussion of the 24-item CHEERS Checklist and the CHEERS Statement. It may be accessed via the *Value in Health* or via the ISPOR Health Economic Evaluation Publication Guidelines – CHEERS: Good Reporting Practices webpage: http://www.ispor.org/TaskForces/EconomicPubGuidelines.asp

Section/item	Item No	Recommendation	Reported on page No/ line No
**Title and abstract**			
Title	1	Identify the study as an economic evaluation or use more specific terms such as “cost-effectiveness analysis”, and describe the interventions compared.	
Abstract	2	Provide a structured summary of objectives, perspective, setting, methods (including study design and inputs), results (including base case and uncertainty analyses), and conclusions.	
**Introduction**			
Background and objectives	3	Provide an explicit statement of the broader context for the study. Present the study question and its relevance for health policy or practice decisions.	
**Methods**			
Target population and subgroups	4	Describe characteristics of the base case population and subgroups analysed, including why they were chosen.	
Setting and location	5	State relevant aspects of the system(s) in which the decision(s) need(s) to be made.	
Study perspective	6	Describe the perspective of the study and relate this to the costs being evaluated.	
Comparators	7	Describe the interventions or strategies being compared and state why they were chosen.	
Time horizon	8	State the time horizon(s) over which costs and consequences are being evaluated and say why appropriate.	
Discount rate	9	Report the choice of discount rate(s) used for costs and outcomes and say why appropriate.	
Choice of health outcomes	10	Describe what outcomes were used as the measure(s) of benefit in the evaluation and their relevance for the type of analysis performed.	
Measurement of effectiveness	11a	*Single study-based estimates:* Describe fully the design features of the single effectiveness study and why the single study was a sufficient source of clinical effectiveness data.	

For peer review only

	11b	*Synthesis-based estimates:* Describe fully the methods used for identification of included studies and synthesis of clinical effectiveness data.	
Measurement and valuation of preference based outcomes	12	If applicable, describe the population and methods used to elicit preferences for outcomes.	
Estimating resources and costs	13a	*Single study-based economic evaluation:* Describe approaches used to estimate resource use associated with the alternative interventions. Describe primary or secondary research methods for valuing each resource item in terms of its unit cost. Describe any adjustments made to approximate to opportunity costs.	
	13b	*Model-based economic evaluation:* Describe approaches and data sources used to estimate resource use associated with model health states. Describe primary or secondary research methods for valuing each resource item in terms of its unit cost. Describe any adjustments made to approximate to opportunity costs.	
Currency, price date, and conversion	14	Report the dates of the estimated resource quantities and unit costs. Describe methods for adjusting estimated unit costs to the year of reported costs if necessary. Describe methods for converting costs into a common currency base and the exchange rate.	
Choice of model	15	Describe and give reasons for the specific type of decision-analytical model used. Providing a figure to show model structure is strongly recommended.	
Assumptions	16	Describe all structural or other assumptions underpinning the decision-analytical model.	
Analytical methods	17	Describe all analytical methods supporting the evaluation. This could include methods for dealing with skewed, missing, or censored data; extrapolation methods; methods for pooling data; approaches to validate or make adjustments (such as half cycle corrections) to a model; and methods for handling population heterogeneity and uncertainty.	
**Results**			
Study parameters	18	Report the values, ranges, references, and, if used, probability distributions for all parameters. Report reasons or sources for distributions used to represent uncertainty where appropriate. Providing a table to show the input values is strongly recommended.	
Incremental costs and outcomes	19	For each intervention, report mean values for the main categories of estimated costs and outcomes of interest, as well as mean differences between the comparator groups. If applicable, report incremental cost-effectiveness ratios.	
Characterising uncertainty	20a	*Single study-based economic evaluation:* Describe the effects of sampling uncertainty for the estimated incremental cost and incremental effectiveness parameters, together with the impact	

For peer review only

		of methodological assumptions (such as discount rate, study perspective).	
	20b	*Model-based economic evaluation:* Describe the effects on the results of uncertainty for all input parameters, and uncertainty related to the structure of the model and assumptions.	
Characterising heterogeneity	21	If applicable, report differences in costs, outcomes, or cost-effectiveness that can be explained by variations between subgroups of patients with different baseline characteristics or other observed variability in effects that are not reducible by more information.	
**Discussion**			
Study findings, limitations, generalisability, and current knowledge	22	Summarise key study findings and describe how they support the conclusions reached. Discuss limitations and the generalisability of the findings and how the findings fit with current knowledge.	
**Other**			
Source of funding	23	Describe how the study was funded and the role of the funder in the identification, design, conduct, and reporting of the analysis. Describe other non-monetary sources of support.	
Conflicts of interest	24	Describe any potential for conflict of interest of study contributors in accordance with journal policy. In the absence of a journal policy, we recommend authors comply with International Committee of Medical Journal Editors recommendations.	

For consistency, the CHEERS Statement checklist format is based on the format of the CONSORT statement checklist

The **ISPOR CHEERS Task Force Report** provides examples and further discussion of the 24-item CHEERS Checklist and the CHEERS Statement. It may be accessed via the *Value in Health* link or via the ISPOR Health Economic Evaluation Publication Guidelines – CHEERS: Good Reporting Practices webpage: http://www.ispor.org/TaskForces/EconomicPubGuidelines.asp

The citation for the CHEERS Task Force Report is:
Husereau D, Drummond M, Petrou S, et al. Consolidated health economic evaluation reporting standards (CHEERS)—Explanation and elaboration: A report of the ISPOR health economic evaluations publication guidelines good reporting practices task force. Value Health 2013;16:231-50.

For peer review only

When has service provision for transient ischemic attack improved enough? A discrete event simulation economic modelling study

Journal:	*BMJ Open*
Manuscript ID	bmjopen-2017-018189.R1
Article Type:	Research
Date Submitted by the Author:	29-Aug-2017
Complete List of Authors:	Barton, Pelham; University of Birmingham, Health Economics Unit<br>Sheppard, James; University of Oxford, Nuffield Department of Primary Care Health Sciences<br>Penaloza, Cristina; University of Birmingham, School of Health and Population Sciences<br>Jowett, Sue; University of Birmingham, Health Economics Unit<br>Ford, Gary; Oxford University Hospitals NHS Foundation Trust, Oxford Academic Health Science Network<br>Lasserson, Daniel; Oxford University Hospitals NHS Foundation Trust, NIHR Oxford Biomedical Research Centre<br>Mant, Jonathan; University of Cambridge, General Practice and Primary Care Research Unit<br>Mellor, Ruth; NHS Lanarkshire, Department of Public Health<br>Quinn, Tom; St George's Univesity of London & Kingston University, Joint Faculty of Health, Social Care & Education<br>Rothwell, Peter; Stroke Prevention Research Unit, Nuffield Department of Clinical Neurosciences<br>Sandler, David; Heart of England NHS Foundation Trust,<br>Sims, Don; University Hospitals Birmingham NHS Foundation Trust, Stroke Medicine<br>McManus, Richard; University of Oxford, Dept of Primary Care Health Sciences
**Primary Subject Heading**:	Health economics
Secondary Subject Heading:	Cardiovascular medicine, Neurology
Keywords:	Economic modelling, Stroke < NEUROLOGY, Secondary Care, Secondary prevention, Health services

SCHOLARONE™
Manuscripts

For peer review only - http://bmjopen.bmj.com/site/about/guidelines.xhtml

When has service provision for transient ischemic attack improved enough? A discrete event simulation economic modelling study

Modelling changes to service provision for transient ischemic attack

Barton, P.,[1] Sheppard, J.P.,[2] Penaloza-Ramos, M.C.,[1] Jowett, S.,[1] Ford, G.A.,[3] Lasserson, D.,[4] Mant, J.,[5] Mellor, R.M.,[6] Quinn, T.,[7] Rothwell, P.M.,[8] Sandler, D.,[9] Sims, D.,[10] McManus, R.J.[2] on behalf of the BBC CLAHRC investigators

[1]Health Economics Unit, University of Birmingham, Edgbaston, Birmingham, UK

[2]Nuffield Department of Primary Care Health Sciences, NIHR School for Primary Care Research, University of Oxford, Oxford, Oxfordshire, UK

[3]Oxford Academic Health Science Network, Oxford, Oxfordshire, UK

[4]Nuffield Department of Medicine, University of Oxford, Oxford, Oxfordshire, UK

[5]Primary Care Unit, University of Cambridge, Cambridge, Cambridgeshire, UK,

[6]Department of Public Health, NHS Lanarkshire, Kirklands, Fallside Road, Bothwell, UK

[7]Faculty of Health, Social Care and Education, St George's, University of London & Kingston University, London, UK

[8]Nuffield Department of Clinical Neurosciences, University of Oxford, Oxfordshire, UK.

[9]Heart of England NHS Foundation Trust, Birmingham, West Midlands, UK

[10]University Hospitals Birmingham NHS Foundation Trust, Birmingham, West Midlands, UK

Corresponding Author: James Sheppard

Address: Nuffield Department of Primary Care Health Sciences, University of Oxford, Radcliffe Primary Care, Radcliffe Observatory Quarter, Woodstock Road, Oxford, OX2 6GG

Email: james.sheppard@phc.ox.ac.uk

For peer review only

Telephone: +44 (0)1865 617192

Fax: +44 (0)1865 289287

Word count: 3,302 (not including abstract, tables, figures or references)

Key words: Economic modelling, stroke, secondary care, secondary prevention, health services

For peer review only

Abstract

Objectives: The aim of this study was to examine the impact of transient ischaemic attack service modification in two hospitals on costs and clinical outcomes.

Design: Discrete event simulation model using data from routine electronic health records from 2011.

Participants: Patients with suspected TIA were followed from symptom onset to presentation, referral to specialist clinics, treatment and subsequent stroke.

Interventions: Included existing versus previous (less same day clinics) and hypothetical service reconfiguration (7-day service with less availability of clinics per day).

Outcome measures: The primary outcome of the model was the prevalence of major stroke after TIA. Secondary outcomes included service costs (including those of treating subsequent stroke) and time to treatment and attainment of national targets for service provision (proportion of high risk patients [according to $ABCD^2$ score] seen within 24 hours).

Results: The estimated costs of previous service provision for 490 patients (aged 74±12 years, 48.9% female and 23.6% high risk) per year at each site were £340,000 and £368,000 respectively. This resulted in 31% of high risk patients seen within 24 hours of referral (47/150) with a median time from referral to clinic attendance/treatment of 1.15 days (IQR 0.93-2.88). The costs associated with the existing and hypothetical services decreased by £5,000 at one site and increased £21,000 at the other site. Target attainment was improved to 79% (118/150). However, the median time to clinic attendance was only reduced to 0.85 days (IQR 0.17-0.99) and thus no appreciable impact on the modelled incidence of major stroke was observed (10.7 per year, 99%CI 10.5-10.9 [previous service] vs. 10.6 per year, 99%CI 10.4-10.8 [existing service]).

Conclusions: Reconfiguration of services for TIA is effective at increasing target attainment, but in services which are already working efficiently (treating patients within 1-2 days), it has little estimated impact on clinical outcomes and increased investment may not be worthwhile.

For peer review only - http://bmjopen.bmj.com/site/about/guidelines.xhtml

Word count: 306

For peer review only

For peer review only - http://bmjopen.bmj.com/site/about/guidelines.xhtml

Modelling the impact of further hypothetical service reconfiguration, including a seven day service, suggested that it was possible to reduce service costs and the number of unused clinic appointments and improve guideline target attainment, but this had little impact on the incidence of major stroke post TIA or post-stroke deaths (table 3 and eTable 3). Figure 2 shows the trade-off between routine appointment provision, unscheduled appointments and high/low risk guideline breaches. Assuming unused appointments (pink), unscheduled appointments (in light blue), admissions (purple) and breaches (red [high risk breaches] and light red [low risk breaches]) are preferably avoidable outcomes of service provision, the optimal hypothetical service in hospital 1 involved a weekend service with approximately 17-18 routine clinic appointments per week. In hospital 2, where more unscheduled appointments were incurred, the optimal hypothetical service involved a weekend service with 10-11 routine clinic slots distributed across the week on Mondays, Wednesdays and Fridays (with high risk patients being seen in unscheduled appointments on the ward on any day of the week if necessary).

Discussion

The aim of this study was to model the impact of TIA service reconfiguration in two large urban hospitals and establish the impact on clinical outcomes (major strokes), costs, and clinic usage. Reconfiguration of services was found to be effective and appropriate for reducing resource (clinic slot) waste, and improving guideline target attainment with variable effects on costs. However, in hospitals where services, despite poor target attainment, had minimal patient delays, such as those studied here, significant reconfiguration had little impact on modelled clinical outcomes such as major stroke post TIA. These findings suggest that clinicians and service coordinators should be cautious before initiating significant reconfiguration of services which are already seeing high risk patients within 1-2 days, unless there is an obvious need to free up resources which could be used for another purpose (e.g. managing other types of patients). Whilst national targets for care are

For peer review only

important, consideration should be given to revising them to maximise the benefits of attainment whilst not incentivising small changes that might increase costs for no measurable clinical benefit.

Strengths and limitations

The present study used local hospital data to describe the characteristics of the presenting population and thus the results are likely to be representative of the hospitals studied, but not necessarily generalizable to other centres in the UK and across the world. Despite this, guideline target attainment in hospital 1 (original service configuration) was similar to that reported nationally (31% vs. 37% nationally)[13] which suggests that this hospital was more likely representative of centres across the UK.

Modelling allows estimation of service ‘unknowns’, such as the number of unscheduled clinic appointments required to meet guideline targets for high risk patients. This requires pre-programming with hypothetical patients and specific rules; therefore simplification of certain service intricacies is necessary. For example, the process by which high risk patients were allocated unscheduled clinic appointments was formalised such that a patient referred by the ED physician was assumed to have been seen by a stroke specialist within two hours. However, patients referred by their GP were assumed to be seen by the stroke specialist on the ward at 10am the following morning or 5pm the same day, whichever was later (but within 24 hours of initial referral). In reality, services are often adapted in response to a specific set of circumstances such as clinic room availability and thus the services modelled here, and the patients attending these services, can best be thought of as stylised versions of the actual services available and patients attending the hospitals in question.

The risk of recurrent stroke attributed to hypothetical patients in the model was based on an estimated $ABCD^2$ score.[10] The accuracy of this score for predicting recurrent stroke has been called

For peer review only - http://bmjopen.bmj.com/site/about/guidelines.xhtml

into question with a recent systematic review showing it to have poor discrimination between those at low and high risk of early (7 day) stroke.[30] However, this was the score used and recommended in practice at the time these services were modelled and was used for all service configurations examined, so our relative comparisons are likely to remain valid, even if the absolute numbers may be subject to change.

The costs modelled here were taken from standard NHS reference costs[27 28] and included investigations, treatment and the cost of staff attending patients during each appointment/admission. Costs relate to standard weekday services and increased rates for weekend services were unavailable for this analysis. It is therefore likely that the costs of providing a weekend service may have been underestimated in the present analyses.

Findings in the context of previous literature and implications for practice

Economic modelling has previously been used for determining the impact of service reconfiguration in acute stroke[31-34] but studies examining optimal strategies for TIA service provision are less common. Those that exist, focus on whether or not patients should be admitted or be seen as an outpatient.[16 35 36] It is generally accepted that patient admission for TIA is inefficient and not cost-effective,[16 37-39] but there are a paucity of objective data comparing different configurations of outpatient service in the same setting, limited to questionnaire surveys of stroke physicians[14 40] and the Sentinel Stroke National Audit Programme (SSNAP) in the UK.[13] A recent review identified six models of care designed to avoid patient admission for TIA. These ranged from patient assessment and triage in the ED, a 24-hour nurse-led telephone advice service and the primary care triage system studied here (+/- electronic decision support).[41] The appropriate model of care will depend on the local healthcare system, but consideration of how such a model is implemented in terms of service configuration is warranted, regardless of the system used.

For peer review only

The primary finding of this study was that whilst service reconfiguration can significantly improve guideline target attainment and potentially reduce service costs, such changes have little impact on the estimated prevalence of subsequent stroke, when services are already working efficiently. In the present study, even the largest service reconfiguration (introduction of a 7 day service) only reduced the median time from patient referral to clinic attendance and treatment by 0.3 days (8 hours). This enabled a large number of patients to be seen within 24 hours, improving guideline target attainment but had little impact on stroke risk. The recommendations for a 'see and treat within 24 hours' target for high risk patients were originally based on data from the EXPRESS study,[9] which showed that those patients seen and treated within 24 hours of initial referral had significantly better outcomes than those patients who were not. However, in the original service to which this optimal strategy was compared, the average time from referral to clinic appointment was 3 days (IQR 2-5 days) and time to first treatment was 20 days (IQR 8-53). The improvement in time to initiation of treatment was therefore 20 days, compared to an improvement of 8 hours seen in the present study, which explains the very different results in terms of clinical outcome.

Recent results from SSNAP show that 45% of high risk outpatients and 60% of high risk inpatients are seen and treated within 24 hours of referral, suggesting there is plenty of room for improvement. However, with a median time to first clinic appointment (and treatment) for all patients of just 2 days, the present analyses would suggest further improvements in guideline target attainment may have little impact on major stroke following TIA.

The modified services examined and proposed here may require the capacity to accommodate unscheduled clinic appointments, which is likely to have an opportunity cost affecting the quality of other services running concurrently in the hospital and may result in increased costs with little tangible benefit. In particular, the ability to accommodate such appointments depends on the time of day at which the patient presents, the availability of specialist stroke physicians and scanning

For peer review only - http://bmjopen.bmj.com/site/about/guidelines.xhtml

equipment required to make an accurate diagnosis, as well as the needs of competing services within the hospital (e.g. acute stroke services).

Conclusions

Reconfiguration of specialist services for TIA can be effective and appropriate for reducing costs, reducing the number of unused routine clinic appointments and increasing clinical guideline target attainment. However, where services are already working near optimal, such modification has little impact on clinical outcomes such as subsequent stroke. Clinicians and service coordinators should be cautious before initiating significant reconfiguration of services which are already seeing high risk patients within 1-2 days, although this might still be appropriate if resources within the hospital are freed up and could be used for an alternate purpose.

For peer review only

Declarations

Ethics approval and consent to participate

Approval for this project was obtained from the National Research Ethics Service (NRES) Committee, London – Queen Square (reference; 09/H0716/71).

Data sharing

The datasets analysed during the current study are available from the corresponding author on reasonable request.

Competing interests

The authors declare that they have no competing interests

Funding

This work was supported by the National Institute for Health Research (NIHR) as part of the Collaborations for Leadership in Applied Health Research and Care (CLAHRC) programme for Birmingham and Black Country. JPS held a Medical Research Council (MRC grant number MR/K022032/1) Strategic Skills Postdoctoral Fellowship and is now funded by the NIHR Oxford CLARHC. RJMcM holds an NIHR Professorship.

Authors' contributions

RJMcM, JM, SJ and PB had the original idea and gained the funding for the project. PB undertook the economic analyses. JPS and RMM were responsible for the data collection and JPS wrote the first draft of the manuscript with RJMcM and PB. PB, JPS, MCP, SJ, GAF, DL, JM, RMM, TQ, PMR, DS, DSi and RJM contributed to protocol development, refined the manuscript and approved the final version. RJMcM is the guarantor.

For peer review only

Acknowledgements

We would like to thank Amun Boyal, Kelly Rosborough and the staff at both participating hospitals for their assistance with recruitment and data collection. Thanks to Sheila Bailey and Anita Martin for their administrative support and Primary Care Clinical Research and Trials Unit for building and maintaining the study database.

Birmingham and Black Country Collaborations for Leadership in Applied Health Research and Care investigators include:

Peter Carr, Heart of England NHS Foundation Trust

Sheila Greenfield, Primary Care Clinical Sciences, University of Birmingham

Brin Helliwell, Lay member of Steering Group

Christina Nand, Lay member of Steering Group

Norman Phillips, Lay member of Steering Group

Rob Scott, Birmingham and Midland Eye Centre

Satinder Singh, Primary Care Clinical Sciences, University of Birmingham

Matthew Ward, West Midlands Ambulance Service NHS Trust

References

1. Cancelli I, Janes F, Gigli GL, et al. Incidence of transient ischemic attack and early stroke risk: validation of the ABCD2 score in an Italian population-based study. *Stroke; a journal of cerebral circulation* 2011;42(10):2751-7. doi: 10.1161/strokeaha.110.612705 [published Online First: 2011/08/13]

2. Correia M, Silva MR, Magalhaes R, et al. Transient ischemic attacks in rural and urban northern Portugal: incidence and short-term prognosis. *Stroke; a journal of cerebral circulation* 2006;37(1):50-5. doi: 10.1161/01.STR.0000195209.26543.8f [published Online First: 2005/12/03]

For peer review only - http://bmjopen.bmj.com/site/about/guidelines.xhtml

3. Feigin VL, Shishkin SV, Tzirkin GM, et al. A population-based study of transient ischemic attack incidence in Novosibirsk, Russia, 1987-1988 and 1996-1997. *Stroke; a journal of cerebral circulation* 2000;31(1):9-13. [published Online First: 2000/01/08]

4. Fonseca PG, Weiss PA, Harger R, et al. Transient ischemic attack incidence in joinville, Brazil, 2010: a population-based study. *Stroke; a journal of cerebral circulation* 2012;43(4):1159-62. doi: 10.1161/strokeaha.111.645101 [published Online First: 2011/12/31]

5. Kleindorfer D, Panagos P, Pancioli A, et al. Incidence and short-term prognosis of transient ischemic attack in a population-based study. *Stroke; a journal of cerebral circulation* 2005;36(4):720-3. doi: 10.1161/01.STR.0000158917.59233.b7 [published Online First: 2005/02/26]

6. Rothwell PM, Coull AJ, Giles MF, et al. Change in stroke incidence, mortality, case-fatality, severity, and risk factors in Oxfordshire, UK from 1981 to 2004 (Oxford Vascular Study). *Lancet* 2004;363(9425):1925-33. doi: 10.1016/s0140-6736(04)16405-2 [published Online First: 2004/06/15]

7. Coull AJ, Lovett JK, Rothwell PM. Population based study of early risk of stroke after transient ischaemic attack or minor stroke: implications for public education and organisation of services. *BMJ (Clinical research ed)* 2004;328(7435):326. doi: 10.1136/bmj.37991.635266.44 [published Online First: 2004/01/28]

8. Lovett JK, Dennis MS, Sandercock PA, et al. Very early risk of stroke after a first transient ischemic attack. *Stroke; a journal of cerebral circulation* 2003;34(8):e138-40. doi: 10.1161/01.str.0000080935.01264.91 [published Online First: 2003/07/12]

9. Rothwell PM, Giles MF, Chandratheva A, et al. Effect of urgent treatment of transient ischaemic attack and minor stroke on early recurrent stroke (EXPRESS study): a prospective population-

For peer review only

based sequential comparison. *Lancet* 2007;370(9596):1432-42. doi: 10.1016/s0140-6736(07)61448-2 [published Online First: 2007/10/12]

10. Rothwell PM, Giles MF, Flossmann E, et al. A simple score (ABCD) to identify individuals at high early risk of stroke after transient ischaemic attack. *Lancet* 2005;366(9479):29-36. doi: 10.1016/s0140-6736(05)66702-5 [published Online First: 2005/07/05]

11. Intercollegiate Stroke Working Party. National clinical guideline for stroke. London: Royal College of Physicians, 2012.

12. National Institute for Health and Clinical Excellence (NICE). Diagnosis and intial management of acute stroke and transient ischemic attack (TIA). NICE clinical guideline, 2008.

13. Royal College of Physicians. Sentinel Stroke National Audit Programme (SSNAP) Acute Organisational Audit Report 2014, 2014.

14. Brazzelli M, Shuler K, Quayyum Z, et al. Clinical and imaging services for TIA and minor stroke: results of two surveys of practice across the UK. *BMJ open* 2013;3(8) doi: 10.1136/bmjopen-2013-003359 [published Online First: 2013/08/10]

15. Sheppard JP, Mellor RM, Bailey SM, et al. Protocol for an observation and implementation study investigating optimisation of the management of stroke and transient ischaemic attack (TIA). *BMJ open* 2012;2(3) doi: 10.1136/bmjopen-2012-001430 [published Online First: 2012/06/27]

16. Mant J, Barton P, McManus RJ, et al. What is the Optimum Model of Service Delivery for Transient Ischaemic Attack?: National Co-ordinating Centre for NHS Service Delivery and Organisation R&D (NCCSDO), 2008.

17. Lasserson DS, Chandratheva A, Giles MF, et al. Influence of general practice opening hours on delay in seeking medical attention after transient ischaemic attack (TIA) and minor stroke:

For peer review only

prospective population based study. *BMJ (Clinical research ed)* 2008;337:a1569. doi: 10.1136/bmj.a1569 [published Online First: 2008/09/20]

18. Rothwell PM, Eliasziw M, Gutnikov SA, et al. Endarterectomy for symptomatic carotid stenosis in relation to clinical subgroups and timing of surgery. *Lancet* 2004;363(9413):915-24. doi: 10.1016/s0140-6736(04)15785-1 [published Online First: 2004/03/27]

19. Tsivgoulis G, Stamboulis E, Sharma VK, et al. Multicenter external validation of the ABCD2 score in triaging TIA patients. *Neurology* 2010;74(17):1351-7. doi: 10.1212/WNL.0b013e3181dad63e [published Online First: 2010/04/28]

20. Asimos AW, Johnson AM, Rosamond WD, et al. A multicenter evaluation of the ABCD2 score's accuracy for predicting early ischemic stroke in admitted patients with transient ischemic attack. *Annals of emergency medicine* 2010;55(2):201-10.e5. doi: 10.1016/j.annemergmed.2009.05.002 [published Online First: 2009/06/27]

21. Collaborative meta-analysis of randomised trials of antiplatelet therapy for prevention of death, myocardial infarction, and stroke in high risk patients. *BMJ (Clinical research ed)* 2002;324(7329):71-86. [published Online First: 2002/01/12]

22. Aguilar MI, Hart R. Oral anticoagulants for preventing stroke in patients with non-valvular atrial fibrillation and no previous history of stroke or transient ischemic attacks. *The Cochrane database of systematic reviews* 2005(3):Cd001927. doi: 10.1002/14651858.CD001927.pub2 [published Online First: 2005/07/22]

23. Baigent C, Keech A, Kearney PM, et al. Efficacy and safety of cholesterol-lowering treatment: prospective meta-analysis of data from 90,056 participants in 14 randomised trials of statins. *Lancet* 2005;366(9493):1267-78. doi: 10.1016/s0140-6736(05)67394-1 [published Online First: 2005/10/11]

For peer review only

24. Ebrahim S, Harwood R. Stroke epidemiology, evidence, and clinical practice. Oxford University Press: Oxford 1999.

25. MacMahon S. BP and the risks of CV disease. In: Swales J, ed. Textbook of hypertension. Oxford: Blackwell Scientific Publications 1994.

26. Wolf PA, Abbott RD, Kannel WB. Atrial fibrillation as an independent risk factor for stroke: the Framingham Study. *Stroke; a journal of cerebral circulation* 1991;22(8):983-8. [published Online First: 1991/08/01]

27. Department of Health. 2011–12 Reference Costs 2013 [Available from: https://www.gov.uk/government/publications/2011-12-reference-costspublication accessed June 2013.

28. Curtis L. Unit Costs in Health and Social Care 2011: Personal Social Services Research Unit (PSSRU); 2012 [Available from: http://www.pssru.ac.uk/project-pages/unit-costs/2011/index.php. accessed November 2012.

29. British National Formulary (BNF). Pharmaceutical Press 2012 [Available from: https://www.bnf.org/ accessed November 2012.

30. Wardlaw JM, Brazzelli M, Chappell FM, et al. ABCD2 score and secondary stroke prevention: Meta-analysis and effect per 1,000 patients triaged. *Neurology* 2015;85(4):373-80. doi: 10.1212/wnl.0000000000001780

31. Penaloza-Ramos MC, Sheppard JP, Jowett S, et al. Cost-effectiveness of optimizing acute stroke care services for thrombolysis. *Stroke; a journal of cerebral circulation* 2014;45(2):553-62. doi: 10.1161/strokeaha.113.003216 [published Online First: 2014/01/05]

32. Earnshaw SR, McDade C, Chapman AM, et al. Economic impact of using additional diagnostic tests to better select patients with stroke for intravenous thrombolysis in the United Kingdom.

For peer review only - http://bmjopen.bmj.com/site/about/guidelines.xhtml

Clinical therapeutics 2012;34(7):1544-58. doi: 10.1016/j.clinthera.2012.05.004 [published Online First: 2012/06/15]

33. Dirks M, Baeten SA, Dippel DW, et al. Real-life costs and effects of an implementation program to increase thrombolysis in stroke. *Neurology* 2012;79(6):508-14. doi: 10.1212/WNL.0b013e31826356bf [published Online First: 2012/07/13]

34. Barton M, McClean S, Gillespie J, et al. Is it beneficial to increase the provision of thrombolysis?-- a discrete-event simulation model. *QJM : monthly journal of the Association of Physicians* 2012;105(7):665-73. doi: 10.1093/qjmed/hcs036 [published Online First: 2012/03/03]

35. Joshi JK, Ouyang B, Prabhakaran S. Should TIA patients be hospitalized or referred to a same-day clinic?: a decision analysis. *Neurology* 2011;77(24):2082-8. doi: 10.1212/WNL.0b013e31823d763f [published Online First: 2011/11/19]

36. Nguyen-Huynh MN, Johnston SC. Is hospitalization after TIA cost-effective on the basis of treatment with tPA? *Neurology* 2005;65(11):1799-801. doi: 10.1212/01.wnl.0000187067.93321.fa [published Online First: 2005/12/14]

37. Buisman LR, Tan SS, Nederkoorn PJ, et al. Hospital costs of ischemic stroke and TIA in the Netherlands. *Neurology* 2015;84(22):2208-15. doi: 10.1212/wnl.0000000000001635 [published Online First: 2015/05/03]

38. Ranta A, Lichtman JH. Health economics of cerebrovascular disease: Can we do better? *Neurology* 2015;84(22):2204-5. doi: 10.1212/wnl.0000000000001651 [published Online First: 2015/05/03]

For peer review only

39. Sanders LM, Cadilhac DA, Srikanth VK, et al. Is nonadmission-based care for TIA patients cost-effective?: A microcosting study. *Neurology: Clinical Practice* 2015;5(1):58-66. doi: 10.1212/cpj.0000000000000095

40. Beech P, Greenhalgh J, Thornton M, et al. A survey of the practice of stroke doctors in developing transient ischaemic attack services in the UK. *Journal of evaluation in clinical practice* 2007;13(3):395-9. doi: 10.1111/j.1365-2753.2006.00714.x [published Online First: 2007/05/24]

41. Ranta A, Barber PA. Transient ischemic attack service provision: A review of available service models. *Neurology* 2016 doi: 10.1212/wnl.0000000000002339

For peer review only

Figure legends

Figure 1. Example survival curves and daily hazard rates for risk of major stroke in high and low risk patients.

High risk patient: male age 70-74, SBP 156mmHg, Speech disturbance without weakness, Duration of symptoms 60+ mins, not diabetic (resulting in ABCD and $ABCD^2$ scores of 5), undiagnosed AF, cholesterol 5.8. Low risk patient: male age 70-74, SBP 115mmHg, Speech disturbance without weakness, Duration of symptoms 0-9 mins, not diabetic (resulting in ABCD and $ABCD^2$ of scores 2), AF on warfarin, cholesterol 6.0.

Figure 2. Outcomes of service provision in terms of clinic appointment utilisation and guideline breaches.

OP = outpatient appointment; TIA = transient ischemic attack; LR = low risk; HR = high risk. Original and modified services (first two bars at each hospital) were those which were actually implemented in each hospital. The remaining weekend services are hypothetical. *Hospital 1 included 16 routine clinics, 5 days per week (original service), 17 in the modified service and 19 in the modified service with weekend working. †Hospital 2 included 12 routine clinics, 3 days per week (original service), and 14 in the modified service (which also included weekend working).

For peer review only

Tables

Table 1. Patient characteristics as modelled

**Patient characteristics**	
Age (mean±SD) (years)	74 ± 12
Gender (% female)	240 (48.9%)
Systolic blood pressure (mean ± SD) (mmHg)	
TIA mimic	143 ± 26
TIA	147 ± 22
Minor stroke	144 ± 18
Overall mean	145 ± 24
Final Diagnosis (Actual condition)	
High risk TIA	116 (23.6%)
Low risk TIA	46 (9.4%)
TIA mimic	294 (60.0%)
Minor stroke	34 (7.0%)
*Patients referral to hospital via their GP according to diagnosis**	
High risk TIA	84 (72.3%)
Low risk TIA/ TIA mimic	299 (87.9%)
Minor stroke	20 (57.4%)
Overall mean	402 (82.1%)

SD = Standard deviation; TIA = Transient ischemic attack; GP = general practitioner. *Expected numbers are rounded to the nearest integer, the apparent anomaly with the addition results from this rounding. High risk patients defined as an $ABCD^2$ score of >4

For peer review only

Table 2. Pattern of outpatient clinics for suspected TIA based on actual service provision at participating hospitals during patient recruitment period.

**Hospital and service**		**Number of routine clinic slots available by day of the week**					**Total clinics per year**	**Details of clinic allocation within the model**
Hospital 1	Original service*	2	4	4	4	2	724	All patients are assigned to the next available clinic slots in order of referral. Where two referrals are made in one day, high risk patients are given priority.
	Modified service*	4	4	2	3	4	769	Where two referrals are made in one day, high patients are given priority. One slot is reserved at the end of each clinic for high risk referrals. If the next high risk clinic slot is not within 24 hours of referral, an additional slot is made available (up to 1 per day). All other patients are assigned to the next available clinic slots in order of referral.
Hospital 2	Original service†	6	4	2	0	0	624	Patients are assigned to the next available outpatient clinic. Those high risk patients who cannot be seen within 24 hours are admitted (including those presenting at weekends).
	Modified service†	4 or 6‡	0	4	0	4	676	All high risk patients are seen on the ward as outpatients as required (including at weekends). Patients referred before 10am are seen at 5pm on the same day, patients referred after 10am are seen at 10am on the following day. All low risk patients seen at the next available clinic in order of referral.

*Clinics are divided among 4 specialists, each of whom were absent for approximately 7 weeks a year (annual leave). These clinics are assumed not to take place if the specialist is absent.

†Specialists were absent for approximately 7 weeks a year (annual leave). All absent clinicians were replaced by a specialist from another site within the Trust.

‡There is a 50% probability each week that either 4 or 6 clinic slots will be available.

For peer review only - http://bmjopen.bmj.com/site/about/guidelines.xhtml

Table 3. Costs, resource utilisation and outcomes (per year) of modifying TIA service provision in hospital 1.

	**Original service (16 clinic slots per week)**	**Modified service (17 clinic slots per week)**	**Weekend service + 17 clinic slots per week)**	**Weekend service + 15 clinic slots per week)**	**Weekend service + 13 clinic slots per week)**
Days operating per week	5 days	5 days	7 days	7 days	7 days
Total number of patients presenting	491	490	490	491	491
Cost of clinics used & unused	£340,000	£361,000	£366,000	£346,000	£325,000
Major strokes post TIA (mean, 99% CI)*	10.6 (10.4-10.8)	10.7 (10.5-10.9)	10.6 (10.4-10.8)	10.8 (10.6-11.0)	10.6 (10.4-10.8)
Post-stroke deaths (mean, 99% CI)*	3.0 (2.9-3.1)	3.0 (2.9-3.1)	3.0 (2.9-3.1)	3.1 (3.0-3.2)	3.0 (2.9-3.1)
Number of high risk breaches† (%)	103 (69%)	32 (21%)	6 (4%)	7 (5%)	8 (5%)
Number of low risk breaches‡ (%)	7 (2%)	4 (1%)	3 (1%)	13 (4%)	75 (22%)
Time from referral to clinic appointment for high risk patients in days (median, IQR)	1.15 (0.93-2.88)	0.85 (0.17-0.99)	0.68 (0.16-0.93)	0.70 (0.15-0.93)	0.86 (0.16-0.99)

TIA = transient ischemic attack; CI = confidence intervals; IQR = inter-quartile range. Of the ~490 patients in the model, 340 are considered low risk (294 TIA mimic; 46 low risk TIA) and 150 are considered high risk (116 high risk TIA; 34 minor stroke). *Point estimate and 99% quasi confidence interval reflecting the uncertainty from sampling in the model, not any uncertainty in model parameters. 99% was chosen because of multiple values were compared. †High risk breaches were defined as high risk patients not seen by a specialist within 24 hours of initial clinic referral.[12] ‡ Low risk breaches were defined as low risk patients not seen by a specialist within 7 days of initial clinic referral.

For peer review only - http://bmjopen.bmj.com/site/about/guidelines.xhtml

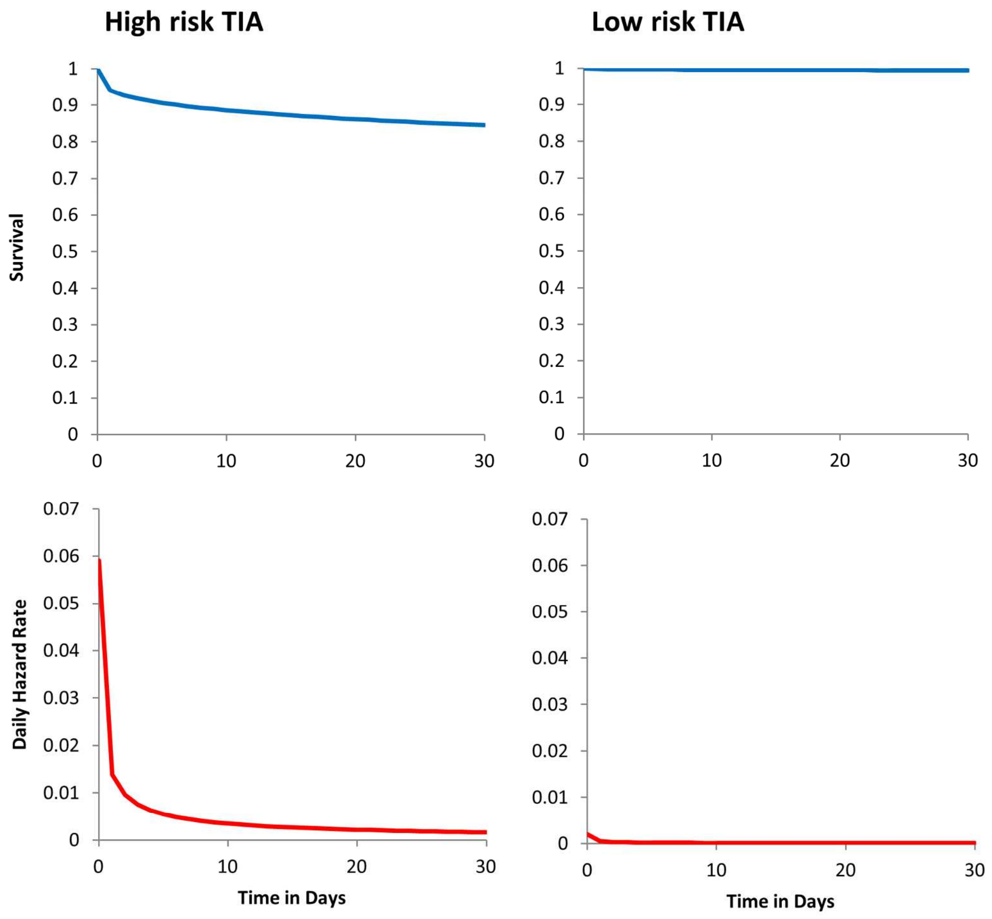

Figure 1. Example survival curves and daily hazard rates for risk of disabling stroke in high and low risk patients.

High risk patient: male age 70-74, SBP 156mmHg, Speech disturbance without weakness, Duration of symptoms 60+ mins, not diabetic (resulting in ABCD and ABCD2 scores of 5), undiagnosed AF, cholesterol 5.8. Low risk patient: male age 70-74, SBP 115mmHg, Speech disturbance without weakness, Duration of symptoms 0-9 mins, not diabetic (resulting in ABCD and ABCD2 of scores 2), AF on warfarin, cholesterol 6.0.

172x160mm (300 x 300 DPI)

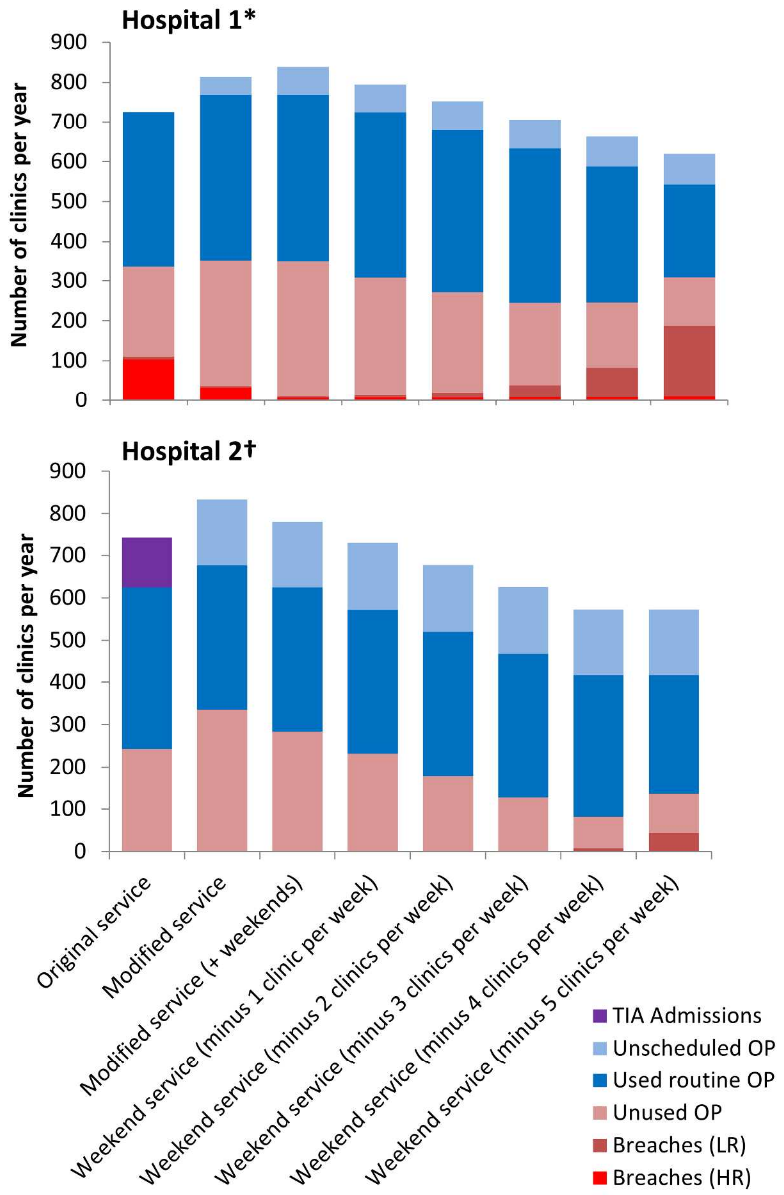

Figure 2. Outcomes of service provision in terms of clinic appointment utilisation and guideline breaches.

OP = outpatient appointment; TIA = transient ischemic attack; LR = low risk; HR = high risk. Original and modified services (first two bars at each hospital) were those which were actually implemented in each hospital. The remaining weekend services are hypothetical. *Hospital 1 included 16 routine clinics, 5 days per week (original service), 17 in the modified service and 19 in the modified service with weekend working. †Hospital 2 included 12 routine clinics, 3 days per week (original service), and 14 in the modified service (which also included weekend working).

172x260mm (300 x 300 DPI)

Online data supplement

For peer review only

Contents

1. **Extended methods**
2. **eFigure 1.** Care pathway for patients presenting with symptoms of transient ischemic attack
3. **eTable 1.** Sources of data included in the model
4. **eTable 2.** Resource unit costs included in the service provision model
5. **eTable 3.** Costs, resource utilisation and outcomes (per year) of modifying TIA service provision in hospital 2.
6. **References**

For peer review only

Extended methods

Setting and service design

The model was designed to assess the costs and clinical consequences arising from patients suspected of suffering an acute TIA and referred to two large urban hospitals in the West Midlands region of the UK. Both hospitals ran a specialist TIA outpatient service catering for approximately 500 patients with suspected stroke and TIA every year. The model parameters were defined by the characteristics of individuals attending these services and recruited to an observational study[1] during 2011 (table 1).

The availability of specialist TIA clinics was modelled on the basis of existing service provision at participating hospital sites during the study period. Patients could either be seen in traditional outpatient clinics, admitted, or seen on the ward on an outpatient basis. The original services were designed within the confines of available clinical staff and a lack of specialist cover at the weekends. Services were redesigned at each site during the study period and the impact of these changes was examined by modelling both the original and modified service. The number and distribution of clinics in each service are described in Table 2.

The impact of further adjustments to the modified service at each site was modelled to replicate the following scenarios:

a) The addition of high risk clinic slots for patients presenting on a Saturday and Sunday
b) Including a weekend service but reducing the number of routine weekday clinics, by up to 5 clinic slots (one patient per slot) per week.

Overview of the model

A discrete event simulation model was programmed in Delphi version 4 (Borland, San Francisco, CA, USA). It was adapted from a previously developed model and further detail regarding the general modelling framework can be found in the original report.[2] Briefly, the model generated a number of possible (virtual) patient histories which began at the onset of TIA (or TIA like) symptoms and followed the patient along the clinical pathway from initial presentation to referral, specialist TIA clinic attendance, treatment and lifetime follow-up for subsequent stroke morbidity and mortality. An essential feature of the model was that patients were sharing limited resources in the form of routine clinics.

For peer review only - http://bmjopen.bmj.com/site/about/guidelines.xhtml

The model estimated the impact of existing service provision, the effect of recent service change and the impact of further alterations to clinic availability on service costs/provision and subsequent stroke events, from a health services perspective.

Clinical pathways in the model

Following onset of an initial event, patients were assumed to contact either their general practitioner (GP) or attend the emergency department (ED) (eFigure 1, online supplemental material). The probability that a patient would choose a specific route was dependent on the type of patient (high/low risk), the time of day, and day of week, estimated using data from the Oxford Vascular Study.[3] Time taken to reach the service depended on the route chosen; where patients directly attended the ED, the time was sampled from a Weibull distribution fitted to data from the Newcastle Stroke Service.[2] Patients attending their GP were assumed to arrive within daytime clinic hours.[3] Following this initial contact with a healthcare professional, patients were referred to a specialist TIA outpatient clinic, seen on the ward (as an outpatient) or admitted as an inpatient. The type of referral was dependant on the specific hospital service provision, clinic availability and the level of risk of the attending patient, defined according to the $ABCD^2$ score.[4] During clinic attendance, it was assumed that the appropriate treatment would be initiated (i.e. blood pressure lowering, cholesterol lowering or antiplatelet therapy in accordance with guidelines) and a small proportion of patients (4.1%) would be deemed suitable for carotid endarterectomy.[5,6] Patients were assumed to take this treatment as prescribed and gain the full benefit in terms of stroke prevention.

Model population

All patients in the model were hypothetical, although they we based on data from real patients and hospitals. Overall rates of patient presentation were based on initial runs of the model and ensured a realistic distribution of final diagnoses which represented the study sample, previous literature[7] and expert opinion (60% TIA mimics, 33% genuine TIA, and 7% minor stroke). High and low risk TIAs were defined according to the $ABCD^2$ score.[4] In the absence of robust data for the distribution of new referrals through the week, it was assumed that new cases arrived uniformly.

Due to missing risk data in the sample population, the observed ratio of low risk/high risk TIA patients used as basecase in the model was supplemented in sensitivity analyses by estimates derived from those reported in previous studies[4,8,9] and the experience of stroke physicians from centres across the UK [personal communications, D Sims, G Ford and C Roffe]. The range of these estimates tested in this sensitivity analysis for impact on the model results was: 2.5:1 (high:low risk; base-case) to 1:1, 5:1 and 7:1.[4,8,9]

For peer review only

Follow-up and risk of repeat events

Hypothetical patients remained in the model until one year from symptom onset, after which the increased risk of repeat event returns close to normal,[10,11] unless they died or suffered a non-fatal disabling stroke. No distinction was made in the model between fatal strokes and non-fatal disabling strokes: these were labelled "major strokes". The risk of a repeat event (TIA or stroke) was dependent on the initial event (minor stroke, true TIA or mimic), the severity of that event (measured by $ABCD^2$ score) and other relevant risk factors such as age, presence of atrial fibrillation and medication prescribed.[4,6,12-17] Following a minor (non-disabling) stroke, patients remained in the model, but with an additional risk of mortality that could be reduced by appropriate treatment. Additional deaths from this cause were estimated and labelled in the model outputs as "post-stroke deaths". Modelled outputs are therefore derived from risk profile of the hypothetical patients, adjusting for the effects of treatments which would start at varying times in the different scenarios. Risks were modelled using a Weibull distribution for the time to event which allowed for a substantially increased risk in the short term, followed by a decreasing risk over time.[18] Examples of the modelled risk for two patients (with high and low risk characteristics) are given in figure 1.

The model was run 100 times for a total simulated time of 12 years in each run: the first year of the run was regarded as a "warm up" period to allow the system to reach a steady state before collecting data. Patients entering during the next 10 years were included in the model results, with the final year being necessary to allow for a one-year follow up time for all included patients. Results for patients entering the model during this last "follow up" period were not included, but such patients were modelled to ensure that the constraints on clinic availability were maintained. Results are presented in terms of annual costs and other outcomes.

Costs and outcomes

The model included costs of GP visits, transport by ambulance to the ED, ED attendance, outpatient clinics, hospital admission for TIA and stroke, surgery and therapy (eTable 1 and 2, online data supplement). Costs included in the model were taken from a combination of NHS reference costs,[19,20] and drug costs from the British National Formulary.[21] The price year for all costs was 2011-12.

The primary outcome was the number of expected major strokes occurring post TIA, based on risk analysis. Secondary outcomes included the overall service costs per year and attainment of national targets for TIA service provision. Target attainment was defined as the number of high and low risk ‘breaches’ which occurred in each service per year: high risk breaches were defined as a high risk

For peer review only

patient not seen by a specialist within 24 hours of initial referral.[5,22] Low risk breaches were defined as low risk patients not seen by a specialist within seven days of referral.[5,22] Further outcomes examined the median time from referral to specialist appointment, the total number of routine outpatient appointments available (used or unused) and any unscheduled outpatient appointments required (where high risk patients were assessed immediately on the ward).

All data are presented as means or medians ± standard deviation (SD), inter-quartile range (IQR) or 99% confidence intervals (CI), chosen because multiple values are compared. Percentages are given for the total population unless otherwise stated.

For peer review only

eFigure 1. Care pathway for patients presenting with symptoms of transient ischemic attack

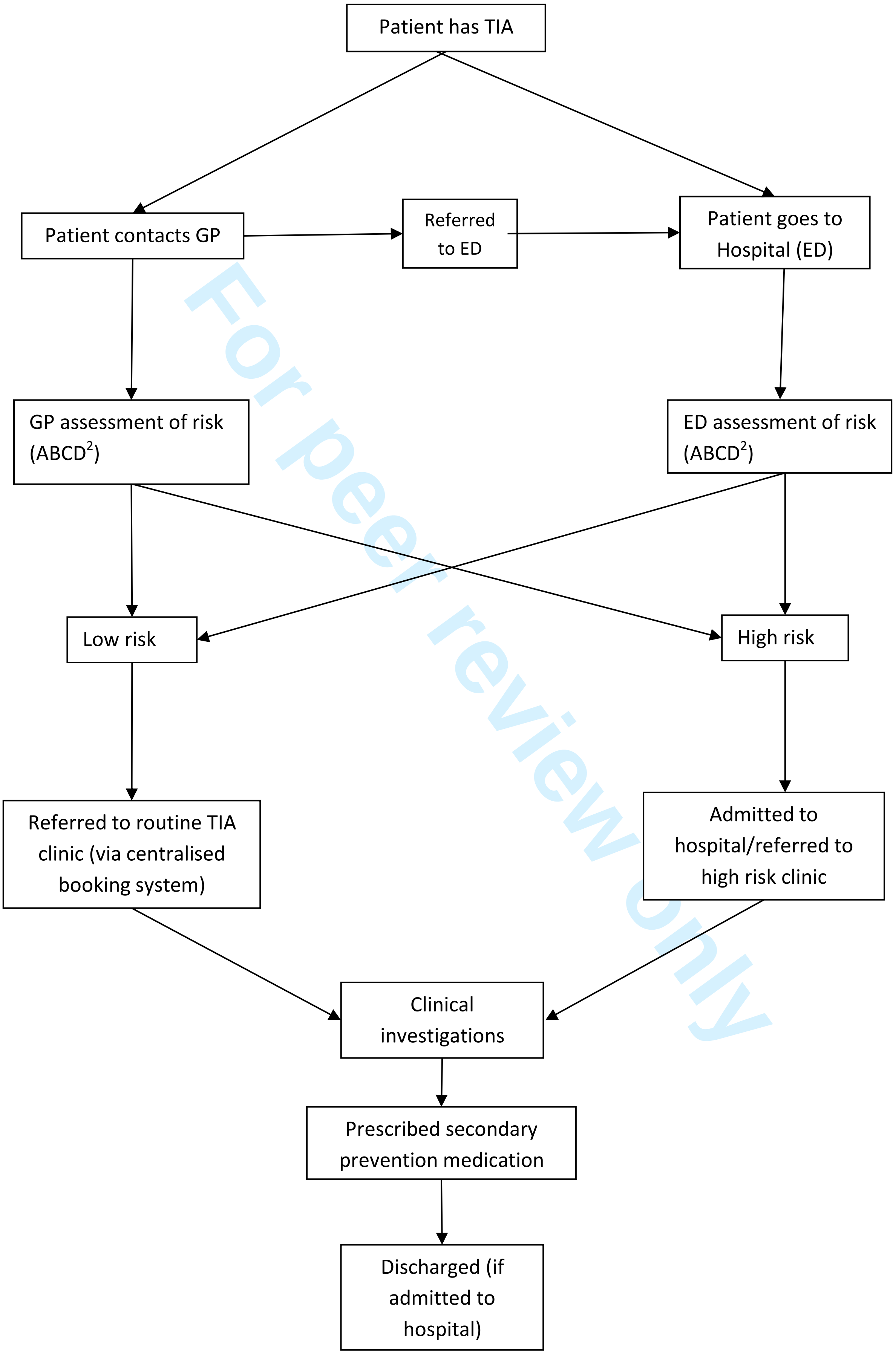

eTable 1. Sources of data included in the model

Model Inputs	Source	References
Schedule of Appointments<br>Casemix (mimic, TIA, minor stroke)	Participating hospital sites, Previous literature and the experience of stroke physicians from centres across the UK (personal communications, D Sims, G Ford and C Roffe)	8,9
Distribution of patient ages	Data from the OXVASC study adjusted to England and Wales population	23
Patient characteristics (gender, components of ABCD[2] score, current medication, AF status, cholesterol level, diabetes conditional on age as appropriate)	Data from the CLAHRC optimisation of the management of stroke and TIA study	1
Patient choice to contact GP or ambulance	OXVASC data	3
Time for patient to reach GP	Assumptions sustained from SDO model	2
Time for patient to reach service using ambulance	Data from Northumbria Ambulance Service	2
"Other cause" death rates	Government Actuary's Department & ONS, Health Statistics Quarterly	24-26
Baseline risk of repeat events	OXVASC data	23
Factors affecting risk of repeat events	Previous literature	12-17,23

OXVASC = OXford VASCular study; CLAHRC = Collaborations for Leadership in Applied Health Research and Care; GP = General Practitioner; ABCD[2] = Age, Blood pressure, Clinical features, Duration and Diabetes; AF = Atrial Fibrillation

For peer review only

eTable 2. Resource unit costs included used in the service provision model.

Item	Cost (£)	Notes	Data source (reference)
Use of an ambulance	230		[19]
General practitioner consultation	33		[20]
Emergency department consultation	143	Category 2 investigation not leading to admitted	[19]
Outpatient appointment	212		[19]
Repeat major stroke	3127		[19]
Assessment for carotid endarterectomy	50	Additional cost of ultrasound only	[19]
Carotid endarterectomy surgery	3923		[19]
Stroke after carotid endarterectomy	3127	As for repeat stroke – small numbers	[19]
Hospital Admission	413		[19]
MRI	92	Applied to 20% of patients at outpatient appointment or hospital admission	[19]
Initiation of antiplatelet therapy*	0.485	300mg loading (from pack of 56 aspirin)	[21]
Antiplatelet mono therapy (per week)*	0.1213	75mg daily from pack of 56 aspirin	[21]
Antiplatelet dual therapy (per week)*	1.0683	Mono plus clopidogrel 75 mg od	[21]
Warfarin (per week)*	0.2150	3mg daily	[21]
Statin (per week)*	0.2925	Simvastatin 40mg daily (from pack of 28)	[21]
Antihypertensive*	0.2025	Bendrofulmethiazide 2.5mg daily	[21]

*Costs taken from the British National Formulary (November 2012) [21]

For peer review only

eTable 3. Costs, resource utilisation and outcomes (per year) of modifying TIA service provision in hospital 2.

	**Original service (12 clinic slots per week)**	**Modified service (14 clinic slots per week)**	**New service (13 clinic slots per week)**	**New service (11 clinic slots per week)**	**New service (9 clinic slots per week)**
Days operating per week	7 days	7 days	7 days	7 days	7 days
Total number of patients presenting	490	490	490	490	490
Cost of clinics used & unused	£368,000	£363,000	£353,000	£329,000	£304,000
Major strokes post TIA (mean, 99% CI)*	10.7 (10.5-10.9)	10.4 (10.2-10.5)	10.7 (10.5-10.9)	10.6 (10.5-10.8)	10.6 (10.4-10.8)
Post-stroke deaths (mean, 99% CI)*	3.0 (2.9-3.1)	3.0 (2.9-3.1)	3.0 (2.9-3.1)	3.1 (3.0-3.2)	3.0 (2.9-3.1)
Number of high risk breaches† (%)	0 (0.0%)	0 (0.0%)	0 (0.0%)	0 (0.0%)	0 (0.0%)
Number of low risk breaches‡ (%)	0 (0.0%)	0 (0.0%)	0 (0.0%)	1 (0.3%)	44 (12.9%)
Time from referral to clinic appointment for high risk patients in days (median, IQR)	0.85 (0.74-0.94)	0.75 (0.08-0.88)	0.75 (0.08-0.88)	0.75 (0.08-0.88)	0.75 (0.08-0.88)

TIA = transient ischemic attack; CI = confidence intervals; IQR = inter-quartile range

Of the ~490 patients in the model, 340 are considered low risk (294 TIA mimic; 46 low risk TIA) and 150 are considered high risk (116 high risk TIA; 34 minor stroke)

*Point estimate and 99% quasi confidence interval reflecting the uncertainty from sampling in the model, not any uncertainty in model parameters. 99% was chosen because of multiple values were compared

†High risk breaches were defined as high risk patients not seen by a specialist within 24 hours of initial clinic referral. These could not occur with the service pattern, as all high risk patients were admitted in the original service, and seen on the ward in the modified service.

‡ Low risk breaches were defined as low risk patients not seen by a specialist within 7 days of initial clinic referral.

For peer review only

References

1. Sheppard JP, Mellor RM, Bailey SM, et al. Protocol for an observation and implementation study investigating optimisation of the management of stroke and transient ischaemic attack (TIA). *BMJ open* 2012; **2**(3).

2. Mant J, Barton P, McManus RJ, Rothwell PM. What is the Optimum Model of Service Delivery for Transient Ischaemic Attack?: National Co-ordinating Centre for NHS Service Delivery and Organisation R&D (NCCSDO), 2008.

3. Lasserson DS, Chandratheva A, Giles MF, Mant D, Rothwell PM. Influence of general practice opening hours on delay in seeking medical attention after transient ischaemic attack (TIA) and minor stroke: prospective population based study. *BMJ (Clinical research ed)* 2008; **337**: a1569.

4. Rothwell PM, Giles MF, Flossmann E, et al. A simple score (ABCD) to identify individuals at high early risk of stroke after transient ischaemic attack. *Lancet* 2005; **366**(9479): 29-36.

5. National Institute for Health and Clinical Excellence (NICE). Diagnosis and intial management of acute stroke and transient ischemic attack (TIA), 2008.

6. Rothwell PM, Eliasziw M, Gutnikov SA, Warlow CP, Barnett HJ. Endarterectomy for symptomatic carotid stenosis in relation to clinical subgroups and timing of surgery. *Lancet* 2004; **363**(9413): 915-24.

7. Brazzelli M, Shuler K, Quayyum Z, et al. Clinical and imaging services for TIA and minor stroke: results of two surveys of practice across the UK. *BMJ open* 2013; **3**(8).

8. Tsivgoulis G, Stamboulis E, Sharma VK, et al. Multicenter external validation of the ABCD2 score in triaging TIA patients. *Neurology* 2010; **74**(17): 1351-7.

9. Asimos AW, Johnson AM, Rosamond WD, et al. A multicenter evaluation of the ABCD2 score's accuracy for predicting early ischemic stroke in admitted patients with transient ischemic attack. *Annals of emergency medicine* 2010; **55**(2): 201-10.e5.

10. Coull AJ, Lovett JK, Rothwell PM. Population based study of early risk of stroke after transient ischaemic attack or minor stroke: implications for public education and organisation of services. *BMJ (Clinical research ed)* 2004; **328**(7435): 326.

11. Lovett JK, Dennis MS, Sandercock PA, Bamford J, Warlow CP, Rothwell PM. Very early risk of stroke after a first transient ischemic attack. *Stroke; a journal of cerebral circulation* 2003; **34**(8): e138-40.

12. Collaborative meta-analysis of randomised trials of antiplatelet therapy for prevention of death, myocardial infarction, and stroke in high risk patients. *BMJ (Clinical research ed)* 2002; **324**(7329): 71-86.

13. Aguilar MI, Hart R. Oral anticoagulants for preventing stroke in patients with non-valvular atrial fibrillation and no previous history of stroke or transient ischemic attacks. *The Cochrane database of systematic reviews* 2005; (3): Cd001927.

14. Baigent C, Keech A, Kearney PM, et al. Efficacy and safety of cholesterol-lowering treatment: prospective meta-analysis of data from 90,056 participants in 14 randomised trials of statins. *Lancet* 2005; **366**(9493): 1267-78.

For peer review only

15. Ebrahim S, Harwood R. Stroke epidemiology, evidence, and clinical practice. Oxford University Press: Oxford; 1999.

16. MacMahon S. BP and the risks of CV disease. In: Swales J, ed. Textbook of hypertension. Oxford: Blackwell Scientific Publications; 1994.

17. Wolf PA, Abbott RD, Kannel WB. Atrial fibrillation as an independent risk factor for stroke: the Framingham Study. *Stroke; a journal of cerebral circulation* 1991; **22**(8): 983-8.

18. Rothwell PM, Giles MF, Chandratheva A, et al. Effect of urgent treatment of transient ischaemic attack and minor stroke on early recurrent stroke (EXPRESS study): a prospective population-based sequential comparison. *Lancet* 2007; **370**(9596): 1432-42.

19. Department of Health. 2011–12 Reference Costs. 2013. https://www.gov.uk/government/publications/2011-12-reference-costspublication (accessed June 2013).

20. Curtis L. Unit Costs in Health and Social Care 2011. 2012. http://www.pssru.ac.uk/project-pages/unit-costs/2011/index.php. (accessed November 2012).

21. British National Formulary (BNF). Pharmaceutical Press. 2012. https://www.bnf.org/ (accessed November 2012).

22. Intercollegiate Stroke Working Party. National clinical guideline for stroke. London: Royal College of Physicians, 2012.

23. Rothwell PM, Coull AJ, Giles MF, et al. Change in stroke incidence, mortality, case-fatality, severity, and risk factors in Oxfordshire, UK from 1981 to 2004 (Oxford Vascular Study). *Lancet* 2004; **363**(9425): 1925-33.

24. Government Actuary's Department. Interim life tables. http://www.gad.gov.uk/, 2006.

25. Health Statistics Quarterly. Deaths by age, sex and underlying cause, 2005 registrations. http://www.statistics.gov.uk/statbase/, 2007.

26. Office for National Statistics. Interim Life Tables for England. http://www.ons.gov.uk/ons/publications/index.html, 2011.

For peer review only

CHEERS Checklist

Items to include when reporting economic evaluations of health interventions

The **ISPOR CHEERS Task Force Report**, *Consolidated Health Economic Evaluation Reporting Standards (CHEERS)—Explanation and Elaboration: A Report of the ISPOR Health Economic Evaluations Publication Guidelines Good Reporting Practices Task Force*, provides examples and further discussion of the 24-item CHEERS Checklist and the CHEERS Statement. It may be accessed via the *Value in Health* or via the ISPOR Health Economic Evaluation Publication Guidelines – CHEERS: Good Reporting Practices webpage: http://www.ispor.org/TaskForces/EconomicPubGuidelines.asp

Section/item	Item No	Recommendation	Reported on page No/ line No
**Title and abstract**			
Title	1	Identify the study as an economic evaluation or use more specific terms such as "cost-effectiveness analysis", and describe the interventions compared.	
Abstract	2	Provide a structured summary of objectives, perspective, setting, methods (including study design and inputs), results (including base case and uncertainty analyses), and conclusions.	
**Introduction**			
Background and objectives	3	Provide an explicit statement of the broader context for the study.<br>Present the study question and its relevance for health policy or practice decisions.	
**Methods**			
Target population and subgroups	4	Describe characteristics of the base case population and subgroups analysed, including why they were chosen.	
Setting and location	5	State relevant aspects of the system(s) in which the decision(s) need(s) to be made.	
Study perspective	6	Describe the perspective of the study and relate this to the costs being evaluated.	
Comparators	7	Describe the interventions or strategies being compared and state why they were chosen.	
Time horizon	8	State the time horizon(s) over which costs and consequences are being evaluated and say why appropriate.	
Discount rate	9	Report the choice of discount rate(s) used for costs and outcomes and say why appropriate.	
Choice of health outcomes	10	Describe what outcomes were used as the measure(s) of benefit in the evaluation and their relevance for the type of analysis performed.	
Measurement of effectiveness	11a	*Single study-based estimates:* Describe fully the design features of the single effectiveness study and why the single study was a sufficient source of clinical effectiveness data.	

For peer review only

	11b	*Synthesis-based estimates:* Describe fully the methods used for identification of included studies and synthesis of clinical effectiveness data.	
Measurement and valuation of preference based outcomes	12	If applicable, describe the population and methods used to elicit preferences for outcomes.	
Estimating resources and costs	13a	*Single study-based economic evaluation:* Describe approaches used to estimate resource use associated with the alternative interventions. Describe primary or secondary research methods for valuing each resource item in terms of its unit cost. Describe any adjustments made to approximate to opportunity costs.	
	13b	*Model-based economic evaluation:* Describe approaches and data sources used to estimate resource use associated with model health states. Describe primary or secondary research methods for valuing each resource item in terms of its unit cost. Describe any adjustments made to approximate to opportunity costs.	
Currency, price date, and conversion	14	Report the dates of the estimated resource quantities and unit costs. Describe methods for adjusting estimated unit costs to the year of reported costs if necessary. Describe methods for converting costs into a common currency base and the exchange rate.	
Choice of model	15	Describe and give reasons for the specific type of decision-analytical model used. Providing a figure to show model structure is strongly recommended.	
Assumptions	16	Describe all structural or other assumptions underpinning the decision-analytical model.	
Analytical methods	17	Describe all analytical methods supporting the evaluation. This could include methods for dealing with skewed, missing, or censored data; extrapolation methods; methods for pooling data; approaches to validate or make adjustments (such as half cycle corrections) to a model; and methods for handling population heterogeneity and uncertainty.	
**Results**			
Study parameters	18	Report the values, ranges, references, and, if used, probability distributions for all parameters. Report reasons or sources for distributions used to represent uncertainty where appropriate. Providing a table to show the input values is strongly recommended.	
Incremental costs and outcomes	19	For each intervention, report mean values for the main categories of estimated costs and outcomes of interest, as well as mean differences between the comparator groups. If applicable, report incremental cost-effectiveness ratios.	
Characterising uncertainty	20a	*Single study-based economic evaluation:* Describe the effects of sampling uncertainty for the estimated incremental cost and incremental effectiveness parameters, together with the impact	

For peer review only

		of methodological assumptions (such as discount rate, study perspective).	
	20b	*Model-based economic evaluation:* Describe the effects on the results of uncertainty for all input parameters, and uncertainty related to the structure of the model and assumptions.	
Characterising heterogeneity	21	If applicable, report differences in costs, outcomes, or cost-effectiveness that can be explained by variations between subgroups of patients with different baseline characteristics or other observed variability in effects that are not reducible by more information.	
**Discussion**			
Study findings, limitations, generalisability, and current knowledge	22	Summarise key study findings and describe how they support the conclusions reached. Discuss limitations and the generalisability of the findings and how the findings fit with current knowledge.	
**Other**			
Source of funding	23	Describe how the study was funded and the role of the funder in the identification, design, conduct, and reporting of the analysis. Describe other non-monetary sources of support.	
Conflicts of interest	24	Describe any potential for conflict of interest of study contributors in accordance with journal policy. In the absence of a journal policy, we recommend authors comply with International Committee of Medical Journal Editors recommendations.	

For consistency, the CHEERS Statement checklist format is based on the format of the CONSORT statement checklist

The **ISPOR CHEERS Task Force Report** provides examples and further discussion of the 24-item CHEERS Checklist and the CHEERS Statement. It may be accessed via the *Value in Health* link or via the ISPOR Health Economic Evaluation Publication Guidelines – CHEERS: Good Reporting Practices webpage: http://www.ispor.org/TaskForces/EconomicPubGuidelines.asp

The citation for the CHEERS Task Force Report is:

Husereau D, Drummond M, Petrou S, et al. Consolidated health economic evaluation reporting standards (CHEERS)—Explanation and elaboration: A report of the ISPOR health economic evaluations publication guidelines good reporting practices task force. Value Health 2013;16:231-50.

For peer review only

BMJ Open

When has service provision for transient ischemic attack improved enough? A discrete event simulation economic modelling study

Journal:	*BMJ Open*
Manuscript ID	bmjopen-2017-018189.R2
Article Type:	Research
Date Submitted by the Author:	12-Oct-2017
Complete List of Authors:	Barton, Pelham; University of Birmingham, Health Economics Unit<br>Sheppard, James; University of Oxford, Nuffield Department of Primary Care Health Sciences<br>Penaloza, Cristina; University of Birmingham, School of Health and Population Sciences<br>Jowett, Sue; University of Birmingham, Health Economics Unit<br>Ford, Gary; Oxford University Hospitals NHS Foundation Trust, Oxford Academic Health Science Network<br>Lasserson, Daniel; Oxford University Hospitals NHS Foundation Trust, NIHR Oxford Biomedical Research Centre<br>Mant, Jonathan; University of Cambridge, General Practice and Primary Care Research Unit<br>Mellor, Ruth; NHS Lanarkshire, Department of Public Health<br>Quinn, Tom; St George's Univesity of London & Kingston University, Joint Faculty of Health, Social Care & Education<br>Rothwell, Peter; Stroke Prevention Research Unit, Nuffield Department of Clinical Neurosciences<br>Sandler, David; Heart of England NHS Foundation Trust,<br>Sims, Don; University Hospitals Birmingham NHS Foundation Trust, Stroke Medicine<br>McManus, Richard; University of Oxford, Dept of Primary Care Health Sciences
**Primary Subject Heading**:	Health economics
Secondary Subject Heading:	Cardiovascular medicine, Neurology
Keywords:	Economic modelling, Stroke < NEUROLOGY, Secondary Care, Secondary prevention, Health services

SCHOLARONE™
Manuscripts

When has service provision for transient ischemic attack improved enough? A discrete event simulation economic modelling study

Modelling changes to service provision for transient ischemic attack

Barton, P.,[1] Sheppard, J.P.,[2] Penaloza-Ramos, M.C.,[1] Jowett, S.,[1] Ford, G.A.,[3] Lasserson, D.,[4] Mant, J.,[5] Mellor, R.M.,[6] Quinn, T.,[7] Rothwell, P.M.,[8] Sandler, D.,[9] Sims, D.,[10] McManus, R.J.[2] on behalf of the BBC CLAHRC investigators

[1]Health Economics Unit, University of Birmingham, Edgbaston, Birmingham, UK

[2]Nuffield Department of Primary Care Health Sciences, NIHR School for Primary Care Research, University of Oxford, Oxford, Oxfordshire, UK

[3]Oxford Academic Health Science Network, Oxford, Oxfordshire, UK

[4]Nuffield Department of Medicine, University of Oxford, Oxford, Oxfordshire, UK

[5]Primary Care Unit, University of Cambridge, Cambridge, Cambridgeshire, UK,

[6]Department of Public Health, NHS Lanarkshire, Kirklands, Fallside Road, Bothwell, UK

[7]Faculty of Health, Social Care and Education, St George's, University of London & Kingston University, London, UK

[8]Nuffield Department of Clinical Neurosciences, University of Oxford, Oxfordshire, UK.

[9]Heart of England NHS Foundation Trust, Birmingham, West Midlands, UK

[10]University Hospitals Birmingham NHS Foundation Trust, Birmingham, West Midlands, UK

Corresponding Author: James Sheppard

Address: Nuffield Department of Primary Care Health Sciences, University of Oxford, Radcliffe Primary Care, Radcliffe Observatory Quarter, Woodstock Road, Oxford, OX2 6GG

Email: james.sheppard@phc.ox.ac.uk

For peer review only

For peer review only - http://bmjopen.bmj.com/site/about/guidelines.xhtml

Telephone: +44 (0)1865 617192

Fax: +44 (0)1865 289287

Word count: 3,343 (not including abstract, tables, figures or references)

Key words: Economic modelling, stroke, secondary care, secondary prevention, health services

For peer review only

Abstract

Objectives: The aim of this study was to examine the impact of transient ischaemic attack service modification in two hospitals on costs and clinical outcomes.

Design: Discrete event simulation model using data from routine electronic health records from 2011.

Participants: Patients with suspected TIA were followed from symptom onset to presentation, referral to specialist clinics, treatment and subsequent stroke.

Interventions: Included existing versus previous (less same day clinics) and hypothetical service reconfiguration (7-day service with less availability of clinics per day).

Outcome measures: The primary outcome of the model was the prevalence of major stroke after TIA. Secondary outcomes included service costs (including those of treating subsequent stroke) and time to treatment and attainment of national targets for service provision (proportion of high risk patients [according to $ABCD^2$ score] seen within 24 hours).

Results: The estimated costs of previous service provision for 490 patients (aged 74±12 years, 48.9% female and 23.6% high risk) per year at each site were £340,000 and £368,000 respectively. This resulted in 31% of high risk patients seen within 24 hours of referral (47/150) with a median time from referral to clinic attendance/treatment of 1.15 days (IQR 0.93-2.88). The costs associated with the existing and hypothetical services decreased by £5,000 at one site and increased £21,000 at the other site. Target attainment was improved to 79% (118/150). However, the median time to clinic attendance was only reduced to 0.85 days (IQR 0.17-0.99) and thus no appreciable impact on the modelled incidence of major stroke was observed (10.7 per year, 99%CI 10.5-10.9 [previous service] vs. 10.6 per year, 99%CI 10.4-10.8 [existing service]).

Conclusions: Reconfiguration of services for TIA is effective at increasing target attainment, but in services which are already working efficiently (treating patients within 1-2 days), it has little estimated impact on clinical outcomes and increased investment may not be worthwhile.

For peer review only - http://bmjopen.bmj.com/site/about/guidelines.xhtml

Word count: 306

For peer review only

For peer review only - http://bmjopen.bmj.com/site/about/guidelines.xhtml

Strengths and limitations of this study

- This study focused on two hospitals within the West Midlands, UK, so the results are likely to be representative of these hospitals, but not necessarily other centres in the UK and across the world.
- Modelling allows estimation of service 'unknowns' which requires pre-programming and therefore simplification of certain service intricacies. Thus, the services modelled represent stylised versions of the actual services offered by participating hospitals.
- Costs relate to standard weekday services and it is not clear whether those actually incurred would be increased by provision of services at the weekend.
- The potential costs of implementation of new services, for example those for weekend services, were unavailable and so the costs presented here may have been underestimated.

For peer review only

Background

Transient ischaemic attack (TIA) is common, with incidences of 15-83 patients per 100,000 population recorded across the world;[1-5] in the UK, it affects approximately 50 patients per 100,000 population.[6] TIA is important because it represents a significant risk factor for future stroke with around 8% suffering an event within 7 days, and 12% within a month without preventative therapy.[7 8]

Rapid recognition and treatment of TIA patients at high risk of having a subsequent stroke is important because simple interventions, such as early prescription of preventative medications, can substantially reduce the risk of stroke following TIA, and existing evidence suggests that earlier intervention may be better.[9 10] In the UK, guidelines[11 12] recommend that patients at high risk of recurrent stroke (defined using the $ABCD^2$ score of >4)[10] are seen within 24 hours of symptom onset and all other patients are seen within 7 days. A recent UK audit suggests there has been significant improvement in attainment of these targets: 45% of high risk outpatients and 60% of inpatients now receive treatment on the same day as referral, compared with just 10% and 33% four years earlier.[13] This improved target attainment has been achieved through a variety of approaches, with some centres admitting more patients for assessment and treatment and others designing services with excess routine clinic capacity.[14]

Service reconfiguration remains ongoing in routine clinical practice and it is unclear what impact such changes have on service costs and clinical outcomes. This study used discrete event simulation modelling to assess the impact of TIA service reconfiguration in two large urban hospitals with different approaches to service provision.

Methods

An extended methods section can be found in the online data supplement.

For peer review only

Setting and service design

The model was designed to assess the costs and clinical consequences arising from patients suspected of suffering an acute TIA, referred to two large urban hospitals in the West Midlands region of the UK. Both hospitals ran a specialist TIA outpatient service catering for approximately 500 patients with suspected TIA and minor stroke every year. The model parameters were defined by the characteristics of individuals attending these services, recruited to an observational study[15] during 2011 (table 1).

The availability of specialist TIA clinics was modelled on the basis of existing service provision at participating hospital sites during the study period. Patients could either be seen in traditional outpatient clinics, admitted, or seen on the ward on an outpatient basis. The original services were designed within the confines of available clinical staff and in particular a lack of specialist cover at the weekends. Services were redesigned at each site during the study period and the impact of these changes on cost and outcomes was examined by modelling both the original and modified service. The number and distribution of clinics in each service are described in table 2.

The impact of further hypothetical adjustments to the modified service at each site was modelled to replicate the following scenarios:

a) The addition of high risk clinic slots for patients presenting on a Saturday and Sunday
b) Including a weekend service but reducing the number of routine weekday clinics, by up to 5 clinic slots (one patient per slot) per week.

Overview of the model

A discrete event simulation model was programmed in Delphi version 4 (Borland, San Francisco, CA, USA). It was adapted from a previously developed model and further detail regarding the general modelling framework can be found in the original report.[16] Briefly, the model generated a

For peer review only

For peer review only - http://bmjopen.bmj.com/site/about/guidelines.xhtml

number of possible (virtual) patient histories which began at the onset of TIA (or TIA like) symptoms and followed the patient along the clinical pathway from initial presentation to follow-up for subsequent stroke morbidity and mortality. An essential feature of the model was that patients were sharing limited resources in the form of routine clinics.

Clinical pathways in the model

Following onset of an initial event, patients were assumed to contact either their general practitioner (GP) or attend the emergency department (ED) (eFigure 1, online supplemental material). The probability that a patient would choose a specific route was dependent on the type of patient (high/low risk), the time of day, and day of week, estimated using data from the Oxford Vascular Study.[17] Following this initial contact with a healthcare professional, patients were referred to a specialist TIA outpatient clinic, seen on the ward (as an outpatient) or admitted as an inpatient. The type of referral was dependant on the specific hospital service provision, clinic availability and the level of risk of the attending patient, defined according to the $ABCD^2$ score.[10] During clinic attendance, it was assumed that the appropriate treatment would be initiated (i.e. blood pressure lowering, cholesterol lowering or antiplatelet therapy in accordance with guidelines) and a small proportion of patients (4.1%) would be treated with carotid endarterectomy.[12 18] Patients were assumed to take this treatment as prescribed and gain the full benefit in terms of stroke prevention.

Model population

All patients in the model were hypothetical, although they were based on data from real patients and hospitals. Overall rates of patient presentation were based on initial runs of the model and ensured a realistic distribution of final diagnoses which represented the study sample, previous literature[14] and expert opinion (60% TIA mimics, 33% genuine TIA, and 7% minor stroke). High and low risk TIAs were defined according to the $ABCD^2$ score.[10] The observed ratio of low risk/high risk TIA patients used in the base-case of the model was supplemented in sensitivity analyses by estimates

For peer review only - http://bmjopen.bmj.com/site/about/guidelines.xhtml

derived from those reported in previous studies[10 19 20] and the experience of stroke physicians from centres across the UK [personal communications, D Sims, G Ford and C Roffe]. The range of these estimates tested in this sensitivity analysis for impact on the model results was: 2.5:1 (high:low risk; base-case) to 1:1, 5:1 and 7:1.[10 19 20]

Follow-up and risk of repeat events

Hypothetical patients remained in the model until one year from symptom onset, after which the increased risk of repeat event returns close to normal,[7 8] unless they died or suffered a non-fatal disabling stroke. No distinction was made in the model between fatal strokes and non-fatal disabling strokes: these were labelled "major strokes". The risk of a repeat event (TIA or stroke) was dependent on the type of initial event (minor stroke, true TIA or mimic), the $ABCD^2$ based risk prediction of a subsequent event and other relevant risk factors such as age, presence of atrial fibrillation and medication prescribed.[10 18 21-26] Following a minor (non-disabling) stroke, patients remained in the model, but with an additional risk of mortality that could be reduced by appropriate treatment. Additional deaths from this cause were estimated and labelled in the model outputs as "post-stroke deaths". Modelled outputs are therefore derived from risk profile of the hypothetical patients, adjusting for the effects of treatments which would start at varying times in the different scenarios. Risks were modelled using a Weibull distribution for the time to event which allowed for a substantially increased risk in the short term, followed by a decreasing risk over time.[9] Examples of the modelled risk for two patients (with high and low risk characteristics) are given in figure 1. The model was run 100 times for a total simulated time of 12 years in each run.

Costs and outcomes

The model included costs of any GP visit (from presentation to referral), or transport by ambulance to the ED and ED attendance, outpatient clinics or hospital admission for TIA and stroke, surgery and therapy (eTables 1 and 2, online data supplement). Pre-hospital and treatment related costs were

For peer review only - http://bmjopen.bmj.com/site/about/guidelines.xhtml

the same for all options considered in this study since the focus was to compare comparative differences between modelled services caused by different clinic configurations. Costs included in the model were taken from a combination of NHS reference costs[27 28] and drug costs from the British National Formulary[29] and were modelled from a health services perspective. The price year for all costs was 2011-12.

The primary outcome was the number of expected major strokes occurring post TIA, based on risk analysis. Secondary outcomes included the overall costs of service provision per year and attainment of national targets for TIA service provision. Target attainment was defined as the number of high and low risk 'breaches' which occurred in each service per year: high risk breaches were defined as a high risk patient not seen by a specialist within 24 hours of initial referral.[11 12] Low risk breaches were defined as low risk patients not seen by a specialist within seven days of referral.[11 12] Further outcomes examined the median time from referral to specialist appointment, the total number of routine outpatient appointments available (used or unused) and any unscheduled outpatient appointments required (where high risk patients were assessed immediately on the ward).

All data are presented as means or medians ± standard deviation (SD), inter-quartile range (IQR) or 99% confidence intervals (CI), chosen because multiple values are compared. Percentages are given for the total population unless otherwise stated.

Results

TIA service usage

The model estimated a total of 490 patients would be referred to specialist TIA clinics each year at each hospital site (mean age 74±12 years; 48.9% female [table 1]). Of these, approximately 34 (7%) patients would be considered to have minor stroke and 162 (33%) would subsequently be considered to have confirmed TIA: the numbers classified as high/low risk, were 116/46, a ratio of

For peer review only - http://bmjopen.bmj.com/site/about/guidelines.xhtml

2.5:1. All subsequent results were robust to adjustment of this high:low risk ratio in the sensitivity analyses.

Costs and outcomes of original TIA service provision

The baseline incidence of major strokes post TIA was 10.6 per year (99% CI 10.4-10.8) in hospital 1 and 10.7 per year (99% CI 10.5-10.9) in hospital 2. The cost of providing TIA services at both sites ranged from £340,000-£368,000 per year (table 3 and eTable 3). Services included the provision of 624-724 specialist week day clinic slots per year. In hospital 1, few high risk patients were seen within 24 hours of referral (47/150 patients [31%]) and the median time from referral to clinic appointment was 1.15 days (IQR 0.93-2.88). In hospital 2, where all high risk TIAs were admitted (a target attainment of 100%), the median time from referral to being seen and treated by a specialist was 0.85 days (IQR 0.74-0.94).

Impact of service modification

Observed service modification increased the number of high risk patients seen within 24 hours in hospital 1 (118/150; guideline target attainment of 79%), with increased service costs (increased from £344,000 to £366,000 per year) due to an increase in the number of unused routine clinic appointments (225 to 316 unused clinic slots per year) (figure 2). This increase in target attainment, resulted in a median time from referral to clinic appointment of 0.85 days (IQR 0.17-0.99); the incidence of major strokes post TIA remained largely unchanged (10.6 per year, 99% CI 10.4-10.8 [modified service] vs. 10.7 per year, 99% CI 10.5-10.9 [original service]). Similar findings were observed at the second hospital site where costs were marginally reduced by not admitting patients with high risk TIA (decrease from £368,000 to £363,000 per year) but there was no change in the number of patients being seen within 24 hours (150/150; guideline target attainment of 100%), median time from referral to clinic appointment (0.75 days, IQR 0.08-0.88 [modified service] vs.

For peer review only - http://bmjopen.bmj.com/site/about/guidelines.xhtml

0.85 days, IQR 0.17-0.99 [original service]) or incidence of major strokes post TIA (10.4 per year, 99% CI 10.2-10.4 [modified service] vs. 10.7 per year, 99% CI 10.5-10.9 [original service]).

Potential optimal service provision

Modelling the impact of further hypothetical service reconfiguration, including a seven day service, suggested that it was possible to reduce service costs and the number of unused clinic appointments and improve guideline target attainment, but this had little impact on the incidence of major stroke post TIA or post-stroke deaths (table 3 and eTable 3). Figure 2 shows the trade-off between routine appointment provision, unscheduled appointments and high/low risk guideline breaches. Assuming unused appointments (pink), unscheduled appointments (in light blue), admissions (purple) and breaches (red [high risk breaches] and light red [low risk breaches]) are preferably avoidable outcomes of service provision, the optimal hypothetical service in hospital 1 involved a weekend service with approximately 17-18 routine clinic appointments per week. In hospital 2, where more unscheduled appointments were incurred, the optimal hypothetical service involved a weekend service with 10-11 routine clinic slots distributed across the week on Mondays, Wednesdays and Fridays (with high risk patients being seen in unscheduled appointments on the ward on any day of the week if necessary).

Discussion

The aim of this study was to model the impact of TIA service reconfiguration in two large urban hospitals and establish the impact on clinical outcomes (major strokes), costs, and clinic usage. Reconfiguration of services was found to be effective and appropriate for reducing resource (clinic slot) waste, and improving guideline target attainment with variable effects on costs. However, in hospitals where services, despite poor target attainment, had minimal patient delays, such as those studied here, significant reconfiguration had little impact on modelled clinical outcomes such as major stroke post TIA. These findings suggest that clinicians and service coordinators should be

For peer review only

cautious before initiating significant reconfiguration of services which are already seeing high risk patients within 1-2 days, unless there is an obvious need to free up resources which could be used for another purpose (e.g. managing other types of patients). Whilst national targets for care are important, consideration should be given to revising them to maximise the benefits of attainment whilst not incentivising small changes that might increase costs for no measurable clinical benefit.

Strengths and limitations

The present study used local hospital data to describe the characteristics of the presenting population and thus the results are likely to be representative of the hospitals studied, but not necessarily generalizable to other centres in the UK and across the world. Despite this, guideline target attainment in hospital 1 (original service configuration) was similar to that reported nationally (31% vs. 37% nationally)[13] which suggests that this hospital was more likely representative of centres across the UK.

Modelling allows estimation of service 'unknowns', such as the number of unscheduled clinic appointments required to meet guideline targets for high risk patients. This requires pre-programming with hypothetical patients and specific rules; therefore simplification of certain service intricacies is necessary. For example, the process by which high risk patients were allocated unscheduled clinic appointments was formalised such that a patient referred by the ED physician was assumed to have been seen by a stroke specialist within two hours. However, patients referred by their GP were assumed to be seen by the stroke specialist on the ward at 10am the following morning or 5pm the same day, whichever was later (but within 24 hours of initial referral). In reality, services are often adapted in response to a specific set of circumstances such as clinic room availability and thus the services modelled here, and the patients attending these services, can best be thought of as stylised versions of the actual services available and patients attending the hospitals in question.

For peer review only - http://bmjopen.bmj.com/site/about/guidelines.xhtml

The risk of recurrent stroke attributed to hypothetical patients in the model was based on an estimated ABCD^2 score.[10] The accuracy of this score for predicting recurrent stroke has been called into question with a recent systematic review showing it to have poor discrimination between those at low and high risk of early (7 day) stroke.[30] However, this was the score used and recommended in practice at the time these services were modelled and was used for all service configurations examined, so our relative comparisons are likely to remain valid, even if the absolute numbers may be subject to change.

The costs modelled here were taken from standard NHS reference costs[27 28] and included investigations, treatment and the cost of staff attending patients during each appointment/admission. Costs relate to standard weekday services and increased rates for weekend services were unavailable for this analysis. It is therefore likely that the costs of providing a weekend service may have been underestimated in the present analyses.

Findings in the context of previous literature and implications for practice

Economic modelling has previously been used for determining the impact of service reconfiguration in acute stroke[31-34] but studies examining optimal strategies for TIA service provision are less common. Those that exist, focus on whether or not patients should be admitted or be seen as an outpatient.[16 35 36] It is generally accepted that patient admission for TIA is inefficient and not cost-effective,[16 37-39] but there are a paucity of objective data comparing different configurations of outpatient service in the same setting, limited to questionnaire surveys of stroke physicians[14 40] and the Sentinel Stroke National Audit Programme (SSNAP) in the UK.[13] A recent review identified six models of care designed to avoid patient admission for TIA. These ranged from patient assessment and triage in the ED, a 24-hour nurse-led telephone advice service and the primary care triage system studied here (+/- electronic decision support).[41] The appropriate model of care will depend

For peer review only - http://bmjopen.bmj.com/site/about/guidelines.xhtml

on the local healthcare system, but consideration of how such a model is implemented in terms of service configuration is warranted, regardless of the system used.

The primary finding of this study was that whilst service reconfiguration can significantly improve guideline target attainment and potentially reduce service costs, such changes have little impact on the estimated prevalence of subsequent stroke, when services are already working efficiently. In the present study, even the largest service reconfiguration (introduction of a 7 day service) only reduced the median time from patient referral to clinic attendance and treatment by 0.3 days (8 hours). This enabled a large number of patients to be seen within 24 hours, improving guideline target attainment but had little impact on stroke risk. The recommendations for a 'see and treat within 24 hours' target for high risk patients were originally based on data from the EXPRESS study,[9] which showed that those patients seen and treated within 24 hours of initial referral had significantly better outcomes than those patients who were not. However, in the original service to which this optimal strategy was compared, the average time from referral to clinic appointment was 3 days (IQR 2-5 days) and time to first treatment was 20 days (IQR 8-53). The improvement in time to initiation of treatment was therefore 20 days, compared to an improvement of 8 hours seen in the present study, which explains the very different results in terms of clinical outcome.

Recent results from SSNAP show that 45% of high risk outpatients and 60% of high risk inpatients are seen and treated within 24 hours of referral, suggesting there is plenty of room for improvement. However, with a median time to first clinic appointment (and treatment) for all patients of just 2 days, the present analyses would suggest further improvements in guideline target attainment may have little impact on major stroke following TIA.

The modified services examined and proposed here may require the capacity to accommodate unscheduled clinic appointments, which is likely to have an opportunity cost affecting the quality of

For peer review only

other services running concurrently in the hospital and may result in increased costs with little tangible benefit. In particular, the ability to accommodate such appointments depends on the time of day at which the patient presents, the availability of specialist stroke physicians and scanning equipment required to make an accurate diagnosis, as well as the needs of competing services within the hospital (e.g. acute stroke services).

Conclusions

Reconfiguration of specialist services for TIA can be effective and appropriate for reducing costs, reducing the number of unused routine clinic appointments and increasing clinical guideline target attainment. However, where services are already working near optimal, such modification has little impact on clinical outcomes such as subsequent stroke. Clinicians and service coordinators should be cautious before initiating significant reconfiguration of services which are already seeing high risk patients within 1-2 days, although this might still be appropriate if resources within the hospital are freed up and could be used for an alternate purpose.

For peer review only

Declarations

Ethics approval and consent to participate

Approval for this project was obtained from the National Research Ethics Service (NRES) Committee, London – Queen Square (reference; 09/H0716/71).

Data sharing

The datasets analysed during the current study are available from the corresponding author on reasonable request.

Competing interests

The authors declare that they have no competing interests

Funding

This work was supported by the National Institute for Health Research (NIHR) as part of the Collaborations for Leadership in Applied Health Research and Care (CLAHRC) programme for Birmingham and Black Country. JPS held a Medical Research Council (MRC grant number MR/K022032/1) Strategic Skills Postdoctoral Fellowship and is now funded by the NIHR Oxford CLARHC. RJMcM holds an NIHR Professorship.

Authors' contributions

RJMcM, JM, SJ and PB had the original idea and gained the funding for the project. PB undertook the economic analyses. JPS and RMM were responsible for the data collection and JPS wrote the first draft of the manuscript with RJMcM and PB. PB, JPS, MCP, SJ, GAF, DL, JM, RMM, TQ, PMR, DS, DSi and RJM contributed to protocol development, refined the manuscript and approved the final version. RJMcM is the guarantor.

For peer review only

Acknowledgements

We would like to thank Amun Boyal, Kelly Rosborough and the staff at both participating hospitals for their assistance with recruitment and data collection. Thanks to Sheila Bailey and Anita Martin for their administrative support and Primary Care Clinical Research and Trials Unit for building and maintaining the study database.

Birmingham and Black Country Collaborations for Leadership in Applied Health Research and Care investigators include:

Peter Carr, Heart of England NHS Foundation Trust

Sheila Greenfield, Primary Care Clinical Sciences, University of Birmingham

Brin Helliwell, Lay member of Steering Group

Christina Nand, Lay member of Steering Group

Norman Phillips, Lay member of Steering Group

Rob Scott, Birmingham and Midland Eye Centre

Satinder Singh, Primary Care Clinical Sciences, University of Birmingham

Matthew Ward, West Midlands Ambulance Service NHS Trust

References

1. Cancelli I, Janes F, Gigli GL, et al. Incidence of transient ischemic attack and early stroke risk: validation of the ABCD2 score in an Italian population-based study. *Stroke; a journal of cerebral circulation* 2011;42(10):2751-7. doi: 10.1161/strokeaha.110.612705 [published Online First: 2011/08/13]

2. Correia M, Silva MR, Magalhaes R, et al. Transient ischemic attacks in rural and urban northern Portugal: incidence and short-term prognosis. *Stroke; a journal of cerebral circulation* 2006;37(1):50-5. doi: 10.1161/01.STR.0000195209.26543.8f [published Online First: 2005/12/03]

For peer review only - http://bmjopen.bmj.com/site/about/guidelines.xhtml

3. Feigin VL, Shishkin SV, Tzirkin GM, et al. A population-based study of transient ischemic attack incidence in Novosibirsk, Russia, 1987-1988 and 1996-1997. *Stroke; a journal of cerebral circulation* 2000;31(1):9-13. [published Online First: 2000/01/08]

4. Fonseca PG, Weiss PA, Harger R, et al. Transient ischemic attack incidence in joinville, Brazil, 2010: a population-based study. *Stroke; a journal of cerebral circulation* 2012;43(4):1159-62. doi: 10.1161/strokeaha.111.645101 [published Online First: 2011/12/31]

5. Kleindorfer D, Panagos P, Pancioli A, et al. Incidence and short-term prognosis of transient ischemic attack in a population-based study. *Stroke; a journal of cerebral circulation* 2005;36(4):720-3. doi: 10.1161/01.STR.0000158917.59233.b7 [published Online First: 2005/02/26]

6. Rothwell PM, Coull AJ, Giles MF, et al. Change in stroke incidence, mortality, case-fatality, severity, and risk factors in Oxfordshire, UK from 1981 to 2004 (Oxford Vascular Study). *Lancet* 2004;363(9425):1925-33. doi: 10.1016/s0140-6736(04)16405-2 [published Online First: 2004/06/15]

7. Coull AJ, Lovett JK, Rothwell PM. Population based study of early risk of stroke after transient ischaemic attack or minor stroke: implications for public education and organisation of services. *BMJ (Clinical research ed)* 2004;328(7435):326. doi: 10.1136/bmj.37991.635266.44 [published Online First: 2004/01/28]

8. Lovett JK, Dennis MS, Sandercock PA, et al. Very early risk of stroke after a first transient ischemic attack. *Stroke; a journal of cerebral circulation* 2003;34(8):e138-40. doi: 10.1161/01.str.0000080935.01264.91 [published Online First: 2003/07/12]

9. Rothwell PM, Giles MF, Chandratheva A, et al. Effect of urgent treatment of transient ischaemic attack and minor stroke on early recurrent stroke (EXPRESS study): a prospective population-

For peer review only

based sequential comparison. *Lancet* 2007;370(9596):1432-42. doi: 10.1016/s0140-6736(07)61448-2 [published Online First: 2007/10/12]

10. Rothwell PM, Giles MF, Flossmann E, et al. A simple score (ABCD) to identify individuals at high early risk of stroke after transient ischaemic attack. *Lancet* 2005;366(9479):29-36. doi: 10.1016/s0140-6736(05)66702-5 [published Online First: 2005/07/05]

11. Intercollegiate Stroke Working Party. National clinical guideline for stroke. London: Royal College of Physicians, 2012.

12. National Institute for Health and Clinical Excellence (NICE). Diagnosis and intial management of acute stroke and transient ischemic attack (TIA). NICE clinical guideline, 2008.

13. Royal College of Physicians. Sentinel Stroke National Audit Programme (SSNAP) Acute Organisational Audit Report 2014, 2014.

14. Brazzelli M, Shuler K, Quayyum Z, et al. Clinical and imaging services for TIA and minor stroke: results of two surveys of practice across the UK. *BMJ open* 2013;3(8) doi: 10.1136/bmjopen-2013-003359 [published Online First: 2013/08/10]

15. Sheppard JP, Mellor RM, Bailey SM, et al. Protocol for an observation and implementation study investigating optimisation of the management of stroke and transient ischaemic attack (TIA). *BMJ open* 2012;2(3) doi: 10.1136/bmjopen-2012-001430 [published Online First: 2012/06/27]

16. Mant J, Barton P, McManus RJ, et al. What is the Optimum Model of Service Delivery for Transient Ischaemic Attack?: National Co-ordinating Centre for NHS Service Delivery and Organisation R&D (NCCSDO), 2008.

17. Lasserson DS, Chandratheva A, Giles MF, et al. Influence of general practice opening hours on delay in seeking medical attention after transient ischaemic attack (TIA) and minor stroke:

For peer review only

prospective population based study. *BMJ (Clinical research ed)* 2008;337:a1569. doi: 10.1136/bmj.a1569 [published Online First: 2008/09/20]

18. Rothwell PM, Eliasziw M, Gutnikov SA, et al. Endarterectomy for symptomatic carotid stenosis in relation to clinical subgroups and timing of surgery. *Lancet* 2004;363(9413):915-24. doi: 10.1016/s0140-6736(04)15785-1 [published Online First: 2004/03/27]

19. Tsivgoulis G, Stamboulis E, Sharma VK, et al. Multicenter external validation of the ABCD2 score in triaging TIA patients. *Neurology* 2010;74(17):1351-7. doi: 10.1212/WNL.0b013e3181dad63e [published Online First: 2010/04/28]

20. Asimos AW, Johnson AM, Rosamond WD, et al. A multicenter evaluation of the ABCD2 score's accuracy for predicting early ischemic stroke in admitted patients with transient ischemic attack. *Annals of emergency medicine* 2010;55(2):201-10.e5. doi: 10.1016/j.annemergmed.2009.05.002 [published Online First: 2009/06/27]

21. Collaborative meta-analysis of randomised trials of antiplatelet therapy for prevention of death, myocardial infarction, and stroke in high risk patients. *BMJ (Clinical research ed)* 2002;324(7329):71-86. [published Online First: 2002/01/12]

22. Aguilar MI, Hart R. Oral anticoagulants for preventing stroke in patients with non-valvular atrial fibrillation and no previous history of stroke or transient ischemic attacks. *The Cochrane database of systematic reviews* 2005(3):Cd001927. doi: 10.1002/14651858.CD001927.pub2 [published Online First: 2005/07/22]

23. Baigent C, Keech A, Kearney PM, et al. Efficacy and safety of cholesterol-lowering treatment: prospective meta-analysis of data from 90,056 participants in 14 randomised trials of statins. *Lancet* 2005;366(9493):1267-78. doi: 10.1016/s0140-6736(05)67394-1 [published Online First: 2005/10/11]

For peer review only

24. Ebrahim S, Harwood R. Stroke epidemiology, evidence, and clinical practice. Oxford University Press: Oxford 1999.

25. MacMahon S. BP and the risks of CV disease. In: Swales J, ed. Textbook of hypertension. Oxford: Blackwell Scientific Publications 1994.

26. Wolf PA, Abbott RD, Kannel WB. Atrial fibrillation as an independent risk factor for stroke: the Framingham Study. *Stroke; a journal of cerebral circulation* 1991;22(8):983-8. [published Online First: 1991/08/01]

27. Department of Health. 2011–12 Reference Costs 2013 [Available from: https://www.gov.uk/government/publications/2011-12-reference-costspublication accessed June 2013.

28. Curtis L. Unit Costs in Health and Social Care 2011: Personal Social Services Research Unit (PSSRU); 2012 [Available from: http://www.pssru.ac.uk/project-pages/unit-costs/2011/index.php. accessed November 2012.

29. British National Formulary (BNF). Pharmaceutical Press 2012 [Available from: https://www.bnf.org/ accessed November 2012.

30. Wardlaw JM, Brazzelli M, Chappell FM, et al. ABCD2 score and secondary stroke prevention: Meta-analysis and effect per 1,000 patients triaged. *Neurology* 2015;85(4):373-80. doi: 10.1212/wnl.0000000000001780

31. Penaloza-Ramos MC, Sheppard JP, Jowett S, et al. Cost-effectiveness of optimizing acute stroke care services for thrombolysis. *Stroke; a journal of cerebral circulation* 2014;45(2):553-62. doi: 10.1161/strokeaha.113.003216 [published Online First: 2014/01/05]

32. Earnshaw SR, McDade C, Chapman AM, et al. Economic impact of using additional diagnostic tests to better select patients with stroke for intravenous thrombolysis in the United Kingdom.

For peer review only

Clinical therapeutics 2012;34(7):1544-58. doi: 10.1016/j.clinthera.2012.05.004 [published Online First: 2012/06/15]

33. Dirks M, Baeten SA, Dippel DW, et al. Real-life costs and effects of an implementation program to increase thrombolysis in stroke. *Neurology* 2012;79(6):508-14. doi: 10.1212/WNL.0b013e31826356bf [published Online First: 2012/07/13]

34. Barton M, McClean S, Gillespie J, et al. Is it beneficial to increase the provision of thrombolysis?-- a discrete-event simulation model. *QJM : monthly journal of the Association of Physicians* 2012;105(7):665-73. doi: 10.1093/qjmed/hcs036 [published Online First: 2012/03/03]

35. Joshi JK, Ouyang B, Prabhakaran S. Should TIA patients be hospitalized or referred to a same-day clinic?: a decision analysis. *Neurology* 2011;77(24):2082-8. doi: 10.1212/WNL.0b013e31823d763f [published Online First: 2011/11/19]

36. Nguyen-Huynh MN, Johnston SC. Is hospitalization after TIA cost-effective on the basis of treatment with tPA? *Neurology* 2005;65(11):1799-801. doi: 10.1212/01.wnl.0000187067.93321.fa [published Online First: 2005/12/14]

37. Buisman LR, Tan SS, Nederkoorn PJ, et al. Hospital costs of ischemic stroke and TIA in the Netherlands. *Neurology* 2015;84(22):2208-15. doi: 10.1212/wnl.0000000000001635 [published Online First: 2015/05/03]

38. Ranta A, Lichtman JH. Health economics of cerebrovascular disease: Can we do better? *Neurology* 2015;84(22):2204-5. doi: 10.1212/wnl.0000000000001651 [published Online First: 2015/05/03]

For peer review only

39. Sanders LM, Cadilhac DA, Srikanth VK, et al. Is nonadmission-based care for TIA patients cost-effective?: A microcosting study. *Neurology: Clinical Practice* 2015;5(1):58-66. doi: 10.1212/cpj.0000000000000095

40. Beech P, Greenhalgh J, Thornton M, et al. A survey of the practice of stroke doctors in developing transient ischaemic attack services in the UK. *Journal of evaluation in clinical practice* 2007;13(3):395-9. doi: 10.1111/j.1365-2753.2006.00714.x [published Online First: 2007/05/24]

41. Ranta A, Barber PA. Transient ischemic attack service provision: A review of available service models. *Neurology* 2016 doi: 10.1212/wnl.0000000000002339

For peer review only

Figure legends

Figure 1. Example survival curves and daily hazard rates for risk of major stroke in high and low risk patients.

High risk patient: male age 70-74, SBP 156mmHg, Speech disturbance without weakness, Duration of symptoms 60+ mins, not diabetic (resulting in ABCD and $ABCD^2$ scores of 5), undiagnosed AF, cholesterol 5.8. Low risk patient: male age 70-74, SBP 115mmHg, Speech disturbance without weakness, Duration of symptoms 0-9 mins, not diabetic (resulting in ABCD and $ABCD^2$ of scores 2), AF on warfarin, cholesterol 6.0.

Figure 2. Outcomes of service provision in terms of clinic appointment utilisation and guideline breaches.

OP = outpatient appointment; TIA = transient ischemic attack; LR = low risk; HR = high risk. Original and modified services (first two bars at each hospital) were those which were actually implemented in each hospital. The remaining weekend services are hypothetical. *Hospital 1 included 16 routine clinics, 5 days per week (original service), 17 in the modified service and 19 in the modified service with weekend working. †Hospital 2 included 12 routine clinics, 3 days per week (original service), and 14 in the modified service (which also included weekend working).

For peer review only

For peer review only - http://bmjopen.bmj.com/site/about/guidelines.xhtml

Tables

Table 1. Patient characteristics as modelled

**Patient characteristics**	
Age (mean±SD) (years)	74 ± 12
Gender (% female)	240 (48.9%)
Systolic blood pressure (mean ± SD) (mmHg)	
TIA mimic	143 ± 26
TIA	147 ± 22
Minor stroke	144 ± 18
Overall mean	145 ± 24
Final Diagnosis (Actual condition)	
High risk TIA	116 (23.6%)
Low risk TIA	46 (9.4%)
TIA mimic	294 (60.0%)
Minor stroke	34 (7.0%)
*Patients referral to hospital via their GP according to diagnosis**	
High risk TIA	84 (72.3%)
Low risk TIA/ TIA mimic	299 (87.9%)
Minor stroke	20 (57.4%)
Overall mean	402 (82.1%)

SD = Standard deviation; TIA = Transient ischemic attack; GP = general practitioner. *Expected numbers are rounded to the nearest integer, the apparent anomaly with the addition results from this rounding. High risk patients defined as an $ABCD^2$ score of >4

For peer review only

Table 2. Pattern of outpatient clinics for suspected TIA based on actual service provision at participating hospitals during patient recruitment period.

**Hospital and service**		**Number of routine clinic slots available by day of the week**					**Total clinics per year**	**Details of clinic allocation within the model**
		Mon	***Tues***	***Wed***	***Thur***	***Fri***		
Hospital 1	Original service*	2	4	4	4	2	724	All patients are assigned to the next available clinic slots in order of referral. Where two referrals are made in one day, high risk patients are given priority.
Hospital 1	Modified service*	4	4	2	3	4	769	Where two referrals are made in one day, high patients are given priority. One slot is reserved at the end of each clinic for high risk referrals. If the next high risk clinic slot is not within 24 hours of referral, an additional slot is made available (up to 1 per day). All other patients are assigned to the next available clinic slots in order of referral.
Hospital 2	Original service†	6	4	2	0	0	624	Patients are assigned to the next available outpatient clinic. Those high risk patients who cannot be seen within 24 hours are admitted (including those presenting at weekends).
Hospital 2	Modified service†	4 or 6‡	0	4	0	4	676	All high risk patients are seen on the ward as outpatients as required (including at weekends). Patients referred before 10am are seen at 5pm on the same day, patients referred after 10am are seen at 10am on the following day. All low risk patients seen at the next available clinic in order of referral.

*Clinics are divided among 4 specialists, each of whom were absent for approximately 7 weeks a year (annual leave). These clinics are assumed not to take place if the specialist is absent.

†Specialists were absent for approximately 7 weeks a year (annual leave). All absent clinicians were replaced by a specialist from another site within the Trust.

‡There is a 50% probability each week that either 4 or 6 clinic slots will be available.

For peer review only - http://bmjopen.bmj.com/site/about/guidelines.xhtml

Table 3. Costs, resource utilisation and outcomes (per year) of modifying TIA service provision in hospital 1.

	**Original service (16 clinic slots per week)**	**Modified service (17 clinic slots per week)**	**Weekend service + 17 clinic slots per week)**	**Weekend service + 15 clinic slots per week)**	**Weekend service + 13 clinic slots per week)**
Days operating per week	5 days	5 days	7 days	7 days	7 days
Total number of patients presenting	491	490	490	491	491
Cost of clinics used & unused	£340,000	£361,000	£366,000	£346,000	£325,000
Major strokes post TIA (mean, 99% CI)*	10.6 (10.4-10.8)	10.7 (10.5-10.9)	10.6 (10.4-10.8)	10.8 (10.6-11.0)	10.6 (10.4-10.8)
Post-stroke deaths (mean, 99% CI)*	3.0 (2.9-3.1)	3.0 (2.9-3.1)	3.0 (2.9-3.1)	3.1 (3.0-3.2)	3.0 (2.9-3.1)
Number of high risk breaches† (%)	103 (69%)	32 (21%)	6 (4%)	7 (5%)	8 (5%)
Number of low risk breaches‡ (%)	7 (2%)	4 (1%)	3 (1%)	13 (4%)	75 (22%)
Time from referral to clinic appointment for high risk patients in days (median, IQR)	1.15 (0.93-2.88)	0.85 (0.17-0.99)	0.68 (0.16-0.93)	0.70 (0.15-0.93)	0.86 (0.16-0.99)

TIA = transient ischemic attack; CI = confidence intervals; IQR = inter-quartile range. Of the ~490 patients in the model, 340 are considered low risk (294 TIA mimic; 46 low risk TIA) and 150 are considered high risk (116 high risk TIA; 34 minor stroke). *Point estimate and 99% quasi confidence interval reflecting the uncertainty from sampling in the model, not any uncertainty in model parameters. 99% was chosen because of multiple values were compared. †High risk breaches were defined as high risk patients not seen by a specialist within 24 hours of initial clinic referral.[12] ‡ Low risk breaches were defined as low risk patients not seen by a specialist within 7 days of initial clinic referral.

For peer review only - http://bmjopen.bmj.com/site/about/guidelines.xhtml

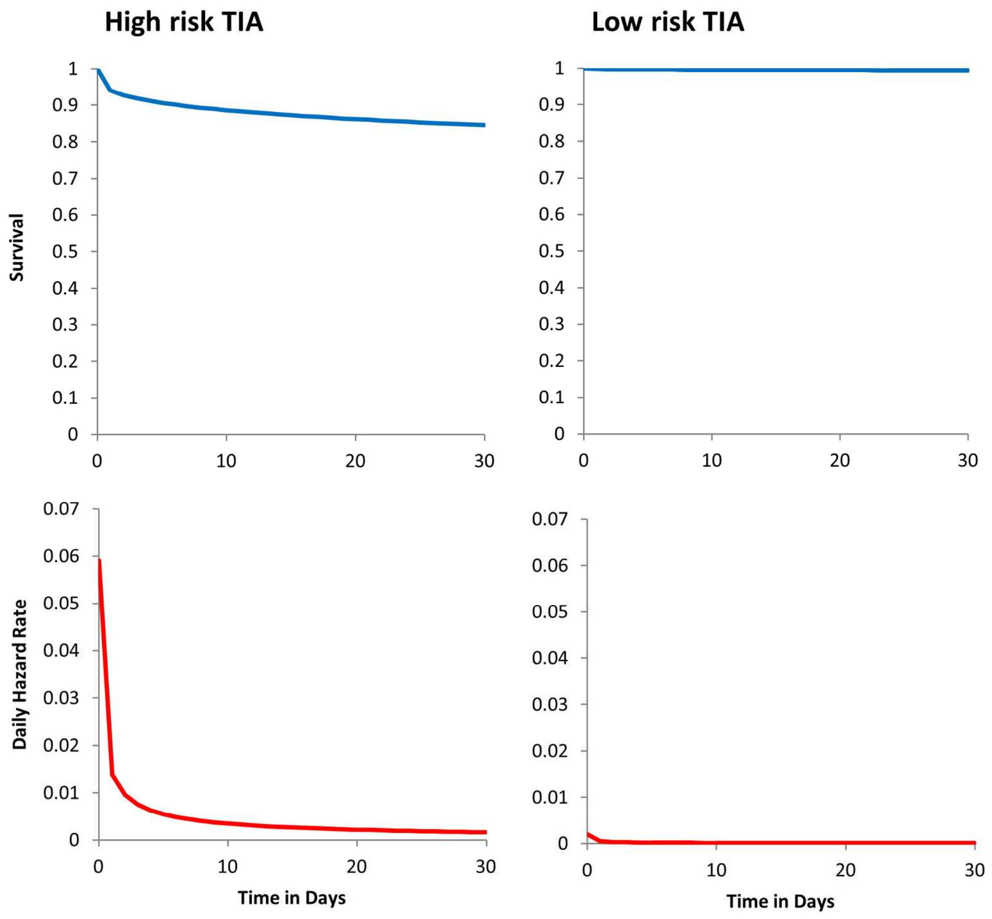

Figure 1. Example survival curves and daily hazard rates for risk of disabling stroke in high and low risk patients.

High risk patient: male age 70-74, SBP 156mmHg, Speech disturbance without weakness, Duration of symptoms 60+ mins, not diabetic (resulting in ABCD and ABCD2 scores of 5), undiagnosed AF, cholesterol 5.8. Low risk patient: male age 70-74, SBP 115mmHg, Speech disturbance without weakness, Duration of symptoms 0-9 mins, not diabetic (resulting in ABCD and ABCD2 of scores 2), AF on warfarin, cholesterol 6.0.

172x160mm (300 x 300 DPI)

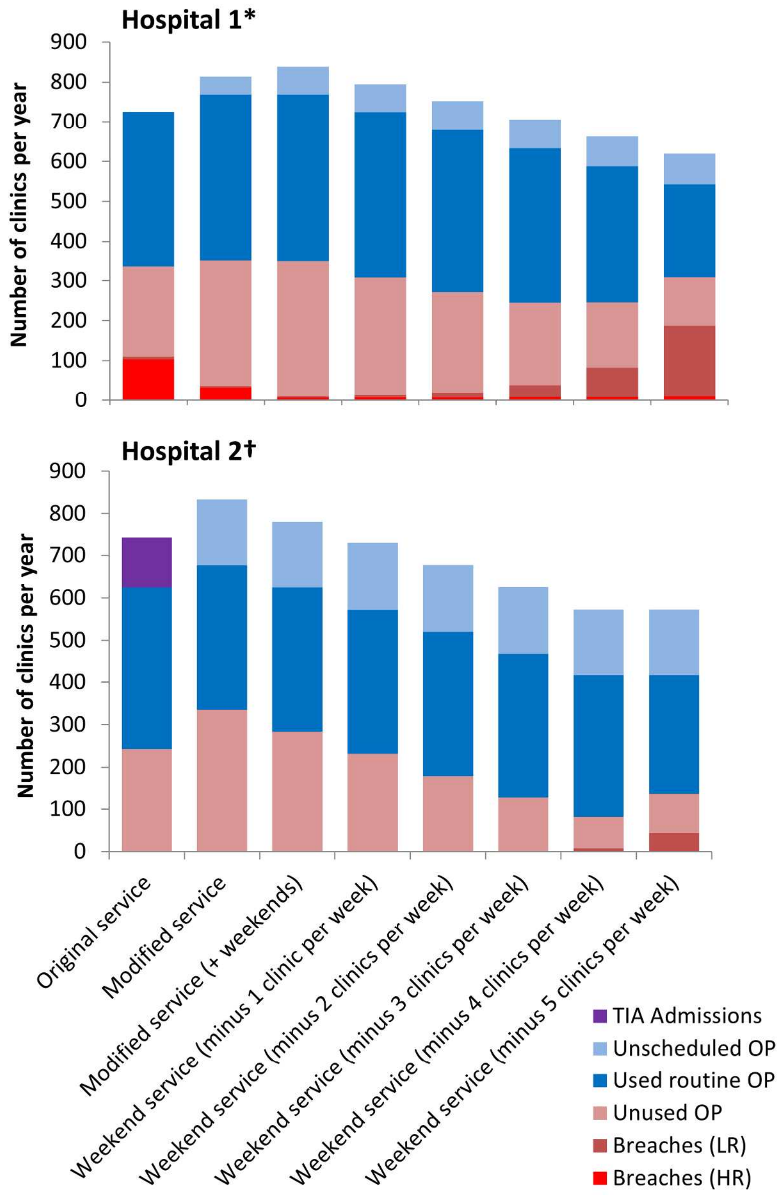

Figure 2. Outcomes of service provision in terms of clinic appointment utilisation and guideline breaches.

OP = outpatient appointment; TIA = transient ischemic attack; LR = low risk; HR = high risk. Original and modified services (first two bars at each hospital) were those which were actually implemented in each hospital. The remaining weekend services are hypothetical. *Hospital 1 included 16 routine clinics, 5 days per week (original service), 17 in the modified service and 19 in the modified service with weekend working. †Hospital 2 included 12 routine clinics, 3 days per week (original service), and 14 in the modified service (which also included weekend working).

172x260mm (300 x 300 DPI)

For peer review only

Online data supplement

Contents

1. **Extended methods**
2. **eFigure 1.** Care pathway for patients presenting with symptoms of transient ischemic attack
3. **eTable 1.** Sources of data included in the model
4. **eTable 2.** Resource unit costs included in the service provision model
5. **eTable 3.** Costs, resource utilisation and outcomes (per year) of modifying TIA service provision in hospital 2.
6. **References**

For peer review only

Extended methods

Setting and service design

The model was designed to assess the costs and clinical consequences arising from patients suspected of suffering an acute TIA and referred to two large urban hospitals in the West Midlands region of the UK. Both hospitals ran a specialist TIA outpatient service catering for approximately 500 patients with suspected stroke and TIA every year. The model parameters were defined by the characteristics of individuals attending these services and recruited to an observational study[1] during 2011 (table 1).

The availability of specialist TIA clinics was modelled on the basis of existing service provision at participating hospital sites during the study period. Patients could either be seen in traditional outpatient clinics, admitted, or seen on the ward on an outpatient basis. The original services were designed within the confines of available clinical staff and a lack of specialist cover at the weekends. Services were redesigned at each site during the study period and the impact of these changes was examined by modelling both the original and modified service. The number and distribution of clinics in each service are described in Table 2.

The impact of further adjustments to the modified service at each site was modelled to replicate the following scenarios:

a) The addition of high risk clinic slots for patients presenting on a Saturday and Sunday
b) Including a weekend service but reducing the number of routine weekday clinics, by up to 5 clinic slots (one patient per slot) per week.

Overview of the model

A discrete event simulation model was programmed in Delphi version 4 (Borland, San Francisco, CA, USA). It was adapted from a previously developed model and further detail regarding the general modelling framework can be found in the original report.[2] Briefly, the model generated a number of possible (virtual) patient histories which began at the onset of TIA (or TIA like) symptoms and followed the patient along the clinical pathway from initial presentation to referral, specialist TIA clinic attendance, treatment and lifetime follow-up for subsequent stroke morbidity and mortality. An essential feature of the model was that patients were sharing limited resources in the form of routine clinics.

For peer review only

The model estimated the impact of existing service provision, the effect of recent service change and the impact of further alterations to clinic availability on service costs/provision and subsequent stroke events, from a health services perspective.

Clinical pathways in the model

Following onset of an initial event, patients were assumed to contact either their general practitioner (GP) or attend the emergency department (ED) (eFigure 1, online supplemental material). The probability that a patient would choose a specific route was dependent on the type of patient (high/low risk), the time of day, and day of week, estimated using data from the Oxford Vascular Study.[3] Time taken to reach the service depended on the route chosen; where patients directly attended the ED, the time was sampled from a Weibull distribution fitted to data from the Newcastle Stroke Service.[2] Patients attending their GP were assumed to arrive within daytime clinic hours.[3] Following this initial contact with a healthcare professional, patients were referred to a specialist TIA outpatient clinic, seen on the ward (as an outpatient) or admitted as an inpatient. The type of referral was dependant on the specific hospital service provision, clinic availability and the level of risk of the attending patient, defined according to the $ABCD^2$ score.[4] During clinic attendance, it was assumed that the appropriate treatment would be initiated (i.e. blood pressure lowering, cholesterol lowering or antiplatelet therapy in accordance with guidelines) and a small proportion of patients (4.1%) would be deemed suitable for carotid endarterectomy.[5,6] Patients were assumed to take this treatment as prescribed and gain the full benefit in terms of stroke prevention.

Model population

All patients in the model were hypothetical, although they were based on data from real patients and hospitals. Overall rates of patient presentation were based on initial runs of the model and ensured a realistic distribution of final diagnoses which represented the study sample, previous literature[7] and expert opinion (60% TIA mimics, 33% genuine TIA, and 7% minor stroke). High and low risk TIAs were defined according to the $ABCD^2$ score.[4] In the absence of robust data for the distribution of new referrals through the week, it was assumed that new cases arrived uniformly.

Due to missing risk data in the sample population, the observed ratio of low risk/high risk TIA patients used as basecase in the model was supplemented in sensitivity analyses by estimates derived from those reported in previous studies[4,8,9] and the experience of stroke physicians from centres across the UK [personal communications, D Sims, G Ford and C Roffe]. The range of these estimates tested in this sensitivity analysis for impact on the model results was: 2.5:1 (high:low risk; base-case) to 1:1, 5:1 and 7:1.[4,8,9]

Follow-up and risk of repeat events

Hypothetical patients remained in the model until one year from symptom onset, after which the increased risk of repeat event returns close to normal,[10,11] unless they died or suffered a non-fatal disabling stroke. No distinction was made in the model between fatal strokes and non-fatal disabling strokes: these were labelled "major strokes". The risk of a repeat event (TIA or stroke) was dependent on the type of initial event (minor stroke, true TIA or mimic), the $ABCD^2$ based risk prediction of a subsequent event and other relevant risk factors such as age, presence of atrial fibrillation and medication prescribed.[4,6,12-17] Following a minor (non-disabling) stroke, patients remained in the model, but with an additional risk of mortality that could be reduced by appropriate treatment. Additional deaths from this cause were estimated and labelled in the model outputs as "post-stroke deaths". Modelled outputs are therefore derived from risk profile of the hypothetical patients, adjusting for the effects of treatments which would start at varying times in the different scenarios. Risks were modelled using a Weibull distribution for the time to event which allowed for a substantially increased risk in the short term, followed by a decreasing risk over time.[18] Examples of the modelled risk for two patients (with high and low risk characteristics) are given in figure 1.

The model was run 100 times for a total simulated time of 12 years in each run: the first year of the run was regarded as a "warm up" period to allow the system to reach a steady state before collecting data. Patients entering during the next 10 years were included in the model results, with the final year being necessary to allow for a one-year follow up time for all included patients. Results for patients entering the model during this last "follow up" period were not included, but such patients were modelled to ensure that the constraints on clinic availability were maintained. Results are presented in terms of annual costs and other outcomes.

Costs and outcomes

The model included costs of any GP visit (from presentation to referral), or transport by ambulance to the ED and ED attendance, outpatient clinics or hospital admission for TIA and stroke, surgery and therapy (eTables 1 and 2, online data supplement). Pre-hospital and treatment related costs were the same for all options considered in this study since the focus was to compare comparative differences between modelled services caused by different clinic configurations. Costs included in the model were taken from a combination of NHS reference costs,[19,20] and drug costs from the British National Formulary.[21] The price year for all costs was 2011-12.

The primary outcome was the number of expected major strokes occurring post TIA, based on risk analysis. Secondary outcomes included the overall service costs per year and attainment of national

For peer review only - http://bmjopen.bmj.com/site/about/guidelines.xhtml

targets for TIA service provision. Target attainment was defined as the number of high and low risk 'breaches' which occurred in each service per year: high risk breaches were defined as a high risk patient not seen by a specialist within 24 hours of initial referral.[5,22] Low risk breaches were defined as low risk patients not seen by a specialist within seven days of referral.[5,22] Further outcomes examined the median time from referral to specialist appointment, the total number of routine outpatient appointments available (used or unused) and any unscheduled outpatient appointments required (where high risk patients were assessed immediately on the ward).

All data are presented as means or medians ± standard deviation (SD), inter-quartile range (IQR) or 99% confidence intervals (CI), chosen because multiple values are compared. Percentages are given for the total population unless otherwise stated.

For peer review only

eFigure 1. Care pathway for patients presenting with symptoms of transient ischemic attack

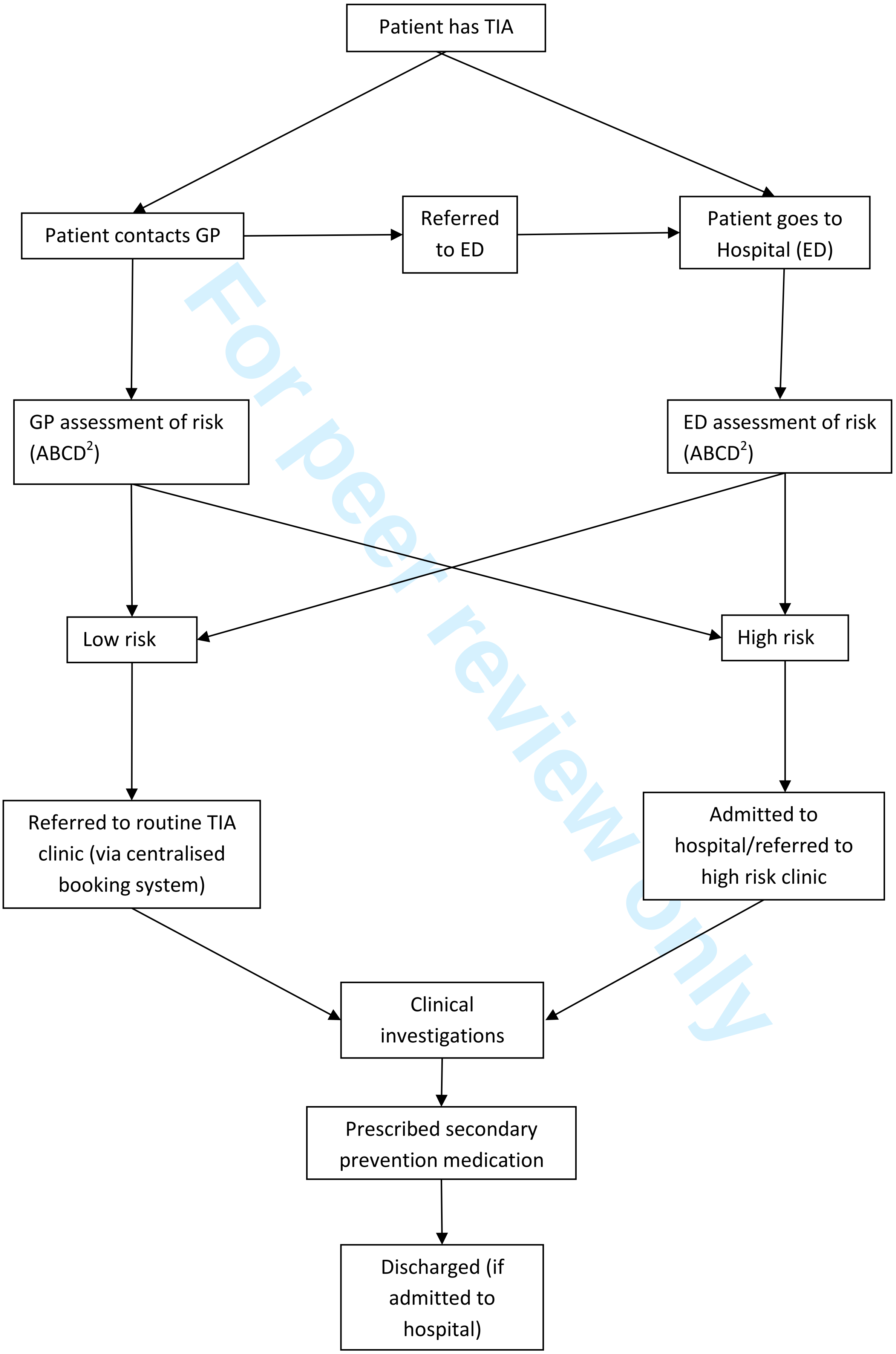

For peer review only

eTable 1. Sources of data included in the model

Model Inputs	Source	References
Schedule of Appointments<br>Casemix (mimic, TIA, minor stroke)	Participating hospital sites, Previous literature and the experience of stroke physicians from centres across the UK (personal communications, D Sims, G Ford and C Roffe)	8,9
Distribution of patient ages	Data from the OXVASC study adjusted to England and Wales population	23
Patient characteristics (gender, components of ABCD^2 score, current medication, AF status, cholesterol level, diabetes conditional on age as appropriate)	Data from the CLAHRC optimisation of the management of stroke and TIA study	1
Patient choice to contact GP or ambulance	OXVASC data	3
Time for patient to reach GP	Assumptions sustained from SDO model	2
Time for patient to reach service using ambulance	Data from Northumbria Ambulance Service	2
"Other cause" death rates	Government Actuary's Department & ONS, Health Statistics Quarterly	24-26
Baseline risk of repeat events	OXVASC data	23
Factors affecting risk of repeat events	Previous literature	12-17,23

OXVASC = OXford VASCular study; CLAHRC = Collaborations for Leadership in Applied Health Research and Care; GP = General Practitioner; ABCD^2 = Age, Blood pressure, Clinical features, Duration and Diabetes; AF = Atrial Fibrillation

For peer review only

eTable 2. Resource unit costs included used in the service provision model.

Item	Cost (£)	Notes	Data source (reference)
Use of an ambulance	230		[19]
General practitioner consultation	33		[20]
Emergency department consultation	143	Category 2 investigation not leading to admitted	[19]
Outpatient appointment	212		[19]
Repeat major stroke	3127		[19]
Assessment for carotid endarterectomy	50	Additional cost of ultrasound only	[19]
Carotid endarterectomy surgery	3923		[19]
Stroke after carotid endarterectomy	3127	As for repeat stroke – small numbers	[19]
Hospital Admission	413		[19]
MRI	92	Applied to 20% of patients at outpatient appointment or hospital admission	[19]
Initiation of antiplatelet therapy*	0.485	300mg loading (from pack of 56 aspirin)	[21]
Antiplatelet mono therapy (per week)*	0.1213	75mg daily from pack of 56 aspirin	[21]
Antiplatelet dual therapy (per week)*	1.0683	Mono plus clopidogrel 75 mg od	[21]
Warfarin (per week)*	0.2150	3mg daily	[21]
Statin (per week)*	0.2925	Simvastatin 40mg daily (from pack of 28)	[21]
Antihypertensive*	0.2025	Bendrofulmethiazide 2.5mg daily	[21]

*Costs taken from the British National Formulary (November 2012) [21]

For peer review only

eTable 3. Costs, resource utilisation and outcomes (per year) of modifying TIA service provision in hospital 2.

	**Original service (12 clinic slots per week)**	**Modified service (14 clinic slots per week)**	**New service (13 clinic slots per week)**	**New service (11 clinic slots per week)**	**New service (9 clinic slots per week)**
Days operating per week	7 days	7 days	7 days	7 days	7 days
Total number of patients presenting	490	490	490	490	490
Cost of clinics used & unused	£368,000	£363,000	£353,000	£329,000	£304,000
Major strokes post TIA (mean, 99% CI)*	10.7 (10.5-10.9)	10.4 (10.2-10.5)	10.7 (10.5-10.9)	10.6 (10.5-10.8)	10.6 (10.4-10.8)
Post-stroke deaths (mean, 99% CI)*	3.0 (2.9-3.1)	3.0 (2.9-3.1)	3.0 (2.9-3.1)	3.1 (3.0-3.2)	3.0 (2.9-3.1)
Number of high risk breaches† (%)	0 (0.0%)	0 (0.0%)	0 (0.0%)	0 (0.0%)	0 (0.0%)
Number of low risk breaches‡ (%)	0 (0.0%)	0 (0.0%)	0 (0.0%)	1 (0.3%)	44 (12.9%)
Time from referral to clinic appointment for high risk patients in days (median, IQR)	0.85 (0.74-0.94)	0.75 (0.08-0.88)	0.75 (0.08-0.88)	0.75 (0.08-0.88)	0.75 (0.08-0.88)

TIA = transient ischemic attack; CI = confidence intervals; IQR = inter-quartile range

Of the ~490 patients in the model, 340 are considered low risk (294 TIA mimic; 46 low risk TIA) and 150 are considered high risk (116 high risk TIA; 34 minor stroke)

*Point estimate and 99% quasi confidence interval reflecting the uncertainty from sampling in the model, not any uncertainty in model parameters. 99% was chosen because of multiple values were compared

†High risk breaches were defined as high risk patients not seen by a specialist within 24 hours of initial clinic referral. These could not occur with the service pattern, as all high risk patients were admitted in the original service, and seen on the ward in the modified service.

‡ Low risk breaches were defined as low risk patients not seen by a specialist within 7 days of initial clinic referral.

For peer review only

References

1. Sheppard JP, Mellor RM, Bailey SM, et al. Protocol for an observation and implementation study investigating optimisation of the management of stroke and transient ischaemic attack (TIA). *BMJ open* 2012; **2**(3).

2. Mant J, Barton P, McManus RJ, Rothwell PM. What is the Optimum Model of Service Delivery for Transient Ischaemic Attack?: National Co-ordinating Centre for NHS Service Delivery and Organisation R&D (NCCSDO), 2008.

3. Lasserson DS, Chandratheva A, Giles MF, Mant D, Rothwell PM. Influence of general practice opening hours on delay in seeking medical attention after transient ischaemic attack (TIA) and minor stroke: prospective population based study. *BMJ (Clinical research ed)* 2008; **337**: a1569.

4. Rothwell PM, Giles MF, Flossmann E, et al. A simple score (ABCD) to identify individuals at high early risk of stroke after transient ischaemic attack. *Lancet* 2005; **366**(9479): 29-36.

5. National Institute for Health and Clinical Excellence (NICE). Diagnosis and intial management of acute stroke and transient ischemic attack (TIA), 2008.

6. Rothwell PM, Eliasziw M, Gutnikov SA, Warlow CP, Barnett HJ. Endarterectomy for symptomatic carotid stenosis in relation to clinical subgroups and timing of surgery. *Lancet* 2004; **363**(9413): 915-24.

7. Brazzelli M, Shuler K, Quayyum Z, et al. Clinical and imaging services for TIA and minor stroke: results of two surveys of practice across the UK. *BMJ open* 2013; **3**(8).

8. Tsivgoulis G, Stamboulis E, Sharma VK, et al. Multicenter external validation of the ABCD2 score in triaging TIA patients. *Neurology* 2010; **74**(17): 1351-7.

9. Asimos AW, Johnson AM, Rosamond WD, et al. A multicenter evaluation of the ABCD2 score's accuracy for predicting early ischemic stroke in admitted patients with transient ischemic attack. *Annals of emergency medicine* 2010; **55**(2): 201-10.e5.

10. Coull AJ, Lovett JK, Rothwell PM. Population based study of early risk of stroke after transient ischaemic attack or minor stroke: implications for public education and organisation of services. *BMJ (Clinical research ed)* 2004; **328**(7435): 326.

11. Lovett JK, Dennis MS, Sandercock PA, Bamford J, Warlow CP, Rothwell PM. Very early risk of stroke after a first transient ischemic attack. *Stroke; a journal of cerebral circulation* 2003; **34**(8): e138-40.

12. Collaborative meta-analysis of randomised trials of antiplatelet therapy for prevention of death, myocardial infarction, and stroke in high risk patients. *BMJ (Clinical research ed)* 2002; **324**(7329): 71-86.

13. Aguilar MI, Hart R. Oral anticoagulants for preventing stroke in patients with non-valvular atrial fibrillation and no previous history of stroke or transient ischemic attacks. *The Cochrane database of systematic reviews* 2005; (3): Cd001927.

14. Baigent C, Keech A, Kearney PM, et al. Efficacy and safety of cholesterol-lowering treatment: prospective meta-analysis of data from 90,056 participants in 14 randomised trials of statins. *Lancet* 2005; **366**(9493): 1267-78.

For peer review only

15. Ebrahim S, Harwood R. Stroke epidemiology, evidence, and clinical practice. Oxford University Press: Oxford; 1999.

16. MacMahon S. BP and the risks of CV disease. In: Swales J, ed. Textbook of hypertension. Oxford: Blackwell Scientific Publications; 1994.

17. Wolf PA, Abbott RD, Kannel WB. Atrial fibrillation as an independent risk factor for stroke: the Framingham Study. *Stroke; a journal of cerebral circulation* 1991; **22**(8): 983-8.

18. Rothwell PM, Giles MF, Chandratheva A, et al. Effect of urgent treatment of transient ischaemic attack and minor stroke on early recurrent stroke (EXPRESS study): a prospective population-based sequential comparison. *Lancet* 2007; **370**(9596): 1432-42.

19. Department of Health. 2011–12 Reference Costs. 2013. https://www.gov.uk/government/publications/2011-12-reference-costspublication (accessed June 2013).

20. Curtis L. Unit Costs in Health and Social Care 2011. 2012. http://www.pssru.ac.uk/project-pages/unit-costs/2011/index.php. (accessed November 2012).

21. British National Formulary (BNF). Pharmaceutical Press. 2012. https://www.bnf.org/ (accessed November 2012).

22. Intercollegiate Stroke Working Party. National clinical guideline for stroke. London: Royal College of Physicians, 2012.

23. Rothwell PM, Coull AJ, Giles MF, et al. Change in stroke incidence, mortality, case-fatality, severity, and risk factors in Oxfordshire, UK from 1981 to 2004 (Oxford Vascular Study). *Lancet* 2004; **363**(9425): 1925-33.

24. Government Actuary's Department. Interim life tables. http://www.gad.gov.uk/, 2006.

25. Health Statistics Quarterly. Deaths by age, sex and underlying cause, 2005 registrations. http://www.statistics.gov.uk/statbase/, 2007.

26. Office for National Statistics. Interim Life Tables for England. http://www.ons.gov.uk/ons/publications/index.html, 2011.

For peer review only

CHEERS Checklist

Items to include when reporting economic evaluations of health interventions

The **ISPOR CHEERS Task Force Report**, *Consolidated Health Economic Evaluation Reporting Standards (CHEERS)—Explanation and Elaboration: A Report of the ISPOR Health Economic Evaluations Publication Guidelines Good Reporting Practices Task Force*, provides examples and further discussion of the 24-item CHEERS Checklist and the CHEERS Statement. It may be accessed via the *Value in Health* or via the ISPOR Health Economic Evaluation Publication Guidelines – CHEERS: Good Reporting Practices webpage: http://www.ispor.org/TaskForces/EconomicPubGuidelines.asp

Section/item	Item No	Recommendation	Reported on page No/ line No
**Title and abstract**			
Title	1	Identify the study as an economic evaluation or use more specific terms such as "cost-effectiveness analysis", and describe the interventions compared.	
Abstract	2	Provide a structured summary of objectives, perspective, setting, methods (including study design and inputs), results (including base case and uncertainty analyses), and conclusions.	
**Introduction**			
Background and objectives	3	Provide an explicit statement of the broader context for the study. Present the study question and its relevance for health policy or practice decisions.	
**Methods**			
Target population and subgroups	4	Describe characteristics of the base case population and subgroups analysed, including why they were chosen.	
Setting and location	5	State relevant aspects of the system(s) in which the decision(s) need(s) to be made.	
Study perspective	6	Describe the perspective of the study and relate this to the costs being evaluated.	
Comparators	7	Describe the interventions or strategies being compared and state why they were chosen.	
Time horizon	8	State the time horizon(s) over which costs and consequences are being evaluated and say why appropriate.	
Discount rate	9	Report the choice of discount rate(s) used for costs and outcomes and say why appropriate.	
Choice of health outcomes	10	Describe what outcomes were used as the measure(s) of benefit in the evaluation and their relevance for the type of analysis performed.	
Measurement of effectiveness	11a	*Single study-based estimates:* Describe fully the design features of the single effectiveness study and why the single study was a sufficient source of clinical effectiveness data.	

For peer review only

For peer review only - http://bmjopen.bmj.com/site/about/guidelines.xhtml

For peer review only

	11b	*Synthesis-based estimates:* Describe fully the methods used for identification of included studies and synthesis of clinical effectiveness data.	
Measurement and valuation of preference based outcomes	12	If applicable, describe the population and methods used to elicit preferences for outcomes.	
Estimating resources and costs	13a	*Single study-based economic evaluation:* Describe approaches used to estimate resource use associated with the alternative interventions. Describe primary or secondary research methods for valuing each resource item in terms of its unit cost. Describe any adjustments made to approximate to opportunity costs.	
	13b	*Model-based economic evaluation:* Describe approaches and data sources used to estimate resource use associated with model health states. Describe primary or secondary research methods for valuing each resource item in terms of its unit cost. Describe any adjustments made to approximate to opportunity costs.	
Currency, price date, and conversion	14	Report the dates of the estimated resource quantities and unit costs. Describe methods for adjusting estimated unit costs to the year of reported costs if necessary. Describe methods for converting costs into a common currency base and the exchange rate.	
Choice of model	15	Describe and give reasons for the specific type of decision-analytical model used. Providing a figure to show model structure is strongly recommended.	
Assumptions	16	Describe all structural or other assumptions underpinning the decision-analytical model.	
Analytical methods	17	Describe all analytical methods supporting the evaluation. This could include methods for dealing with skewed, missing, or censored data; extrapolation methods; methods for pooling data; approaches to validate or make adjustments (such as half cycle corrections) to a model; and methods for handling population heterogeneity and uncertainty.	
**Results**			
Study parameters	18	Report the values, ranges, references, and, if used, probability distributions for all parameters. Report reasons or sources for distributions used to represent uncertainty where appropriate. Providing a table to show the input values is strongly recommended.	
Incremental costs and outcomes	19	For each intervention, report mean values for the main categories of estimated costs and outcomes of interest, as well as mean differences between the comparator groups. If applicable, report incremental cost-effectiveness ratios.	
Characterising uncertainty	20a	*Single study-based economic evaluation:* Describe the effects of sampling uncertainty for the estimated incremental cost and incremental effectiveness parameters, together with the impact	

		of methodological assumptions (such as discount rate, study perspective).	
	20b	*Model-based economic evaluation:* Describe the effects on the results of uncertainty for all input parameters, and uncertainty related to the structure of the model and assumptions.	
Characterising heterogeneity	21	If applicable, report differences in costs, outcomes, or cost-effectiveness that can be explained by variations between subgroups of patients with different baseline characteristics or other observed variability in effects that are not reducible by more information.	
**Discussion**			
Study findings, limitations, generalisability, and current knowledge	22	Summarise key study findings and describe how they support the conclusions reached. Discuss limitations and the generalisability of the findings and how the findings fit with current knowledge.	
**Other**			
Source of funding	23	Describe how the study was funded and the role of the funder in the identification, design, conduct, and reporting of the analysis. Describe other non-monetary sources of support.	
Conflicts of interest	24	Describe any potential for conflict of interest of study contributors in accordance with journal policy. In the absence of a journal policy, we recommend authors comply with International Committee of Medical Journal Editors recommendations.	

For consistency, the CHEERS Statement checklist format is based on the format of the CONSORT statement checklist

The **ISPOR CHEERS Task Force Report** provides examples and further discussion of the 24-item CHEERS Checklist and the CHEERS Statement. It may be accessed via the *Value in Health* link or via the ISPOR Health Economic Evaluation Publication Guidelines – CHEERS: Good Reporting Practices webpage: http://www.ispor.org/TaskForces/EconomicPubGuidelines.asp

The citation for the CHEERS Task Force Report is:
Husereau D, Drummond M, Petrou S, et al. Consolidated health economic evaluation reporting standards (CHEERS)—Explanation and elaboration: A report of the ISPOR health economic evaluations publication guidelines good reporting practices task force. Value Health 2013;16:231-50.

For peer review only
